# Lewis Acid-Catalyzed Formal (4+2)-Cycloaddition between Cross-Conjugated Azatrienes and Styrylmalonates: The Way to Functionalized Quinolizidine Precursors

**DOI:** 10.3390/molecules28010088

**Published:** 2022-12-22

**Authors:** Pavel G. Sergeev, Roman A. Novikov, Yury V. Tomilov

**Affiliations:** N. D. Zelinsky Institute of Organic Chemistry, Russian Academy of Sciences, 47 Leninsky Prosp., 119991 Moscow, Russia

**Keywords:** Styrlmalonates, azatrienes, tetrahydropyridines, formal (4+2)-cycloaddition, Lewis acid catalysis, detosylation, nitrogen-containing heterocycles

## Abstract

Quinolizidine and azaphenalene alkaloids are common in nature and exhibit a pharmaceutical activity, which stirs up increased interest in expanding the range of methods for the synthesis of the corresponding derivatives. In this work, we attempted to adapt our previously presented method for the synthesis of tetrahydropyridines to the preparation of potential precursors for these heterocycles as a separate development of a necessary intermediate stage. To this end, we studied the reactions of β-styrylmalonates with *N*-protected cross-conjugated azatrienes in the presence of Sn(OTf)_2_. Moreover, the regioselectivity of the process involving unsymmetrically substituted azatrienes was estimated. The diene character of vinyltetrahydropyridines was studied in detail with the participation of PTAD. Finally, for the Ts-protected highly functionalized vinyltetrahydropyridines synthesized, a detosylation method to give new desired azadiene structures as precursors of the quinolizidine core was suggested.

## 1. Introduction

Quinolizidine motifs are widely distributed in natural products. These structures have long been of interest, primarily in the context of pharmaceuticals. Today, they continue to attract increasing attention from researchers [1,2,3,4,5,6], along with their related azaphenalene alkaloids [7,8,9]. Such acute attention prompts a search for new methods for the synthesis of compounds containing these structural cores. One of the possible strategies for synthesizing quinolizidine structures may involve the functionalization of an appropriate nitrogen-containing monocyclic precursor. We have recently presented a useful method for highly diastereoselective construction of 1,2,3,4-tetrahydropyridines from styrylmalonates [10,11,12,13,14] **1** and conformationally non-rigid 1-azadienes that undergo a formal (4+2)-cycloaddition in the presence of Lewis acids [15]. In order to expand the prospects for the synthetic application of the previously discovered reaction and to switch to quinolizidine precursors, modifying the substrate in such a way as to create the possibility of completing a second ring has been suggested.

**Scheme 1:**
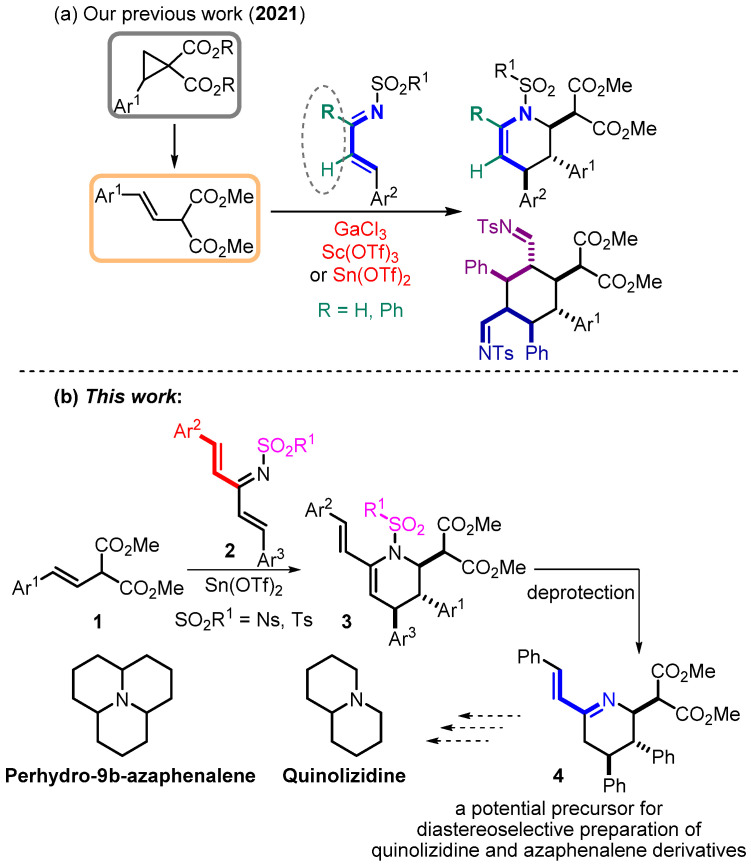
Reactions of styrylmalonates **1** with cross-conjugated azatrienes **2** and further possible transformations of the products.

One of the possible ways to develop this approach involves studying the reaction of cross-conjugated azatrienes **2** as initial substrates (Scheme 1). This method makes it possible to switch to the corresponding vinyltetrahydropyridines **3** containing a diene system, which opens access to further functionalization. Since we are interested in obtaining structures that contain a nodal nitrogen atom, it seems appropriate to “turn” the diene system in its direction by removing the protective group followed by migration of the double bond. It should be noted that such an unusual deprotection seems to be an interesting and promising challenge. It may result in a new class of azadienes **4** that retains the *trans*,*trans*-orientation of the substituents in the cyclic moiety. Compounds of this type can further undergo [4+2]-addition reactions, and thus the strategy makes it possible to use the features of the substrate and form stereocenters in the molecule due to cycloaddition processes that compensate for the loss of the leaving group in terms of atom economy.

## 2. Results and Discussion

**Scheme 2:**
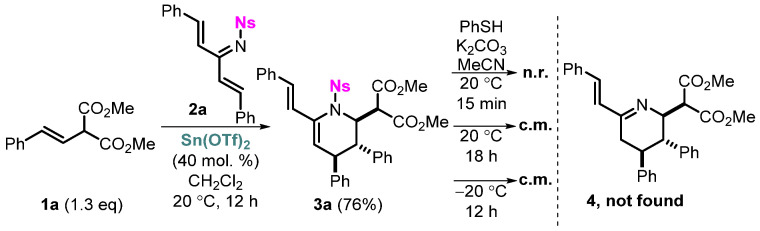
Preparation of vinyltetrahydropyridine **3a** and attempts to remove the nosyl protection.

The implementation of the idea of synthesizing azadienes **4** is closely related to the choice of a removable protective group. In our previous work, substrates containing sulfonyl protective groups such as tosyl (Ts) and nosyl (Ns) were studied because of the availability of well-known and simple methods for the synthesis of the corresponding starting compounds, the acceptor effect that promotes the reaction, and the prominent stability of these groups. These circumstances prompted us to decide first to study the possibility of removing these protective sulfonyl groups in the corresponding substrates. Since the removal of the tosyl group usually requires rather drastic conditions, such as a strongly acidic medium (HBr/AcOH [16], TfOH [17]) or the presence of powerful reducing agents (Na/Hg [18,19], Na/NH_3_ [20], Na(Li)/naphthalene [21,22,23,24,25], SmI_2_ [26,27,28,29,30,31,32,33,34,35,36,37,38,39,40,41,42,43], Mg/MeOH [15,27,44,45,46], etc.), the nosyl group was chosen as the most preferred one. For this purpose, protected azatriene **2a** was obtained and then reacted with styrylmalonate in the presence of Sn(OTf)_2_ as a Lewis acid (Scheme 2). As a result, vinyltetrahydropyridine **3a** was synthesized in a good yield, as those obtained earlier in the reaction with azadienes. Further, a number of attempts were made to remove the nosyl group [47,48]; however, due to side processes, the desired product was not identified in the reaction mixture, as well as any other products in preparative amounts. It is most probable that the thiolate anion is capable of reacting with the azadiene fragment of newly formed denosylated substrate **4** with the occurrence of further side processes. Thus, it was shown that nosyl protection was not an optimal choice for the chosen strategy, so we switched to the synthesis of tosyl derivatives.

**Scheme 3:**
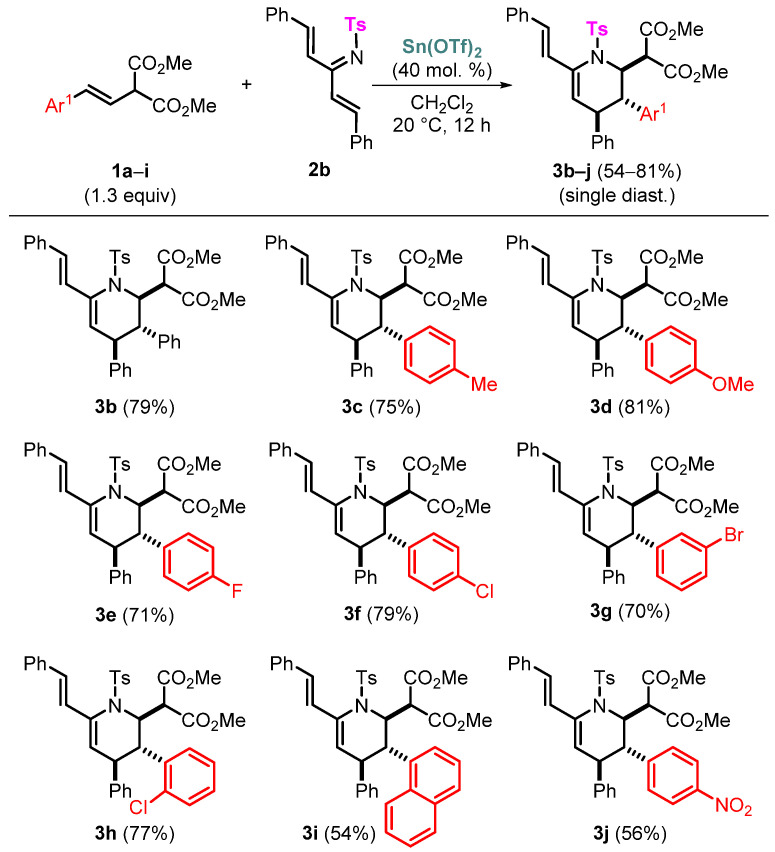
Scope of the reaction for vinyltetrahydropyridines **3**.

To study the scope of the reaction with the participation of sulfonyl-protected azatrienes **2a–c**, we tested a number of starting styrylmalonates **1** (Scheme 3). All the corresponding vinyltetrahydropyridines **3** were obtained as single *trans,trans*-diastereomers. As in the case of azadienes studied in our previous work, the highest yields were achieved for *para*-methyl, -methoxy, and halo substituents in the aryl moiety of styrylmalonates. A noticeable decrease in yields was demonstrated for electron-withdrawing *para*-NO_2_C_6_H_4_- and 1-naphtyl-substituted styrylmalonates. Our attempt to involve an analogous bis(4-MeOC_6_H_4_)-substituted azatriene (see Supplementary Materials) in the reaction failed due to side reactions of the donor substrate in the presence of Sn(OTf)_2_, which was confirmed by mixing these substances in the absence of styrylmalonate; only traces of the desired product were detected in the ^1^H NMR spectrum. It is interesting that compound **3i** showed a partial broadening of a set of signals in the NMR spectra; the same behavior was shown and studied in detail earlier in our previous work for a similar product of the reaction between the same *ortho*-ClC_6_H_4_-styrylmalonate and azadiene bearing a Ph group at the C=N double bond (see [15], Supplementary Materials). As in the case described in the previous work, the signals of some aliphatic (e.g., H(2), H(3), H(4) in the tetrahydropyridine ring) and aromatic protons that are spatially proximate to the *ortho*-ClC_6_H_4_ moiety are broadened, and apparently, those closest to the chlorine atom (especially H(2) and H(4)) undergo a downfield shift by up to 0.8 ppm that is quite noticeable in comparison with the chemical shifts of the corresponding signals of the other obtained tetrahydropyridines. Presumably, this effect can be explained by the proximity and direct influence of the chlorine atom.

**Scheme 4:**
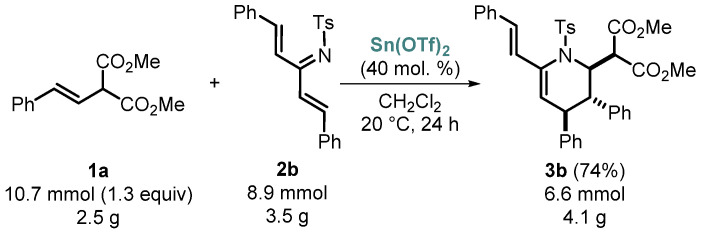
Gram-scale synthesis of vinyltetrahydropyridine **3b**.

To show the potential applications of our synthetic method, we carried out the reaction between styrylmalonate **1a** and azatriene **2b** on a gram scale under standard reaction conditions (Scheme 4). As shown by TLC, in this case, the reaction takes longer to achieve full conversion. Fortunately, the formation of the desired vinyltetrahydropyridine **3b** occurred with only a slightly lower yield in comparison with the milligram-scale synthesis.

A study of the regioselectivity of the reaction in the presence of unsymmetrically substituted azatrienes is an obvious development of this research. To identify the potential effect of electronic and sterical factors, the corresponding azatrienes **2c**,**d** were synthesized and used in the reaction with styrylmalonate **1d** as the model substrate (Table 1). It was shown that the effect of strong electron-donating and electron-withdrawing groups is not sufficient to make the process regioselective (compounds **3k** and **3k′**), but the situation was slightly better in the case of the sterical impact of the 2,6-Cl_2_C_6_H_3_ moiety. This observation prompted us to increase steric hindrance by using a bulkier *ortho*-ClC_6_H_4_ substituent in the starting styrylmalonate **1g**, which resulted in increasing the ratio of regioisomers **3m** and **3m′** up to 4/1 at low temperature (entry 6).

**Scheme 5:**
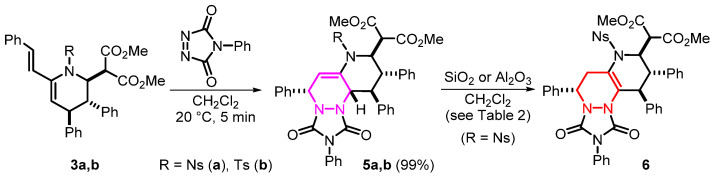
The reaction of vinyltetrahydropyridines **3a**,**b** with PTAD to form adducts **5a**,**b** and subsequent formation of isomer **6**.

The resulting vinyltetrahydropyridines **3** contain a diene moiety that can be involved in [4+2]-cycloaddition reactions and thereby provide access to the implementation of the diene-transmissive hetero Diels–Alder reaction strategy (DTHDA) [49]. To illustrate this approach, reactions of compounds **3a**,**b** with a commonly used dienophile PTAD were carried out (Scheme 5). The process occurs very quickly with almost quantitative yields to give classical adducts **5a**,**b**. Purification of the latter using column chromatography was found to be difficult due to the occurrence of side reactions on silica gel, the products of which were adsorbed and partially decomposed. However, the purity and yield of the compounds obtained make it possible to do without additional purification. Nevertheless, for one of the adducts, **5a**, we attempted to isolate the most stable product of transformation by carrying out the reactions with SiO_2_ as an acidic reagent (Scheme 5, Table 2), and heterocycle **6** was isolated as the only product of double bond migration. Despite the low yield of this compound, it is of interest because a new asymmetric center is induced at a noticeable distance from the initial ones. In view of this, we tried to optimize the conditions for double bond migration, including the replacement of silica gel with neutral Al_2_O_3_, but we failed to achieve an improvement here (Table 2).

The next stage of the work was the selection of the method for the removal of tosyl protection. Vinyltetrahydropyridine **3b** was chosen as a model substrate. It should be noted that the removal of tosyl protection is still an urgent problem of modern organic chemistry. Despite the existence of a set of deprotection methods discussed above, the type of substrate has a very strong influence, which requires careful selection and optimization of conditions in each specific case. We tested a variety of detosylation conditions described in the literature (Table 3). It was shown that the use of SmI_2_ in combination with miscellaneous additives was not effective (entries 1–4). In the case of using the Mg/MeOH system, it was not possible to stop the reduction at the stage of azadiene **4**; the formation of traces of further reduction products were found (entries 5–7). An attempt at detosylation in the presence of TfOH or HBr/AcOH resulted in the formation of a complex mixture of products (entries 8–10). Finally, the formation of the desired azadiene **4** in an acceptable amount was achieved only if sodium naphthalenide was used (entries 11–13). Apparently, the rather low yields of compound **4** are due to the occurrence of side reactions under harsh reducing conditions, which indicates the need for further research to develop a milder approach to the preparation of such compounds. Nevertheless, this result shows that the formation of such complexly functionalized azadienes is possible. This fact opens up the possibility of further study of such substances in reactions with various ene compounds for the formation of the desired quinolizidines.

## 3. Materials and Methods

### 3.1. General Methods

All reagents and solvents used were purchased from Aldrich (St. Louis, MI, USA) or Acros Organics (Geel, Belgium) without additional purification. THF was purified by distillation over Na/benzophenone under an argon atmosphere just prior to use. All operations were performed under an argon atmosphere. Starting styrylmalonates **1** were synthesized from the corresponding D-A cyclopropanes [50,51]. TLC analysis was performed on Silufol chromatographic plates. For preparative chromatography, silica gel 60 (0.040−0.063 mm) was used. ^1^H, ^13^C, and ^15^N NMR spectra were recorded on a Bruker AM-300 (300.1, 75.5, and 30.4 MHz, respectively) spectrometers in CDCl_3_ or CD_2_Cl_2_ containing 0.05% Me_4_Si as the internal standard. Determinations of structures and stereochemistry of obtained compounds and assignments of ^1^H and ^13^C signals were made with the aid of 1D and 2D gradient/non-gradient DEPT-135, COSY, NOESY, HSQC, HMBC, and ^1^H–^15^N HMBC spectra. ^19^F NMR spectra were recorded on a 300 MHz spectrometer (282.4 MHz); standard—CFCl_3_. High-resolution mass spectra were obtained using simultaneous electrospray ionization (ESI) [52]. The melting points were determined using a Kofler hot-stage microscope.

### 3.2. Synthetic Procedures

#### 3.2.1. General Synthetic Procedure and Spectroscopic Data for Azatrienes **2**

A mixture of corresponding substituted 1,4-pentadien-3-one (10 mmol), Et_3_N (6.13 mL, 44 mmol), and *p*-nitrophenylsulfonamide or *p*-toluenesulfonamide (4.0 or 3.4 g, 20 mmol) in CH_2_Cl_2_ (50 mL) was cooled to 0 °C, and titanium tetrachloride (10 mL, 1.0 M solution in CH_2_Cl_2_, 10 mmol) was added dropwise. The reaction mixture was warmed to room temperature with stirring for 7 h, after which the reaction was quenched with saturated aqueous NaHCO_3,_ and the mixture was extracted with CH_2_Cl_2_ (30 mL × 2). The combined extracts were washed with water and brine, dried over MgSO_4_, and concentrated in vacuo. Acetone (20 mL) was added to the residue, left for 10 min, and then the precipitate was filtered off, washed with cold acetone, and dried to afford the azatrienes **2**.

*N-((1E,4E)-1,5-diphenylpenta-1,4-dien-3-ylidene)-4-nitrobenzenesulfonamide* (**2a**)**.** Yellow solid (2.39 g, 57%). M.p. 153–155 °C. ^1^H NMR (300.1 MHz, CDCl_3_): δ 7.29–7.77 (m, 14H, Ph, CH=CH), 8.24 (d, *J* = 9.0 Hz, 2H), 8.38 (d, *J* = 9.0 Hz, 2H) ppm. ^13^C NMR (75.5 MHz, CDCl_3_): δ 122.9 (2=CH), 124.1 (2CH, *m*-Ns), 128.4 (2CH), 128.7 (4CH), 129.1 (6CH), 131.2 (2=CH), 134.5 (2C_q_, Ph), 145.4 (C_q_, Ns), 147.5 (C_q_, Ns), 173.6 (C=N) ppm. HRMS (ESI) *m/z*: calcd for C_23_H_19_N_2_O_4_S^+^ [M+H]^+^ 419.1060; found: 419.1053.

*N-((1E,4E)-1,5-diphenylpenta-1,4-dien-3-ylidene)-4-methylbenzenesulfonamide* (**2b**)**.** Yellow solid (2.36 g, 61%). M.p. 184–185 °C. ^1^H NMR (300.1 MHz, CDCl_3_): δ 2.42 (s, 3H, Ts), 7.24–7.61 (m, 16H, Ar, CH=CH), 7.94 (d, *J* = 8.2 Hz, Ts) ppm. Spectral data are consistent with literature data [49].

*N-((1E,4E)-1-(4-methoxyphenyl)-5-(4-nitrophenyl)penta-1,4-dien-3-ylidene)-4-methylbenzenesulfonamide* (**2c**)**.** Orange solid (2.31 g, 50%). M.p. >250 °C. ^1^H NMR (300.1 MHz, CDCl_3_): δ 2.44 (s, 3H, Me), 3.86 (s, 3H, OMe), 6.93 (d, *J* = 8.7 Hz, 2H, 4-MeOC_6_H_4_), 7.20–8.00 (m, 4H, CH=CH), 7.34 (d, *J* = 8.2 Hz, 2H, Ts), 7.57 (d, *J* = 8.7 Hz, 2H, 4-MeOC_6_H_4_), 7.71 (d, *J* = 8.6 Hz, 2H, 4-NO_2_C_6_H_4_), 7.92 (d, *J* = 8.2 Hz, 2H, Ts), 8.24 (d, *J* = 8.6 Hz, 2H, 4-NO_2_C_6_H_4_) ppm. ^13^C NMR (75.5 MHz, CDCl_3_): δ 21.6 (Me), 55.5 (OMe), 114.6 (2CH, 4-MeOC_6_H_4_), 124.2 (2CH), 127.1 and 127.2 (2=CH), 127.1 (2CH), 128.8 (2CH), 129.5 (2CH), 130.7 (2CH), 138.8 (C_q_), 139.4 (2=CH), 141.1 (C_q_), 143.5 (C_q_), 145.4 (2C_q_), 148.4 (C_q_), 162.4 (C_q_–OMe), 171.4 (C=N) ppm. HRMS (ESI) *m/z*: calcd for C_25_H_23_N_2_O_5_S^+^ [M+H]^+^ 463.1322; found: 463.1323. 

*N-((1E,4E)-1-(2,6-dichlorophenyl)-5-phenylpenta-1,4-dien-3-ylidene)-4-methylbenzenesulfonamide* (**2d**)**.** Yellow solid (2.10 g, 46%). M.p. 167–169 °C. ^1^H NMR (300.1 MHz, CDCl_3_): δ 2.43 (s, 3H, Me), 7.00–8.20 (m,4H, CH=CH), 7.17–7.25 (t, *J* = 8.2 Hz, 1H, 2,6-Cl_2_C_6_H_3_), 7.33 (d, *J* = 8.2 Hz, Ts), 7.38 (d, *J* = 8.2 Hz, 2H, 2,6-Cl_2_C_6_H_3_), 7.38–7.46 (m, 3H, Ph), 7.57–7.66 (m, 2H, Ph), 7.94 (d, *J* = 8.2 Hz, Ts) ppm. ^13^C NMR (75.5 MHz, CDCl_3_): δ 21.6 (Me), 124.5 (2=CH), 127.2 (2CH), 128.7 (br.s, 2CH), 128.8 (2CH), 129.0 (2CH), 129.4 (2CH), 130.0 (CH), 131.0 (CH), 132.5 (=CH), 134.6 (C_q_), 135.1 (C_q_), 137.0 (C_q_), 138.7 (=CH), 143.5 (C_q_), 145.6 (C_q_), 170.8 (C=N) ppm. HRMS (ESI) *m/z*: calcd for C_24_H_20_Cl_2_NO_2_S^+^ [M+H]^+^ 456.0586; found: 456.0574.

#### 3.2.2. General Synthetic Procedure and Spectroscopic Data for Vinyltetrahydropyridines **3**

A solution of styrlmalonate **1a–i** (0.33 mmol, 1.3 eq.), azatriene **2a–e** (0.25 mmol), and Sn(OTf)_2_ (40 mol. %, 42 mg) in dry CH_2_Cl_2_ (3 mL) was stirred 12 h at room temperature or 5 days at -20 °C for azatrienes **2c,d**. Then the reaction mixture without any preliminary work-up was purified by column chromatography on silica gel (petroleum ether–EtOAc 20:1 to 1:1 or benzene–EtOAc, 100:1 to 10:1) to afford title compounds **3a–m,k’–m’**.

*Dimethyl trans,trans-2-(1-((4-nitrophenyl)sulfonyl)-3,4-diphenyl-6-((E)-styryl)-1,2,3,4-tetrahydropyridin-2-yl)malonate* (**3a**)**.** Yellow solid (124 mg, 76%). R_f_ = 0.47 (petroleum ether–EtOAc 3:1). M.p. 127–129 °C. ^1^H NMR (300.1 MHz, CDCl_3_): δ 3.02 (dd, ^3^*J*_3,4_ = 9.5 Hz, ^3^*J*_4,5_ = 3.8 Hz, 1H, H(4)), 3.38 and 3.43 (both s, 2×3H, 2 CO_2_Me), 3.51 (dd, ^3^*J*_3,4_ = 9.5 Hz, ^3^*J*_2,3_ = 6.6 Hz, 1H, H(3)), 3.87 (d, ^3^*J*_2,2′_ = 8.1 Hz, 1H, H(2′)), 5.33 (dd, ^3^*J*_2,2′_ = 8.1 Hz, ^3^*J*_2,3_ = 6.6 Hz, 1H, H(2)), 6.31 (d, ^3^*J*_4,5_ = 3.8 Hz, 1H, H(5)), 6.54 (d, ^3^*J*_1″,2″_ = 16.0 Hz, 1H, H(2″)), 6.79–6.85 (m, 2H, 2×H(*o″*)), 6.84 (d, ^3^*J*_1″,2″_ = 16.0 Hz, 1H, H(1″)), 6.89–6.98 (m, 2H, 2×H(*o’*)), 7.04–7.15 (m, 6H, 2×H(*m’*), H(*p’*), 2×H(*m″*) and H(*p″*)), 7.24–7.37 (m, 5H, 2×H(*o‴*), 2×H(*m‴*) and H(*p‴*)), 8.04 (d, *J* = 8.9 Hz, 2H, 2×H(*o*)), 8.28 (d, *J* = 8.9 Hz, 2H, 2×H(*m*)) ppm. ^13^C NMR (75.5 MHz, CDCl_3_): δ 46.3 (CH(4)), 52.38 and 52.41 (2 CO_2_Me), 55.2 (CH(3)), 56.1 (CH(2′)), 62.3 (CH(2)), 123.8 (2×CH(*m*)), 125.1 (CH(1″)), 126.6 (2×CH(*o‴*)), 126.7 (CH(*p″*)), 127.1 (CH(*p’*)), 127.9 (2×CH(*o″*)), 128.23, 128.27, 128.32, 128.36 and 128.36 (CH(5), 2×CH(*o’*), 2×CH(*m’*), 2×CH(*m″*) and CH(*p‴*)), 128.8 (2×CH(*m‴*)), 129.6 (2×CH(*o*)), 131.1 (CH(2″)), 136.0 (C(*i‴*)), 137.8 (C(6)), 140.2 (C(*i’*)), 141.1 (C(*i″*)), 144.7 (C(*i*)), 150.1 (C(*p*)), 166.7 and 166.8 (2 COO) ppm. HRMS (ESI) *m/z*: calcd for C_36_H_33_N_2_O_8_S^+^ [M+H]^+^ 653.1952; found: 653.1951. 

*Dimethyl trans,trans-2-(3,4-diphenyl-6-((E)-styryl)-1-tosyl-1,2,3,4-tetrahydropyridin-2-yl)malonate* (**3b**)**.** Yellow solid (122 mg, 79%). R_f_ = 0.46 (petroleum ether–EtOAc 3:1). M.p. 91–92 °C. ^1^H NMR (300.1 MHz, CDCl_3_): δ 2.43 (s, 3H, C(*p*)-Me), 2.53 (dd, ^3^*J*_3,4_ = 11.2 Hz, ^3^*J*_4,5_ = 3.9 Hz, 1H, H(4)), 3.11 and 3.49 (both s, 2×3H, 2 CO_2_Me), 3.65 (dd, ^3^*J*_3,4_ = 11.2 Hz, ^3^*J*_2,3_ = 8.3 Hz, 1H, H(3)), 4.06 (d, ^3^*J*_2,2′_ = 6.3 Hz, 1H, H(2′)), 5.17 (dd, ^3^*J*_2,3_ = 8.3 Hz, ^3^*J*_2,2′_ = 6.3 Hz, 1H, H(2)), 6.20 (d, ^3^*J*_4,5_ = 3.9 Hz, 1H, H(5)), 6.62–6.71 (m, 2H, 2×H(*o″*)), 6.75 and 6.78 (two doublets, ^3^*J*_1″,2″_ = 16.3 Hz, 2H, H(1″) and H(2″)), 6.77–6.85 (m, 2H, 2×H(*o’*)), 6.92–7.07 (m, 6H, 2×H(*m’*), H(*p’*), 2×H(*m″*) and H(*p″*)), 7.16–7.24 (m, 1H, H(*p‴*)), 7.25–7.33 (m, 2H, 2×H(*m‴*)), 7.32–7.42 (m, 2H, 2×H(*o‴*)), 7.35 (d, *J* = 8.2 Hz, 2H, 2×H(*m*)), 7.90 (d, *J* = 8.2 Hz, 2H, 2×H(*o*)) ppm. ^13^C NMR (75.5 MHz, CDCl_3_): δ 21.3 (C(*p*)-Me), 46.7 (CH(4)), 51.7 and 52.1 (2 CO_2_Me), 55.4 (CH(3)), 56.1 (CH(2′)), 61.9 (CH(2)), 125.6 (CH(1″)), 126.2 (CH(*p″*)), 126.4 (2×CH(*o‴*)), 126.5 (CH(*p’*)), 127.6 (CH(*p‴*)), 127.7, 127.8 and 127.9 (2×CH(*m’*), 2×CH(*o″*) and 2×CH(*m″*)), 128.0 (2×CH(*o*)), 128.4 (2×CH(*o’*) and 2×CH(*m‴*)), 129.3 (2×CH(*m*)), 129.9 (CH(5)), 130.1 (CH(2″)), 135.3 (C(*i*)), 136.4 (C(*i‴*)), 138.1 (C(6)), 139.8 (C(*i’*)), 141.0 (C(*i″*)), 144.2 (C(*p*)), 166.4 and 167.1 (2 COO) ppm. HRMS (ESI) *m/z*: calcd for C_37_H_36_NO_6_S^+^ [M+H]^+^ 622.2258; found: 622.2259.

*Dimethyl trans,trans-2-(3-(4-tolyl)-4-phenyl-6-((E)-styryl)-1-tosyl-1,2,3,4-tetrahydropyridin-2-yl)malonate* (**3c**)**.** Yellowish solid (119 mg, 75%). R_f_ = 0.36 (petroleum ether–EtOAc 3:1). M.p. 42–44 °C. ^1^H NMR (300.1 MHz, CDCl_3_): δ 2.17 (s, 3H, C(*p’*)-Me), 2.49 (s, 3H, C(*p*)-Me), 2.51 (dd, ^3^*J*_3,4_ = 11.1 Hz, ^3^*J*_4,5_ = 3.9 Hz, 1H, H(4)), 3.19 and 3.54 (both s, 2×3H, 2 CO_2_Me), 3.59 (dd, ^3^*J*_3,4_ = 11.1 Hz, ^3^*J*_2,3_ = 8.3 Hz, 1H, H(3)), 4.02 (d, ^3^*J*_2,2′_ = 6.2 Hz, 1H, H(2′)), 5.10 (dd, ^3^*J*_2,3_ = 8.3 Hz, ^3^*J*_2,2′_ = 6.3 Hz, 1H, H(2)), 6.17 (d, ^3^*J*_4,5_ = 3.9 Hz, 1H, H(5)), 6.63–6.73 (m, 4H, 2×H(*o’*) and 2×H(*o″*)), 6.76 (two doublets, ^3^*J*_1″,2″_ = 16.3 Hz, 2H, H(1″) and H(2″)), 6.81–6.89 (m, 2H, 2×H(*m’*)), 6.99–7.09 (m, 3H, 2×H(*m″*) and H(*p″*)), 7.19–7.25 (m, 1H, H(*p‴*)), 7.28–7.35 (m, 2H, 2×H(*m‴*)), 7.35–7.44 (m, 4H, 2×H(*m*) and 2×H(*o‴*)), 7.89 (d, *J* = 8.2 Hz, 2H, 2×H(*o*)) ppm. ^13^C NMR (75.5 MHz, CDCl_3_): δ 20.9 (C(*p’*)-Me), 21.6 (C(*p*)-Me), 46.8 (CH(4)), 52.0 and 52.3 (2 CO_2_Me), 55.1 (CH(3)), 56.3 (CH(2′)), 62.3 (CH(2)), 125.9 (CH(1″)), 126.4 (CH(*p″*)), 126.7 (2×CH(*o‴*)), 127.8 (CH(*p‴*)), 128.1 and 128.5 (2×CH(*o’*) and 2×CH(*o″*)), 128.1 (2×CH(*m″*)), 128.3 (2×CH(*o*)), 128.6 (2×CH(*m‴*)), 128.7 (2×CH(*m’*)), 129.5 (2×CH(*m*)), 129.8 (CH(5)), 130.4 (CH(2″)), 135.7 (C(*p*)), 136.2 (C(*p’*)), 136.8 and 137.0 (C(*i’*) and C(*i‴*)), 138.3 (C(6)), 141.5 (C(*i″*)), 144.3 (C(*i*)), 166.8 and 167.5 (2 COO) ppm. HRMS (ESI) *m/z*: calcd for C_38_H_37_NNaO_6_S^+^ [M+Na]^+^ 658.2234; found: 658.2228. 

*Dimethyl trans,trans-2-(3-(4-methoxyphenyl)-4-phenyl-6-((E)-styryl)-1-tosyl-1,2,3,4-tetrahydropyridin-2-yl)malonate* (**3d**)**.** Beige solid (132 mg, 81%). R_f_ = 0.35 (petroleum ether–EtOAc 3:1). M.p. 65–67 °C. ^1^H NMR (300.1 MHz, CDCl_3_): δ 2.49 (s, 3H, C(*p*)-Me), 2.49 (dd, ^3^*J*_3,4_ = 11.2 Hz, ^3^*J*_4,5_ = 3.8 Hz, 1H, H(4)), 3.22 and 3.54 (both s, 2×3H, 2 CO_2_Me), 3.57 (dd, ^3^*J*_3,4_ = 11.2 Hz, ^3^*J*_2,3_ = 8.0 Hz, 1H, H(3)), 3.68 (s, 3H, OMe), 4.02 (d, ^3^*J*_2,2′_ = 6.3 Hz, 1H, H(2′)), 5.09 (dd, ^3^*J*_2,3_ = 8.0 Hz, ^3^*J*_2,2′_ = 6.3 Hz, 1H, H(2)), 6.18 (d, ^3^*J*_4,5_ = 3.8 Hz, 1H, H(5)), 6.58 (d, *J* = 8.6 Hz, 2H, 2×H(*m’*)), 6.64–6.75 (m, 4H, 2×H(*o’*) and 2×H(*o″*)), 6.72 and 6.77 (two doublets, ^3^*J*_1″,2″_ = 16.0 Hz, 2H, H(1″) and H(2″)), 6.98–7.12 (m, 3H, 2×H(*m″*) and H(*p″*)), 7.21–7.43 (m, 7H, 2×H(*o‴*), 2×H(*m‴*), H(*p‴*) and 2×H(*m*)), 7.90 (d, *J* = 8.3 Hz, 2H, 2×H(*o*)) ppm. ^13^C NMR (75.5 MHz, CDCl_3_): δ 21.7 (C(*p*)-Me), 47.0 (CH(4)), 52.1 and 52.4 (2 CO_2_Me), 54.8 (CH(3)), 55.1 (OMe), 56.3 (CH(2′)), 62.3 (CH(2)), 113.4 (2×CH(*m’*)), 125.9 (CH(1″)), 126.4 (CH(*p″*)), 126.7 (2×CH(*o‴*)), 127.8 (CH(*p‴*)), 128.1 (2×CH(*o″*)), 128.2 (2×CH(*m″*)), 128.3 (2×CH(*o*)), 128.6 (2×CH(*m‴*)), 130.0 (CH(5)), 129.5 (2×CH(*m*)), 129.6 (2×CH(*o’*)), 130.4 (CH(2″)), 132.1 (C(*i’*), 135.7 (C(*p*)), 136.8 (C(*i‴*)), 138.3 (C(6)), 141.5 (C(*i″*)), 144.4 (C(*i*)), 158.2 (C(*p’*)), 166.8 and 167.5 (2 COO) ppm. HRMS (ESI) *m/z*: calcd for C_38_H_38_NO_7_S^+^ [M+H]^+^ 652.2363; found: 652.2350. 

*Dimethyl trans,trans-2-(3-(4-fluorophenyl)-4-phenyl-6-((E)-styryl)-1-tosyl-1,2,3,4-tetrahydropyridin-2-yl)malonate* (**3e**)**.** Beige solid (113 mg, 71%). R_f_ = 0.55 (petroleum ether–EtOAc 3:1). M.p. 58–60 °C. ^1^H NMR (300.1 MHz, CDCl_3_): δ 2.48 (s, 3H, C(*p*)-Me), 2.59 (dd, ^3^*J*_3,4_ = 11.1 Hz, ^3^*J*_4,5_ = 3.9 Hz, 1H, H(4)), 3.23 and 3.52 (both s, 2×3H, 2 CO_2_Me), 3.61 (dd, ^3^*J*_3,4_ = 11.1 Hz, ^3^*J*_2,3_ = 7.9 Hz, 1H, H(3)), 4.00 (d, ^3^*J*_2,2′_ = 6.6 Hz, 1H, H(2′)), 5.13 (dd, ^3^*J*_2,3_ = 7.9 Hz, ^3^*J*_2,2′_ = 6.6 Hz, 1H, H(2)), 6.21 (d, ^3^*J*_4,5_ = 3.9 Hz, 1H, H(5)), 6.65–6.73 (m, 2H, 2×H(*o″*)), 6.73 and 6.76 (two doublets, ^3^*J*_1″,2″_ = 16.0 Hz, 2H, H(1″) and H(2″)), 6.70–6.81 (m, 2H, 2×H(*m’*)), 6.74–6.85 (m, 2H, 2×H(*o’*)), 7.01–7.11 (m, 3H, 2×H(*m″*) and H(*p″*)), 7.21–7.28 (m, 1H, H(*p‴*)), 7.28–7.42 (m, 6H, 2×H(*o‴*), 2×H(*m‴*) and 2×H(*m*)), 7.89 (d, *J* = 8.3 Hz, 2H, 2×H(*o*)) ppm. ^13^C NMR (75.5 MHz, CDCl_3_): δ 21.6 (C(*p*)-Me), 47.1 (CH(4)), 52.1 and 52.4 (2 CO_2_Me), 54.9 (CH(3)), 56.4 (CH(2′)), 62.1 (CH(2)), 114.9 (d, ^2^*J*_C,F_ = 21.3 Hz, 2×CH(*m’*)), 125.7 (CH(1″)), 126.6 (CH(*p″*)), 126.7 (2×CH(*o‴*)), 127.9 (CH(*p‴*)), 128.0 (2×CH(*o″*)), 128.29 and 128.32 (2×CH(*o*) and 2×CH(*m″*)), 128.6 (2×CH(*m‴*)), 129.8 (CH(5)), 129.6 (2×CH(*m*)), 130.1 (d, ^3^*J*_C,F_ = 7.9 Hz, 2×CH(*o’*)), 130.6 (CH(2″)), 135.8 (C(*p*)), 136.1 (d, ^4^*J*_C,F_ = 3.3 Hz, C(*i’*)), 136.6 (C(*i‴*)), 138.5 (C(6)), 141.1 (C(*i″*)), 144.5 (C(*i*)), 161.5 (d, ^1^*J*_C,F_ = 21.3 Hz, C(*p’*)), 166.7 and 167.4 (2 COO) ppm. ^19^F NMR (282.5 MHz, CDCl_3_): δ -115.4 (tt, ^3^*J*_H,F_ = 8.4 Hz, ^4^*J*_H,F_ = 5.2 Hz) ppm. HRMS (ESI) *m/z*: calcd for C_37_H_34_FNNaO_6_S^+^ [M+Na]^+^ 662.1983; found: 662.1973. 

*Dimethyl trans,trans-2-(3-(4-chlorophenyl)-4-phenyl-6-((E)-styryl)-1-tosyl-1,2,3,4-tetrahydropyridin-2-yl)malonate* (**3f**)**.** Yellowish solid (129 mg, 79%). R_f_ = 0.57 (petroleum ether–EtOAc 3:1). M.p. 82–83 °C. ^1^H NMR (300.1 MHz, CDCl_3_): δ 2.48 (s, 3H, C(*p*)-Me), 2.62 (dd, ^3^*J*_3,4_ = 10.8 Hz, ^3^*J*_4,5_ = 3.8 Hz, 1H, H(4)), 3.25 and 3.51 (both s, 2×3H, 2 CO_2_Me), 3.62 (dd, ^3^*J*_3,4_ = 10.8 Hz, ^3^*J*_2,3_ = 7.8 Hz, 1H, H(3)), 3.99 (d, ^3^*J*_2,2′_ = 6.7 Hz, 1H, H(2′)), 5.12 (dd, ^3^*J*_2,3_ = 7.8 Hz, ^3^*J*_2,2′_ = 6.7 Hz, 1H, H(2)), 6.19 (d, ^3^*J*_4,5_ = 3.9 Hz, 1H, H(5)), 6.67–6.75 (m, 2H, 2×H(*o″*)), 6.70 and 6.77 (two doublets, ^3^*J*_1″,2″_ = 16.0 Hz, 2H, H(1″) and H(2″)), 6.74–6.82 (m, 2H, 2×H(*o’*)), 6.99–7.14 (m, 5H, 2×H(*m’*), 2×H(*m″*) and H(*p″*)), 7.21–7.28 (m, 1H, H(*p‴*)), 7.28–7.42 (m, 6H, 2×H(*o‴*), 2×H(*m‴*) and 2×H(*m*)), 7.86 (d, *J* = 8.3 Hz, 2H, 2×H(*o*)) ppm. ^13^C NMR (75.5 MHz, CDCl_3_): δ 21.6 (C(*p*)-Me), 46.7 (CH(4)), 52.1 and 52.4 (2 CO_2_Me), 54.8 (CH(3)), 56.3 (CH(2′)), 61.9 (CH(2)), 125.6 (CH(1″)), 126.6 (CH(*p″*)), 126.7 (2×CH(*o‴*)), 127.9 (CH(*p‴*)), 127.9 (2×CH(*o″*)), 128.2 (2×CH(*o*)), 128.2 and 128.3 (2×CH(*m’*) and 2×CH(*m″*)), 128.6 (2×CH(*m‴*)), 129.2 (CH(5)), 129.5 (2×CH(*m*)), 129.9 (2×CH(*o’*)), 130.6 (CH(2″)), 132.5 (C(*p’*)), 135.8 (C(*p*)), 136.6 (C(*i‴*)), 138.5 (C(6)), 138.9 (C(*i’*), 141.0 (C(*i″*)), 144.5 (C(*i*)), 166.6 and 167.3 (2 COO) ppm. HRMS (ESI) *m/z*: calcd for C_37_H_35_ClNO_6_S^+^ [M+H]^+^ 656.1868; found: 656.1879. 

*Dimethyl trans,trans-2-(3-(3-bromophenyl)-4-phenyl-6-((E)-styryl)-1-tosyl-1,2,3,4-tetrahydropyridin-2-yl)malonate* (**3g**)**.** Yellowish solid (122 mg, 70%). R_f_ = 0.43 (petroleum ether–EtOAc 3:1). M.p. 45–46 °C. ^1^H NMR (300.1 MHz, CDCl_3_): δ 2.47 (dd, ^3^*J*_3,4_ = 11.2 Hz, ^3^*J*_4,5_ = 3.9 Hz, 1H, H(4)), 2.51 (s, 3H, C(*p*)-Me), 3.21 and 3.54 (both s, 2×3H, 2 CO_2_Me), 3.65 (dd, ^3^*J*_3,4_ = 11.1 Hz, ^3^*J*_2,3_ = 8.1 Hz, 1H, H(3)), 4.07 (d, ^3^*J*_2,2′_ = 6.2 Hz, 1H, H(2′)), 5.04 (dd, ^3^*J*_2,3_ = 8.1 Hz, ^3^*J*_2,2′_ = 6.3 Hz, 1H, H(2)), 6.18 (d, ^3^*J*_4,5_ = 3.9 Hz, 1H, H(5)), 6.64–6.69 (m, 2H, 2×H(*o’*)), 6.69 (ddd, ^3^*J*_6′″,5′″_ = 7.8, ^4^*J*_6′″,2′″_ = 1.8, ^4^*J*_6′″,4′″_ = 1.1 Hz, 1H, H(6′″)), 6.75 and 6.79 (two doublets, ^3^*J*_1″,2″_ = 16.0 Hz, 2H, H(1″) and H(2″)), 6.88 (t, ^3^*J*_5′″,4′″_ = ^3^*J*_5′″,6′″_ = 7.8 Hz, 1H, H(5′″)), 6.93 (t, ^4^*J*_2′″,4′″_ = ^4^*J*_2′″,6′″_ = 1.8, 1H, H(2′″)), 7.00–7.11 (m, 3H, 2×H(*m’*) and H(*p’*)), 7.14 (ddd, ^3^*J*_4′″,5′″_ = 7.8, ^4^*J*_4′″,2′″_ = 1.8, ^4^*J*_4′″,6′″_ = 1.1 Hz, 1H, H(4′″)), 7.20–7.28 (m, 1H, H(*p″*)), 7.28–7.36 (m, 2H, 2×H(*m″*)), 7.37–7.47 (m, 4H, 2×H(*o″*) and 2×H(*m*)), 7.91 (d, *J* = 8.3 Hz, 2H, 2×H(*o*)) ppm. ^13^C NMR (75.5 MHz, CDCl_3_): δ 21.7 (C(*p*)-Me), 46.8 (CH(4)), 52.1 and 52.5 (2 CO_2_Me), 55.0 (CH(3)), 56.3 (CH(2′)), 61.9 (CH(2)), 122.1 (C(3′″)), 125.8 (CH(1″)), 126.7 (CH(*p’*)), 126.8 (2×CH(*o’*)), 127.8 and 127.9 (CH(6′″) and CH(*p″*)), 128.0 (2×CH(*o″*)), 128.2 (2×CH(*o*)), 128.4 (2×CH(*m’*)), 128.6 (2×CH(*m″*)), 129.2 (CH(5)), 129.7 (CH(5′″)), 129.9 (2×CH(*m*)), 129.9 (CH(4′″)), 130.7 (CH(2″)), 131.0 (CH(2′″)), 135.6 (C(*p*)), 136.7 (C(*i″*)), 138.6 (C(6)), 140.9 (C(*i’*)), 142.8 (C(1′″)), 144.7 (C(*i*)), 166.6 and 167.4 (2 COO) ppm. HRMS (ESI) *m/z*: calcd for C_37_H_35_BrNO_6_S^+^ [M+H]^+^ 700.1363; found: 700.1355. 

*Dimethyl trans,trans-2-(3-(2-chlorophenyl)-4-phenyl-6-((E)-styryl)-1-tosyl-1,2,3,4-tetrahydropyridin-2-yl)malonate* (**3h**)**.** Beige solid (126 mg, 77%). R_f_ = 0.40 (petroleum ether–EtOAc 3:1). M.p. 87–88 °C. ^1^H NMR (300.1 MHz, CDCl_3_): δ 2.46 (s, 3H, C(*p*)-Me), 2.67 (br.s, 1H, H(4)), 3.37 (br.s, 3H, C(3′)O_2_Me), 3.61 (s, 3H, C(1′)O_2_Me), 3.94 (br.s, 1H, H(2′)), 4.23 (dd, ^3^*J*_3,4_ = 10.9 Hz, ^3^*J*_2,3_ = 8.5 Hz, 1H, H(3)), 5.15 (br.s, 1H, H(2)), 6.27 (d, ^3^*J*_4,5_ = 3.9 Hz, 1H, H(5)), 6.70–6.79 (m, 4H, 2×H(*o’*), H(1″) and H(2″)), 6.91–7.10 (m, 5H, H(*p’*), H(3″), H(4″) and 2×H(*m’*)), 7.12–7.43 (m, 8H, H(5″), H(*p″*), 2×H(*m″*), 2×H(*o″*) and 2×H(*m*)), 7.90 (d, *J* = 8.3 Hz, 2H, 2×H(*o*)) ppm. ^13^C NMR (75.5 MHz, CDCl_3_): δ 21.7 (C(*p*)-Me), 47.4 (br.s, CH(4)), 51.0 (br.s, CH(3)), 52.2 (C(3′)O_2_Me), 52.6 (C(1′)O_2_Me), 56.5 (CH(2′)), 62.5 (CH(2)), 125.7 (CH(1″)), 126.7 (CH(*p’*)), 126.8 (2×CH(*o″*)), 127.0 (br.s, CH(5′″)), 127.9 (CH(*p″*)), 128.0 (2×CH(*m’*), CH(4′″) and CH(6′″)), 128.3 (2×CH(*o’*)), 128.5 (2×CH(*o*)), 128.6 (2×CH(*m″*)), 129.3 (br.s, CH(3′″)), 129.6 (2×CH(*m*)), 129.9 (CH(5)), 130.7 (CH(2″)), 134.5 (br.s, C(2′″)), 135.9 (C(*p*)), 136.7 (C(*i″*)), 138.4 (br.s, C(1′″)), 138.8 (br.s, C(6)), 140.1 (br.s, C(*i’*)), 144.5 (C(*i*)), 166.7 and 167.2 (2 COO) ppm. HRMS (ESI) *m/z*: calcd for C_37_H_35_ClNO_6_S^+^ [M+H]^+^ 656.1868; found: 656.1853. 

*Dimethyl trans,trans-2-(3-(naphthalen-1-yl)-4-phenyl-6-((E)-styryl)-1-tosyl-1,2,3,4-tetrahydropyridin-2-yl)malonate* (**3i**)**.** Yellowish solid (90 mg, 54%). R_f_ = 0.52 (petroleum ether–EtOAc 3:1). M.p. 51–53 °C. ^1^H NMR (300.1 MHz, CDCl_3_): δ 2.48 (s, 3H, C(*p*)-Me), 2.88 and 3.42 (both s, 2×3H, 2 CO_2_Me), 2.91 (dd, ^3^*J*_3,4_ = 9.7 Hz, ^3^*J*_4,5_ = 3.9 Hz, 1H, H(4)), 4.06 (d, ^3^*J*_2,2′_ = 6.5 Hz, 1H, H(2′)), 4.78 (dd, ^3^*J*_3,4_ = 9.7 Hz, ^3^*J*_2,3_ = 7.5 Hz, 1H, H(3)), 5.31 (dd, ^3^*J*_2,3_ = 7.5 Hz, ^3^*J*_2,2′_ = 6.5 Hz, 1H, H(2)), 6.29 (d, ^3^*J*_4,5_ = 3.9 Hz, 1H, H(5)), 6.74–6.80 (m, 2H, 2×H(*o’*)), 6.84 and 6.88 (two doublets, ^3^*J*_1″,2″_ = 16.0 Hz, 2H, H(1″) and H(2″)), 6.83–6.94 (m, 3H, H(*p’*) and 2×H(*m’*)), 7.20–7.39 (m, 9H, 2×H(*m*), 2×H(*m″*), H(*p″*), H(2′″), H(3′″), H(6′″), H(7′″)), 7.40–7.50 (m, 2H, 2×H(*o″*)), 7.57 (d, ^3^*J*_4′″,3′″_ = 7.6 Hz, 1H, H(4′″)), 7.60–7.66 (m, 1H, H(5′″)), 7.80 (d, *J* = 8.3 Hz, 2H, 2×H(o)), 7.89–7.97 (m, 1H, H(8′″)) ppm. ^13^C NMR (75.5 MHz, CDCl_3_): δ 21.7 (C(*p*)-Me), 47.3 (CH(3) and CH(4)), 51.9 and 52.4 (2 CO_2_Me), 56.0 (CH(2′)), 63.3 (CH(2)), 123.3 (CH(8′″)), 125.1 and 125.2 (CH(2′″) and CH(3′″)), 125.3 (CH(6′″)), 125.8 (CH(7′″)), 126.0 (CH(1″)), 126.3 (CH(*p’*)), 126.8 (2×CH(*o″*)), 127.4 (CH(4′″)), 127.5 (CH(5)), 127.9 (CH(*p″*)), 127.9 (2×CH(*m’*)), 128.0 (2×CH(*o’*)), 128.2 (CH(5′″)), 128.3 (2×CH(*o*)), 128.7 (2×CH(*m″*)), 129.6 (2×CH(*m*)), 130.6 (CH(2″)), 132.0 (C(8′″a)), 133.3 (C(4′″a)), 136.1 (C(*p*)), 136.8 (C(*i″*)), 137.8 (C(1′″)), 138.5 (C(6)), 141.3 (C(*i’*)), 144.3 (C(*i*)), 166.6 and 167.9 (2 COO) ppm. HRMS (ESI) *m/z*: calcd for C_41_H_38_NO_6_S^+^ [M+H]^+^ 672.2414; found: 672.2399. 

*Dimethyl trans,trans-2-(3-(4-nitrophenyl)-4-phenyl-6-((E)-styryl)-1-tosyl-1,2,3,4-tetrahydropyridin-2-yl)malonate* (**3j**)**.** Orange solid (93 mg, 56%). R_f_ = 0.36 (petroleum ether–EtOAc 3:1). M.p. 39–40 °C. ^1^H NMR (300.1 MHz, CDCl_3_): δ 2.48 (s, 3H, C(*p*)-Me), 2.84 (dd, ^3^*J*_3,4_ = 10.7 Hz, ^3^*J*_4,5_ = 3.8 Hz, 1H, H(4)), 3.28 and 3.50 (both s, 2×3H, 2 CO_2_Me), 3.72 (dd, ^3^*J*_3,4_ = 10.7 Hz, ^3^*J*_2,3_ = 7.3 Hz, 1H, H(3)), 3.96 (d, ^3^*J*_2,2′_ = 7.1 Hz, 1H, H(2′)), 5.25 (dd, ^3^*J*_2,2′_ = 7.1 Hz, ^3^*J*_2,3_ = 7.3 Hz, 1H, H(2)), 6.25 (d, ^3^*J*_4,5_ = 3.9 Hz, 1H, H(5)), 6.64 (d, ^3^*J*_1″,2″_ = 16.0 Hz, 1H, H(2″)), 6.70–6.79 (m, 2H, 2×H(*o″*)), 6.76 (d, ^3^*J*_1″,2″_ = 16.0 Hz, 1H, H(1″)), 7.05–7.14 (m, 3H, 2×H(*m″*) and H(*p″*)), 7.07 (d, *J* = 8.7 Hz, 2H, 2×H(*o’*)), 7.23–7.29 (m, 1H, H(*p‴*)), 7.30–7.36 (m, 4H, 2×H(*o‴*), 2×H(*m‴*)), 7.37 (d, *J* = 8.2 Hz, 2H, 2×H(*m*)), 7.87 (d, *J* = 8.2 Hz, 2H, 2×H(*o*)), 7.94 (d, *J* = 8.7 Hz, 2H, 2×H(*m’*)) ppm. ^13^C NMR (75.5 MHz, CDCl_3_): δ 21.6 (C(*p*)-Me), 46.9 (CH(4)), 52.2 and 52.5 (2 CO_2_Me), 55.7 (CH(3)), 56.5 (CH(2′)), 61.5 (CH(2)), 123.2 (2×CH(*m’*)), 125.2 (CH(1″)), 126.7 (2×CH(*o‴*)), 127.0 (CH(*p″*)), 127.8 (2×CH(*o″*)), 128.1 (CH(*p‴*)), 128.3 (2×CH(*o*)), 128.5 and 128.6 (2×CH(*m″*) and 2×CH(*m‴*)), 128.9 (CH(5)), 129.4 (2×CH(*o’*)), 129.6 (2×CH(*m*)), 131.0 (CH(2″)), 136.0 (C(*p*)), 136.3 (C(*i‴*)), 138.8 (C(6)), 140.5 (C(*i″*)), 144.7 (C(*i*)), 146.6 (C(*p’*)), 148.4 (C(*i’*)), 166.5 and 167.1 (2 COO) ppm. HRMS (ESI) *m/z*: calcd for C_37_H_34_N_2_NaO_8_S^+^ [M+Na]^+^ 689.1928; found: 689.1918. 

*Dimethyl trans,trans-2-(3-(4-chlorophenyl)-4-(4-nitrophenyl)-6-((E)-4-methoxystyryl)-1-tosyl-1,2,3,4-tetrahydropyridin-2-yl)malonate* (**3k**) and *dimethyl trans,trans-2-(3-(4-chlorophenyl)-4-(4-methoxyphenyl)-6-((E)-4-nitrostyryl)-1-tosyl-1,2,3,4-tetrahydropyridin-2-yl)malonate* (**3k’**). Orange thick oil (131 mg, 72%, rr 1/1 at rt, 12 h; 111 mg, 61%, rr 1/1 at -20 °C, 5 d). R_f_ = 0.34 (petroleum ether–EtOAc 3:1). HRMS (ESI) *m/z*: calcd for C_38_H_36_ClN_2_O_9_S^+^ [M+H]^+^ 731.1825; found: 731.1819. **3k**: ^1^H NMR (300.1 MHz, CDCl_3_): δ 2.50 (s, 3H, C(*p*)-Me), 2.87 (dd, ^3^*J*_3,4_ = 11.2 Hz, ^3^*J*_4,5_ = 3.9 Hz, 1H, H(4)), 3.23 and 3.51 (both s, 2×3H, 2 CO_2_Me), 3.67 (dd, ^3^*J*_3,4_ = 11.2 Hz, ^3^*J*_2,3_ = 7.7 Hz, 1H, H(3)), 3.83 (s, 3H, OMe), 3.94 (d, ^3^*J*_2,2′_ = 6.6 Hz, 1H, H(2′)), 5.17 (dd, ^3^*J*_2,3_ = 7.7 Hz, ^3^*J*_2,2′_ = 6.6 Hz, 1H, H(2)), 6.07 (d, ^3^*J*_4,5_ = 3.9 Hz, 1H, H(5)), 6.59 (two doublets, ^3^*J*_1″,2″_ = 16.0 Hz, 2H, H(1″) and H(2″)), 6.80 (d, *J* = 8.6 Hz, 2H, 2×H(*o’*)), 6.86 (d, *J* = 8.9 Hz, 2H, 2×H(*m‴*)), 6.91 (d, *J* = 8.8 Hz, 2H, 2×H(*o″*)), 7.07 (d, *J* = 8.6 Hz, 2H, 2×H(*m’*)), 7.28 (d, *J* = 8.9 Hz, 2H, 2×H(*o‴*)), 7.40 (d, *J* = 8.3 Hz, 2H, 2×H(*m*)), 7.89 (d, *J* = 8.3 Hz, 2H, 2×H(*o*)), 7.96 (d, *J* = 8.8 Hz, 2H, 2×H(*m″*)) ppm. ^13^C NMR (75.5 MHz, CDCl_3_): δ 21.68 (C(*p*)-Me), 47.1 (CH(4)), 52.19 and 52.53 (2 CO_2_Me), 55.0 (CH(3)), 55.3 (OMe), 56.3 (CH(2′)), 61.9 (CH(2)), 114.2 (2×CH(*m‴*)), 122.9 (CH(1″)), 123.6 (2×CH(*m″*)), 126.5 (CH(5)), 128.08 (2×CH(*o‴*)), 128.4 and 128.6 (2×CH(*o*) and 2×CH(*m’*)), 129.0 (2×CH(*o″*)), 129.6 and 129.7 (2×CH(*o’*) and 2×CH(*m*)), 131.0 (CH(2″)), 132.7 (C(*i‴*)), 133.1 (C(*p’*)), 135.9 (C(*p*)), 138.2 (C(*i’*)), 139.6 (C(6)), 144.7 (C(*i*)), 146.8 (C(*p″*)), 148.9 (C(*i″*)), 159.8 (C(*p‴*)), 166.52 and 167.3 (2 COO) ppm. **3k’**: ^1^H NMR (300.1 MHz, CDCl_3_): δ 2.40 (dd, ^3^*J*_3,4_ = 11.4 Hz, ^3^*J*_4,5_ = 3.9 Hz, 1H, H(4)), 2.52 (s, 3H, C(*p*)-Me), 3.22 and 3.56 (both s, 2×3H, 2 CO_2_Me), 3.61 (dd, ^3^*J*_3,4_ = 11.4 Hz, ^3^*J*_2,3_ = 8.4 Hz, 1H, H(3)), 3.69 (s, 3H, OMe), 4.03 (d, ^3^*J*_2,2′_ = 5.9 Hz, 1H, H(2′)), 4.98 (dd, ^3^*J*_2,3_ = 8.4 Hz, ^3^*J*_2,2′_ = 5.9 Hz, 1H, H(2)), 6.27 (d, ^3^*J*_4,5_ = 3.9 Hz, 1H, H(5)), 6.54 (d, *J* = 8.9 Hz, 2H, 2×H(*o″*)), 6.61 (d, *J* = 8.9 Hz, 2H, 2×H(*m″*)), 6.69 (d, *J* = 8.3 Hz, 2H, 2×H(*o’*)), 6.84 and 6.93 (two doublets, ^3^*J*_1″,2″_ = 16.0 Hz, 2H, H(1″) and H(2″)), 7.04 (d, *J* = 8.3 Hz, 2H, 2×H(*m’*)), 7.43 (d, *J* = 8.3 Hz, 2H, 2×H(*m*)), 7.54 (d, *J* = 8.8 Hz, 2H, 2×H(*o‴*)), 7.88 (d, *J* = 8.3 Hz, 2H, 2×H(*o*)), 8.20 (d, *J* = 8.8 Hz, 2H, (2×H(*m‴*))) ppm. ^13^C NMR (75.5 MHz, CDCl_3_): δ 21.70 (C(*p*)-Me), 46.1 (CH(4)), 52.16 and 52.46 (2 CO_2_Me), 54.7 (CH(3)), 55.2 (OMe), 56.0 (CH(2′)), 62.0 (CH(2)), 113.8 (2×CH(*m″*)), 124.1 (2×CH(*m‴*)), 127.1 (2×CH(*o‴*)), 128.1 (CH(1″)), 128.2 (2×CH(*o*)), 128.3 (2×CH(*m’*)), 128.8 (2×CH(*o″*)), 129.8 (2×CH(*m*)), 129.9 (2×CH(*o’*)), 130.36 (CH(2″)), 132.5 (C(*i″*)), 132.5 (C(*p’*), 132.8 (CH(5)), 135.3 (C(*p*)), 138.0 (C(6)), 138.6 (C(*i’*), 143.3 (C(*i‴*)), 144.9 (C(*i*)), 147.0 (C(*p‴*)), 158.3 (C(*p″*)), 166.53 and 167.5 (2 COO) ppm. 

*Dimethyl trans,trans-2-(3-(4-chlorophenyl)-4-phenyl-6-((E)-2,6-dichlorostyryl)-1-tosyl-1,2,3,4-tetrahydropyridin-2-yl)malonate* (**3l**) and *dimethyl trans,trans-2-(3-(4-chlorophenyl)-4-(2,6-dichlorophenyl)-6-((E)-styryl)-1-tosyl-1,2,3,4-tetrahydropyridin-2-yl)malonate* (**3l’**). Yellowish thick oil (94 mg, 52%, rr 1/1 at rt, 12 h; 101 mg, 56%, rr 1.2/1 at -20 °C, 5 d). R_f_ = 0.53 (petroleum ether–EtOAc 3:1). HRMS (ESI) *m/z*: calcd for C_37_H_33_Cl_3_NO_6_S^+^ [M+H]^+^ 724.1089; found: 724.1086. **3l**: ^1^H NMR (300.1 MHz, CDCl_3_): δ 2.48 (s, 3H, C(*p*)-Me), 2.58 (dd, ^3^*J*_3,4_ = 10.3 Hz, ^3^*J*_4,5_ = 3.8 Hz, 1H, H(4)), 3.30 and 3.58 (both s, 2×3H, 2 CO_2_Me), 3.64 (dd, ^3^*J*_3,4_ = 10.3 Hz, ^3^*J*_2,3_ = 7.9 Hz, 1H, H(3)), 3.98 (d, ^3^*J*_2,2′_ = 6.6 Hz, 1H, H(2′)), 5.07 (dd, ^3^*J*_2,3_ = 7.9 Hz, ^3^*J*_2,2′_ = 6.6 Hz, 1H, H(2)), 6.24 (d, ^3^*J*_4,5_ = 3.8 Hz, 1H, H(5)), 6.68–6.75 (m, 2H, 2×H(*o″*)), 6.77 (d, *J* = 8.5 Hz, 2H, 2×H(*o’*)), 6.92 and 7.00 (two doublets, ^3^*J*_1″,2″_ = 16.0 Hz, 2H, H(1″) and H(2″)), 7.02 (d, *J* = 8.5 Hz, 2H, 2×H(*m’*)), 7.05–7.12 (m, 4H, 2×H(*m″*), H(*p″*) and H(*p‴*)), 7.34 (d, *J* = 8.1 Hz, 2H, 2×H(*m‴*)), 7.37 (d, *J* = 8.3 Hz, 2H, 2×H(*m*)), 7.88 (d, *J* = 8.3 Hz, 2H, 2×H(*o*)) ppm. ^13^C NMR (75.5 MHz, CDCl_3_): δ 21.7 (C(*p*)-Me), 46.4 (CH(4)), 52.2 and 52.4 (2 CO_2_Me), 54.6 (CH(3)), 56.1 (CH(2′)), 61.8 (CH(2)), 124.1 (CH(1″)), 126.7 (CH(*p″*)), 127.9 (2×CH(*o″*)), 128.1 (2×CH(*o*)), 128.2 (CH(*p‴*)), 128.3 and 128.4 (2×CH(*m’*) and 2×CH(*m″*)), 128.7 (2×CH(*m‴*)), 129.1 (CH(5)), 129.6 (2×CH(*m*)), 129.8 (2×CH(*o’*)), 132.7 (C(*p’*)), 133.7 (C(*i‴*)), 134.4 (CH(2″)), 134.7 (2×CCl(*o‴*)), 135.5 (C(*p*)), 138.3 (C(6)), 138.9 (C(*i’*), 141.0 (C(*i″*)), 144.4 (C(*i*)), 166.7 and 167.4 (2 COO) ppm. **3l’**: ^1^H NMR (300.1 MHz, CDCl_3_): δ 2.44 (s, 3H, C(*p*)-Me), 3.21 and 3.60 (both s, 2×3H, 2 CO_2_Me), 3.73 (dd, ^3^*J*_3,4_ = 11.6 Hz, ^3^*J*_4,5_ = 4.1 Hz, 1H, H(4)), 4.17 (d, ^3^*J*_2,2′_ = 6.3 Hz, 1H, H(2′)), 4.56 (dd, ^3^*J*_3,4_ = 11.6 Hz, ^3^*J*_2,3_ = 8.7 Hz, 1H, H(3)), 5.15 (dd, ^3^*J*_2,3_ = 8.7 Hz, ^3^*J*_2,2′_ = 6.3 Hz, 1H, H(2)), 6.24 (d, ^3^*J*_4,5_ = 4.1 Hz, 1H, H(5)), 6.73 (two doublets, ^3^*J*_1″,2″_ = 16.0 Hz, 2H, H(1″) and H(2″)), 6.90–6.95 (m, 2H, 2×H(*m″*)), 6.93–7.00 (m, 2H, 2×H(*o’*)), 7.02–7.08 (m, 2H, 2×H(*m’*)), 7.14–7.20 (m, 1H, H(*p″*)), 7.24–7.43 (m, 5H, 2×H(*o‴*), 2×H(*m‴*) and H(*p‴*)), 7.40 (d, *J* = 8.3 Hz, 2H, 2×H(*m*)), 7.93 (d, *J* = 8.3 Hz, 2H, 2×H(*o*)) ppm. ^13^C NMR (75.5 MHz, CDCl_3_): δ 21.6 (C(*p*)-Me), 42.9 (CH(4)), 49.8 (CH(3)), 52.0 and 52.5 (2 CO_2_Me), 56.7 (CH(2′)), 62.5 (CH(2)), 125.8 (CH(1″)), 126.8 (2×CH(*o‴*)), 127.3 (CH(5)), 127.8 (CH(*p‴*)), 128.05 (2×CH(*o*)), 128.05 (2×CH(*m’*)), 128.14 and 128.7 (2×CH(*m″*)), 128.6 (2×CH(*m‴*)), 129.90 (2×CH(*m*)), 129.92 (CH(*p″*)), 129.95 (2×CH(*o’*)), 130.1 (CH(2″)), 133.0 (C(*p’*)), 134.55 and 134.68 (2×CCl(*o″*)), 135.6 (C(*p*)), 136.0 (C(*i″*)), 136.7 (C(*i‴*)), 137.7 (C(6)), 137.8 (C(*i’*), 144.5 (C(*i*)), 166.7 and 167.2 (2 COO) ppm. 

*Dimethyl trans,trans-2-(3-(2-chlorophenyl)-4-phenyl-6-((E)-2,6-dichlorostyryl)-1-tosyl-1,2,3,4-tetrahydropyridin-2-yl)malonate* (**3m**) and *dimethyl trans,trans-2-(3-(2-chlorophenyl)-4-(2,6-dichlorophenyl)-6-((E)-styryl)-1-tosyl-1,2,3,4-tetrahydropyridin-2-yl)malonate* (**3m’**). Yellowish thick oil (134 mg, 74%, rr 2/1 at rt; 98 mg, rr 4/1 54% at -20 °C). R_f_ = 0.33 (petroleum ether–EtOAc 3:1). HRMS (ESI) *m/z*: calcd for C_37_H_33_Cl_3_NO_6_S^+^ [M+H]^+^ 724.1089; found: 724.1085. Major isomer (**3m**): ^1^H NMR (300.1 MHz, CDCl_3_): δ 2.48 (s, 3H, C(*p*)-Me), 2.50 (br.s, 1H, H(4)), 3.44 (br.s, 3H, C(3′)O_2_Me), 3.69 (s, 3H, C(1′)O_2_Me), 3.89 (br.s, 1H, H(2′)), 4.25 (br.s, 1H, H(3)), 5.02 (br.s, 1H, H(2)), 6.28 (d, ^3^*J*_4,5_ = 3.8 Hz, 1H, H(5)), 6.68–6.77 (m, 2H, 2×H(*o’*)), 6.99 (two doublets, ^3^*J*_1″,2″_ = 16.0 Hz, 2H, H(1″) and H(2″)), 6.92–7.36 (m, 10H, 2×H(*m’*), H(*p’*), 2×H(*m″*), H(*p″*), H(3′″), H(4′″), H(5′″), H(6′″)), 7.40 (d, *J* = 8.3 Hz, 2H, 2×H(*m*)), 7.95 (d, *J* = 8.3 Hz, 2H, 2×H(*o*)) ppm. ^13^C NMR (75.5 MHz, CDCl_3_): δ 21.7 (C(*p*)-Me), 47.1 (br.s, CH(4)), 52.3 (C(3′)O_2_Me), 52.6 (C(1′)O_2_Me), 56.1 (CH(3)), 56.1 (CH(2′)), 62.6 (CH(2)), 124.4 (CH(1″)), 126.7 (CH(*p’*)), 124.36 (CH(1″)), 126.7 (C(*p’*)), 127.0 and 127.1 (CH(4′″) and CH(5′″)), 128.0 (2×CH(*m’*)), 128.1 (CH(*p″*)), 128.20 (2×CH(*o’*)), 128.37 (2×CH(*o*)), 128.71 (2×CH(*m″*)), 128.82 (CH(6′″)), 129.42 (CH(3′″)), 129.55 (2×CH(*m*)), 130.5 (CH(5)), 133.9 (C(*i″*)), 134.2 (CH(2″)), 134.6 (C(2′″)–Cl) , 134.7 (C(*o″*)–Cl), 135.3 (C(*p*)), 138.7 (C(6) and C(1′*″*)), 140.1 (C(*i’*)), 144.5 (C(*i*)), 166.7 (C(3′)O_2_Me) and 167.3 (br.s, C(1′)O_2_Me) ppm. Minor isomer (**3m’**): ^1^H NMR (300.1 MHz, CDCl_3_): δ 2.39 (s, 3H, C(*p*)-Me), 3.57 and 3.61 (both s, 2×3H, 2 CO_2_Me), 3.96 (d, ^3^*J*_2,2′_ = 8.2 Hz, 1H, H(2′)), 4.04 (dd, ^3^*J*_3,4_ = 11.4 Hz, ^3^*J*_4,5_ = 3.8 Hz, 1H, H(4)), 4.63 (dd, ^3^*J*_3,4_ = 11.4 Hz, ^3^*J*_2,3_ = 6.2 Hz, 1H, H(3)), 5.38 (dd, ^3^*J*_2,3_ = 8.2 Hz, ^3^*J*_2,2′_ = 6.2 Hz, 1H, H(2)), 6.37 (d, ^3^*J*_4,5_ = 3.8 Hz, 1H, H(5)), 6.59 and 6.67 (two doublets, ^3^*J*_1″,2″_ = 16.0 Hz, 2H, H(1″) and H(2″)), 6.92–7.36 (m, 14H, 2×H(*m’*), H(*p’*), 2×H(*o″*), 2×H(*m″*), H(*p″*), H(3′″), H(4′″), H(5′″), H(6′″) and 2×H(*m*)), 7.92 (m, 2H, 2×H(*o*)) ppm. ^13^C NMR (75.5 MHz, CDCl_3_): δ 21.6 (C(*p*)-Me), 43.3 (CH(4)), 48.6 (CH(3)), 52.5 and 52.7 (2 CO_2_Me), 58.1 (CH(2′)), 63.3 (CH(2)), 125.5 (CH(1″)), 126.7 (2×CH(*o″*)), 127.8 (CH(5′″)), 127.9 (CH(*p″*)), 128.1 (CH(5)), 128.16 (CH(*p’*)), 128.17 (CH(4′″)), 128.44 (2×CH(*o*)), 128.56 (2×CH(*m’*)), 128.78 (2×CH(*m″*)), 129.42 (CH(3′″)), 129.6 (2×CH(*m*)), 130.3 (CH(2″)), 130.5 (CH(6′″)), 134.1 (C(2′″)–Cl), 134.2 (C(*i’*)), 136.3 (C(*p*)), 136.7 (2×CCl(*o’*) and C(*i″*)), 137.5 (C(6)), 138.5 (C(1′″)), 144.4 (C(*i*)), 166.6 and 166.8 (2 COO) ppm.

#### 3.2.3. General Synthetic Procedure and Spectroscopic Data for Heterocycles **5**

To a solution of vinyltetrahydropyridine **3** (0.046 mmol) in CH_2_Cl_2_ (1 mL) PTAD (8 mg, 0.046 mmol, 1.0 equiv) was added. After 15 min, the final solution was removed under vacuum to afford corresponding heterocycles **5a,b**.

*Dimethyl 2-((5SR,8RS,9SR,10SR,10aSR)-7-((4-nitrophenyl)sulfonyl)-1,3-dioxo-2,5,9,10-tetraphenyl-2,3,5,7,8,9,10,10a-octahydro-1H-pyrido[3,2-c]*[1,2,4]*triazolo[1,2-a]pyridazin-8-yl)malonate* (**5a**). Yellow solid (38 mg, 99%). R_f_ = 0.34 (petroleum ether–EtOAc 3:1). M.p. >250 °C. ^1^H NMR (300.1 MHz, CDCl_3_): δ 3.66 and 3.67 (both s, 2×3H, 2 CO_2_Me), 3.68 (dd, ^3^*J*_10,10a_ = 11.3 Hz, ^3^*J*_9,10_ = 9.1 Hz, 1H, H(10)), 3.91 (dd, ^3^*J*_9,10_ = 9.1 Hz, ^3^*J*_8,9_ = 5.7 Hz, H(9)), 4.42 (d, ^3^*J*_8,2′_ = 9.1 Hz, 1H, H(2′)), 5.12 (dd, ^3^*J*_8,2′_ = 9.1 Hz, ^3^*J*_8,9_ = 5.7 Hz, H(8)), 5.31 (t, ^3^*J*_5,6_ = ^5^*J*_5,10a_ = 2.1 Hz, H(5)), 5.38 (ddd, ^3^*J*_10,10a_ = 11.3 Hz, ^5^*J*_5,10a_ = 2.1 Hz, ^4^*J*_6,10a_ = 1.2 Hz, 1H, H(10a)), 5.80 (dd, ^3^*J*_5,6_ = 2.1 Hz, ^4^*J*_6,10a_ = 1.2 Hz, 1H, H(6)), 7.02–7.13 (m, 7H, 2×H(*o_A_*), 2×H(*o_D_*), 2×H(*m_D_*) and H(*p_D_*)), 7.14–7.22 (m, 5H, 2×H(*o_E_*), 2×H(*m_E_*) and H(*p_E_*)), 7.22–7.35 (m, 3H, 2×H(*m_A_*) and H(*p_A_*)), 7.41–7.49 (m, 5H, 2×H(*o_B_*), 2×H(*m_B_*) and H(*p_B_*)), 7.72 (d, *J* = 9.0 Hz, 2H, 2×H(*o_C_*)), 8.04 (d, *J* = 9.0 Hz, 2H, 2×H(*m_C_*)) ppm. ^13^C NMR (75.5 MHz, CDCl_3_): δ 51.2 (CH(9)), 53.14 and 53.17 (2 CO_2_Me), 53.9 (CH(10)), 55.0 (CH(10a)), 57.0 (CH(2′)), 61.9 (CH(5)), 66.2 (CH(8)), 116.6 (CH(6)), 124.1 (2×CH(*m_C_*)), 125.3 (2×CH(*o_A_*)), 127.1 (2×CH(*o_B_*)), 127.6 (CH(*p_D_*)), 128.1 (CH(*p_E_*)), 128.2 (CH(*p_A_*)), 128.60 and 128.62 (2×CH(*o_E_*) and 2×CH(*m_E_*)), 128.7 and 128.80 (2×CH(*o_D_*) and 2×CH(*m_D_*)), 128.84 (2×CH(*m_A_*)), 128.9 (CH(*p_B_*)), 129.1 (2×CH(*m_B_*)), 129.2 (2×CH(*o_C_*)), 130.8 (C(*i_A_*)), 134.1 (C(6a)), 137.3 (C(*i_E_*)), 138.1 (C(*i_B_*)), 138.9 (C(*i_D_*)), 143.9 (C(*i_C_*)), 148.5 and 155.2 (C(1) and C(3)), 150.3 (C(*p_C_*)), 166.6 and 167.5 (2 COO) ppm. HRMS (ESI) *m/z*: calcd for C_44_H_38_N_5_O_10_S^+^ [M+H]^+^ 828.2334; found: 828.2321.

*Dimethyl 2-((5SR,8RS,9SR,10SR,10aSR)-1,3-dioxo-2,5,9,10-tetraphenyl-7-tosyl-2,3,5,7,8,9,10,10a-octahydro-1H-pyrido[3,2-c]*[1,2,4]*triazolo[1,2-a]pyridazin-8-yl)malonate* (**5b**). Yellowish solid (37 mg, 99%). R_f_ = 0.35 (petroleum ether–EtOAc 3:1). M.p. 181–183 °C. ^1^H NMR (300.1 MHz, CDCl_3_): δ 2.45 (s, 3H, C(*p’*)-Me), 3.58 and 3.59 (both s, 2×3H, 2 CO_2_Me), 3.63 (dd, ^3^*J*_10,10a_ = 11.2, ^3^*J*_9,10_ = 9.8 Hz, 1H, H(10)), `4.08 (dd, ^3^*J*_9,10_ = 9.8 Hz, ^3^*J*_8,9_ = 7.2 Hz, 1H, H(9)), 4.51 (d, ^3^*J*_8,2′_ = 8.9 Hz, 1H, H(2′)), 5.03 (dd, ^3^*J*_8,2′_ = 8.9 Hz, ^3^*J*_8,9_ = 7.2 Hz, 1H, H(8)), 5.33 (m, 1H, H(5)), 5.40 (d, ^3^*J*_10,10a_ = 11.2 Hz, 1H, H(10a)), 5.89 (m, 1H, H(6)), 7.01–7.15 (m, 7H, 2×H(*o_A_*), 2×H(*o_D_*), 2×H(*m_D_*) and H(*p_D_*)), 7.15–7.25 (m, 7H, 2×H(*m_C_*), 2×H(*o_E_*), 2×H(*m_E_*) and H(*p_E_*)), 7.25–7.37 (m, 3H, 2×H(*m_A_*) and H(*p_A_*)), 7.45–7.55 (m, 5H, 2×H(*o_B_*), 2×H(*m_B_*) and H(*p_B_*)), 7.61 (d, *J* = 8.2 Hz, 2H, 2×H(*o_C_*)) ppm. ^13^C NMR (75.5 MHz, CDCl_3_): δ 21.8 (C(*p’*)-Me), 50.9 (CH(9)), 53.13 and 53.15 (2 CO_2_Me), 55.3 (CH(10a)), 55.6 (CH(10)), 56.5 (CH(2′)), 61.9 (CH(5)), 66.2 (CH(8)), 117.8 (CH(6)), 125.4 (2×CH(*o_A_*)), 127.3 (CH(*p_D_*)), 127.4 (2×CH(*o_B_*)), 128.0 (CH(*p_E_*)), 128.07 (2×CH(*o_C_*)), 128.11 (CH(*p_A_*)), 128.4, 128.6, 128.83, 128.85 and 128.9 (2×CH(*o_D_*), 2×CH(*m_D_*), 2×CH(*o_E_*), 2×CH(*m_E_*) and CH(*p_B_*)), 128.9 (2×CH(*m_A_*)), 129.2 (2×CH(*m_B_*)), 129.8 (2×CH(*m_C_*)), 130.7 (C(*i_A_*)), 135.1 (C(6a)), 135.6 (C(*p_C_*)), 137.4 (C(*i_E_*)), 138.1 (C(*i_B_*)), 138.7 (C(*i_D_*)), 144.8 (C(*i_C_*)), 148.5 and 155.3 (C(1) and C(3)), 167.1 and 167.7 (2 COO) ppm. ^15^N NMR (30.4 MHz, CDCl_3_; reconstructed from ^1^H–^15^N HMBC): δ 120.3 (s, N(7)), 126.5 (s, N(4)), 128.9 (s, N(11)), 147.9 (s, N(2)) ppm. HRMS (ESI) *m/z*: calcd for C_45_H_41_N_4_O_8_S^+^ [M+H]^+^ 797.2640; found: 797.2640.

#### 3.2.4. General Synthetic Procedure and Spectroscopic Data for Heterocycle **6**

To a solution of heterocycle **5a** (0.046 mmol, 38 mg) in CH_2_Cl_2_ (1 mL) at −20 °C SiO_2_ (50 mg) was added. After 12 h, the final solution was evaporated and purified using column chromatography on silica gel (petroleum ether–EtOAc 10:1 to 1:1) to afford title compound **6**.

*Dimethyl 2-((5SR,8RS,9SR,10SR)-7-((4-nitrophenyl)sulfonyl)-1,3-dioxo-2,5,9,10-tetraphenyl-2,3,5,6,7,8,9,10-octahydro-1H-pyrido[3,2-c]*[1,2,4]*triazolo[1,2-a]pyridazin-8-yl)malonate* (**6**). Yellow solid (10 mg, 26%). R_f_ = 0.69 (petroleum ether–EtOAc 1:1). M.p. >250 °C. ^1^H NMR (300.1 MHz, CDCl_3_): δ 3.38 and 3.78 (both s, 2×3H, 2 CO_2_Me), 3.46 (ddd, ^2^*J*_6′,6″_ = 17.9 Hz, ^3^*J*_5,6′_ = 2.0 Hz, *J* = 1.8 Hz, 1H, H(6′)), 3.70 (ddd, ^2^*J*_6′,6″_ = 17.9 Hz, ^3^*J*_5,6″_ = 7.4 Hz, *J* = 2.0 1H, H(6″)), 4.08 (br.s, 1H, H(9)), 4.15 (d, ^3^*J*_8,2′_ = 11.4 Hz, 1H, H(2′)), 5.38 (br.s, 1H, H(10)), 5.55 (dd, ^3^*J*_8,2′_ = 11.4 Hz, ^3^*J*_8,9_ = 1.7 Hz, 1H, H(8)), 5.65 (dd, ^3^*J*_5,6″_ = 7.4 Hz, ^3^*J*_5,6′_ = 2.0 Hz, 1H, H(5)), 6.88–6.96 (m, 2H, 2×CH(*o_A_*)), 7.00 (d, *J* = 8.8 Hz, 2H, 2×H(*o_C_*)), 7.23–7.51 (m, 18H, Ar), 7.85 (d, *J* = 8.8 Hz, 2H, 2×H(*m_C_*)) ppm. ^13^C NMR (75.5 MHz, CDCl_3_): δ 32.6 (CH_2_(6)), 38.2 (CH(10)), 45.9 (CH(9)), 52.6 and 53.5 (2 CO_2_Me), 53.3 (CH(2′)), 56.2 (CH(5)), 61.2 (CH(8)), 119.9 (C(10a)), 120.1 (C(6a)), 124.2 (2×CH(*m_C_*)), 125.9 (2×CH(*o_A_*)), 127.1 (2×CH(*o_B_*) and CH(*p_D_*)), 128.0 (2×CH(*o_D_*) and CH(*p_A_*)), 128.7 (2×CH(*o_C_*)), 129.0 (2×CH(*m_A_*) and 2×CH(*o_E_*)), 129.2 (2×CH(*m_B_*), 2×CH(*m_D_*) and 2×CH(*m_E_*)), 129.20 (CH(*p_B_*)), 129.6 (CH(*p_E_*)), 130.0 (C(*i_A_*)), 136.5 (C(*i_B_*)), 141.8 (C(*i_D_*)), 142.3 (C(*i_E_*)), 145.1 (C(*i_C_*)), 146.5 and 152.3 (C(1) and C(3)), 149.3 (C(*p_C_*)), 167.1 and 167.3 (2 COO) ppm. ^15^N NMR (30.4 MHz, CDCl_3_; reconstructed from ^1^H–^15^N HMBC): δ 116.9 (s, N(7)), 132 and 132.5 (two singlets, N(4) and N(11)), 150.2 (s, N(2)), 365.5 (s, NO_2_) ppm. HRMS (ESI) *m/z*: calcd for C_44_H_38_N_5_O_10_S^+^ [M+H]^+^ 828.2334; found: 828.2317.

#### 3.2.5. General Synthetic Procedure and Spectroscopic Data for Azadiene **4**

To a solution of **3b** (80 mg, 12.9 µmol) in THF (5 mL) at −78 °C was added dropwise (during 15–20 min) a freshly prepared ~0.35 M solution of sodium naphthalenide in THF (2.5 mL, 6 equiv) and the reaction mixture stirred 15 min. The reaction was then quenched by the addition of saturated ammonium chloride (2 mL) and warmed to room temperature. The whole was poured onto saturated ammonium chloride (20 mL) and extracted with ethyl acetate (3×20 mL). The combined organic extracts were dried over Na_2_SO_4_, filtered, and concentrated in vacuo. The residual naphthalene was then removed by filtration through a short pad of deactivated silica gel using petroleum ether—EtOAc (1:1) as the eluent. The resulting solution was evaporated, and the residue was purified using column chromatography on deactivated silica gel (benzene–EtOAc 50:1 to 10:1) to afford the desired azadiene **4** as a beige solid. A stock solution of sodium naphthalenide in THF was prepared by dissolving naphthalene (980 mg, 7.65 mmol) in freshly dried THF (20 mL) under an argon atmosphere, to which sodium metal (160 mg, 6.96 mmol) was added in small pieces with vigorous stirring for 2 h until the mixture had formed a homogeneous dark green solution.

*Dimethyl trans,trans-2-(3,4-diphenyl-6-((E)-styryl)-2,3,4,5-tetrahydropyridin-2-yl)malonate* (**4**)**.** Beige solid (24 mg, 40%). R_f_ = 0.31 (petroleum ether–EtOAc 3:1). M.p. 62–63 °C. ^1^H NMR (300.1 MHz, CDCl_3_): δ 2.70 (m, 1H, H(5′)), 3.07 (m, 1H, H(5″)), 3.23 (m, 2H, H(3) and H(4)), 3.60 (m, 1H, H(2′)), 3.63 (two s, 2×3H, 2 CO_2_Me), 4.71 (m, 1H, H(2)), 6.90 (d, ^3^*J*_1″,2″_ = 16.3 Hz, 1H, H(1″)), 6.97 (d, ^3^*J*_1″,2″_ = 16.3 Hz, 1H, H(2″)), 6.96–7.21 (m, 10H, 2×H(*o*), 2×H(*m*), H(*p*), 2×H(*o’*), 2×H(*m’*) and H(*p’*)), 7.21–7.40 (m, 3H, 2×H(*m″*) and H(*p″*)), 7.42–7.50 (m, 2H, 2×H(*o″*)) ppm. ^13^C NMR (75.5 MHz, CDCl_3_): δ 35.2 (CH_2_(5)), 44.5 (CH(4)), 48.7 (CH(3)), 52.1 and 52.3 (2 CO_2_Me), 55.2 (CH(2′)), 66.2 (CH(2)), 126.4 and 126.7 (CH(*p*) and CH(*p’*)), 127.2 (2×CH(*o″*)), 127.6 and 128.3 (2×CH(*o*), 2×CH(*o’*), 2×CH(*m*) and 2×CH(*m’*)), 128.4 (CH(*p″*)), 128.7 (CH(*m″*)), 131.0 (CH(1″)), 135.0 (CH(2″)), 136.1 (C(*i″*)), 140.1 and 142.8 (C(*i*) and C(*i’*)), 166.3 (C(6)), 168.4 and 169.1 (2 COO) ppm. ^1^H NMR (300.1 MHz, CD_2_Cl_2_): δ 2.70 (ddd, ^2^*J*_5′,5″_ = 17.9 Hz, ^3^*J*_4,5′_ = 11.6 Hz, ^5^*J*_2,5′_ = 3.4 Hz, 1H, H(5′)), 3.11 (ddd, ^2^*J*_5′,5″_ = 17.9 Hz, ^3^*J*_4,5″_ = 5.5 Hz, ^5^*J*_2,5″_ = 1.6 Hz, 1H, H(5″)), 3.20 (dd, ^3^*J*_3,4_ = 11.6 Hz, ^3^*J*_2,3_ = 10.3 Hz, 1H, H(3)), 3.35 (td, ^3^*J*_3,4_ = ^3^*J*_4,5′_ = 11.6 Hz, ^3^*J*_4,5″_ = 5.5 Hz, 1H, H(4)), 3.54 (d, ^3^*J*_2,2′_ = 4.7 Hz, H(2′)), 3.63 and 3.72 (two s, 2×3H, 2 CO_2_Me), 4.61–4.73 (m, 1H, H(2)), 6.91 (d, ^3^*J*_1″,2″_ = 16.8 Hz, 1H, H(1″)), 7.08 (d, ^3^*J*_1″,2″_ = 16.8 Hz, 1H, H(2″)), 7.08–7.26 (m, 10H, 2×H(*o*), 2×H(*m*), H(*p*), 2×H(*o’*), 2×H(*m’*) and H(*p’*)), 7.32–7.45 (m, 3H, 2×H(*m″*) and H(*p″*)), 7.51–7.59 (m, 2H, 2×H(*o″*)) ppm. ^13^C NMR (75.5 MHz, CD_2_Cl_2_): δ 35.7 (CH_2_(5)), 44.3 (CH(4)), 48.7 (CH(3)), 51.9 and 52.2 (2 CO_2_Me), 55.3 (CH(2′)), 66.5 (CH(2)), 126.4 and 126.7 (CH(*p*) and CH(*p’*)), 127.2 (2×CH(*o″*)), 127.8 and 128.3 (2×CH(*o*), 2×CH(*o’*), 2×CH(*m*) and 2×CH(*m’*)), 128.5 (CH(*p″*)), 128.7 (CH(*m″*)), 130.6 (CH(1″)), 135.1 (CH(2″)), 136.1 (C(*i″*)), 140.4 and 143.2 (C(*i*) and C(*i’*)), 165.9 (C(6)), 168.3 and 169.0 (2 COO) ppm. HRMS (ESI) *m/z*: calcd for C_30_H_30_NO_4_^+^ [M+H]^+^ 468.2169; found: 468.2164.

#### 3.2.6. Gram-Scale Synthetic Procedure for Vinyltetrahydropyridine **3b**

A solution of styrlmalonate **1a** (10.7 mmol, 1.3 eq., 2.5 g), azatriene **2b** (8.9 mmol, 3.5 g), and Sn(OTf)_2_ (40 mol. %, 1.5 g) in dry CH_2_Cl_2_ (100 mL) was stirred 24 h at room temperature. Then the reaction mixture was filtrated through a short pad of silica gel, evaporated, and purified by column chromatography on silica gel (petroleum ether–EtOAc 15:1 to 3:1) to afford title compound **3b**; yield 4.1 g (74%).

## 4. Conclusions

In conclusion, the Lewis acid-catalyzed reactions of formal (4+2)-cycloaddition between styrylmalonates and N-sulfonyl-protected cross-conjugated azatrienes have been studied. The reaction occurs in good yields and makes it possible to obtain the corresponding vinyltetrahydropyridines as single *trans,trans*-diastereomers. The regioselectivity of the reaction was studied in the case of unsymmetrically substituted azatrienes, where the maximum ratio of regioisomers reached 4/1 at best. The resulting vinyltetrahydropyridines were tested as dienes in the Diels–Alder reaction with PTAD, which is an implementation of the DTHDA concept. Finally, the optimal detosylation conditions were found to obtain a new type of highly functionalized azadienes from the vinyltetrahydropyridines discussed above. In the future, we plan to study related new precursors in order to develop an approach to the diastereoselective synthesis of polycyclic saturated nitrogen-containing heterocycles, in particular, quinolizidines and azaphenalenes.

## Figures and Tables

**Table 1:** The reaction of unsymmetrically substituted azatrienes **2c,d** with styrylmalonates **1d,g**.

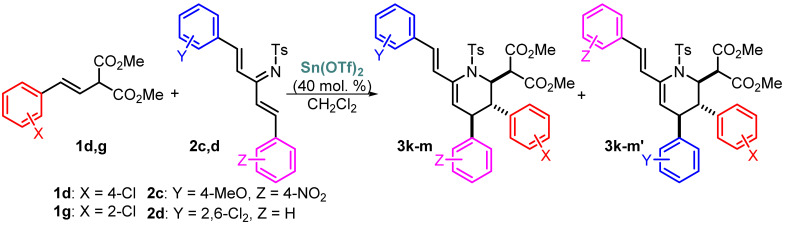								
Entry *	X	Y	Z	T, °C	t, Days	Products,	Total Yields, % **	Ratio 3/3′ **
1	4-Cl	4-MeO	4-NO_2_	20	0.5	**3k, 3k′**	72	1/1
2 ***	4-Cl	4-MeO	4-NO_2_	–20	3	**3k, 3k′**	61	1/1
3	4-Cl	2,6-Cl_2_	H	20	0.5	**3l, 3l′**	52	1/1
4	4-Cl	2,6-Cl_2_	H	–20	5	**3l, 3l′**	56	1.2/1
5	2-Cl	2,6-Cl_2_	H	20	0.5	**3m, 3m′**	74	2/1
6	2-Cl	2,6-Cl_2_	H	**–20**	5	**3m, 3m′**	54	**4/1**

* General conditions: **1**:**2** = 1.3:1 (mol.). ** Determined from ^1^H NMR spectra. *** Incomplete conversion (64%).

**Table 2:** The optimization of conditions for obtaining the product **6**.

Entry	Oxide	T, °C	t, h	Yield 6, %
1	SiO_2_	20	12	16
2	SiO_2_	–20	12	26
3 *	Al_2_O_3_	20	72	11 **
4	Al_2_O_3_	40	6	c.m. ***

* Incomplete conversion (30%). ** Determined by 1H NMR spectra. *** Complex mixture.

**Table 3:** The optimization of reaction conditions for detosylation of vinyltetrahydropyridine **3b**.

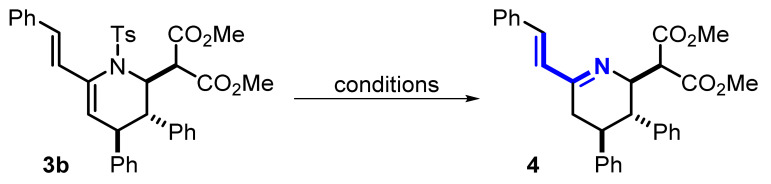					
No	Reagent	Solvent	T, °C	t, h	Yield 4, %
1	SmI_2_ (6 eq)	THF	20 → 65	6	n.r.
2	SmI_2_ (6 eq), HMPA (18 eq)	THF	20	0.25	traces
3 *	SmI_2_ (6 eq), HMPA (18 eq)	THF	–78	1	traces
4	SmI_2_ (10 eq), Et_3_N (20 eq), H_2_O (30 eq)	THF	20	0.25	n.r.
5	Mg (40 eq), )))	MeOH	20	1	further reduction (traces)
6	Mg (10 eq), )))	MeOH	20	0.25	
7	Mg (3 eq), HgCl_2_ (cat.)	MeOH	–20	1	n.r.
8	TfOH (1 eq)	DCE **	60	1	c.m.
9 *	TfOH (1 eq)	CH_2_Cl_2_	–20 → 20	1	c.m.
10	HBr/AcOH		100	1	c.m.
11	Na-naphthalenide (10 eq)	THF	–78	0.75	30
12	Na-naphthalenide (6 eq)	THF	–78	0.25	40
13	Na-naphthalenide(6 eq)	DME	–60	0.25	29

* Incomplete conversion (<10% traces of **3b** were found). ** 1,2-dichloroethane.

## Data Availability

All data obtained are contained in this article.

## Supplementary Materials

The following supporting information can be downloaded at: https://www.mdpi.com/article/10.3390/molecules28010088/s1, copies of NMR spectra.

## Sample Availability

Samples of the compounds are not available from the authors.
