# Supplementary material for: Lewis Acid-Catalyzed Formal (4+2)-Cycloaddition between Cross-Conjugated Azatrienes and Styrylmalonates: The Way to Functionalized Quinolizidine Precursors
Source: Molecules. 2022 Dec 22;28(1):88. doi: 10.3390/molecules28010088 (PMC9822494; doi:10.3390/molecules28010088)
Supplement: Supplementary file 1 [file molecules-28-00088-s001.zip › molecules-2058042-supplementary.pdf]

Lewis Acid-Catalyzed Formal (4+2)-Cycloaddition between Cross-Conjugated Azatrienes and Styrylmalonates: The Way to Functionalized Quinolizidine Precursors

Pavel G. Sergeev [1], Roman A. Novikov [1,*] and Yury V. Tomilov [1,]**

N. D. Zelinsky Institute of Organic Chemistry, Russian Academy of Sciences, 47 Leninsky prosp., 119991 Moscow, Russian Federation

* Correspondence: novikovfff@bk.ru; phone: +7(919)727-5362

** Correspondence: tom@ioc.ac.ru; phone, fax: +7(495)135-6390

SUPPORTING INFORMATION

Table of contents

Entry	Description	Page
1	Summary of discussed reactions	S2
2	NMR spectra of new compounds	S3

1. Summary of discussed reactions

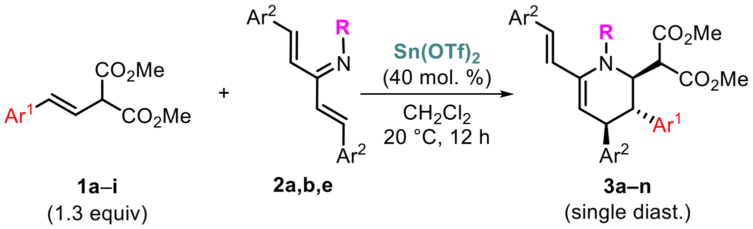

(from **2a**): Ar^2 = Ph; R = Ns; Ar^1 = Ph (**3a**, 76%)
(from **2b**): Ar^2 = Ph; R = Ts; Ar^1 = Ph (**3b**, 79%), 4-MeC_6H_4 (**3c**, 75%), 4-MeOC_6H_4 (**3d**, 81%), 4-FC_6H_4 (**3e**, 71%), 4-ClC_6H_4 (**3f**, 79%), 3-BrC_6H_4 (**3g**, 70%), 2-ClC_6H_4 (**3h**, 77%), 1-naphthyl (**3i**, 54%), 4-$NO_2C_6H_4$ (**3j**, 56%)
(from **2e**): Ar^2 = 4-MeOC_6H_4; R = Ts; Ar^1 = Ph (**3n**, traces)

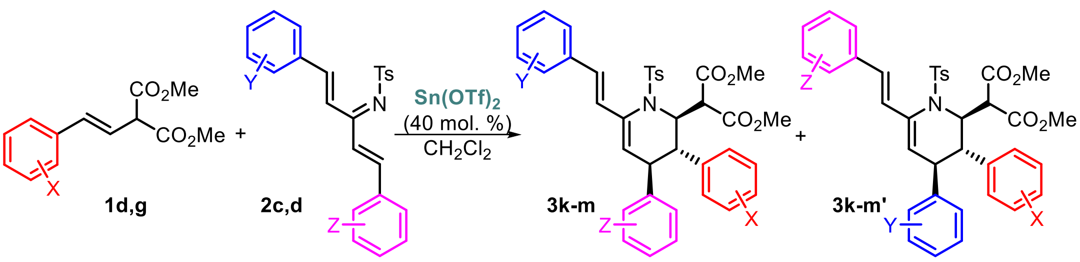

(from **2c**): Y = 4-MeO; Z = 4-NO_2; X = 4-Cl (**3k+3k'**)
(from **2d**): Y = 2,6-Cl_2; Z = H; X = 4-Cl (**3l+3l'**), 2-Cl (**3m+3m'**)

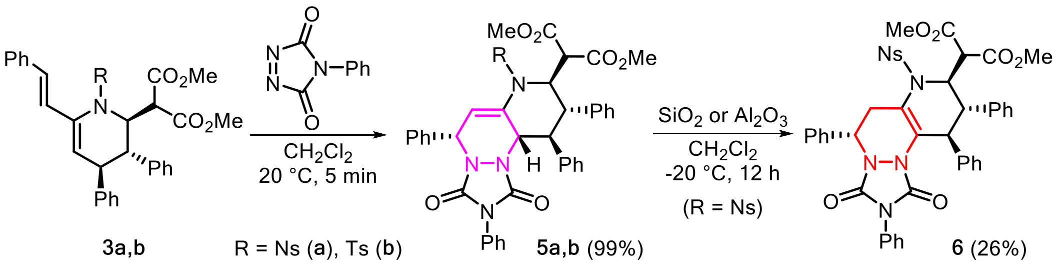

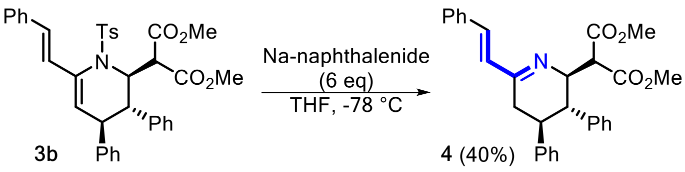

2. NMR spectra of new compounds

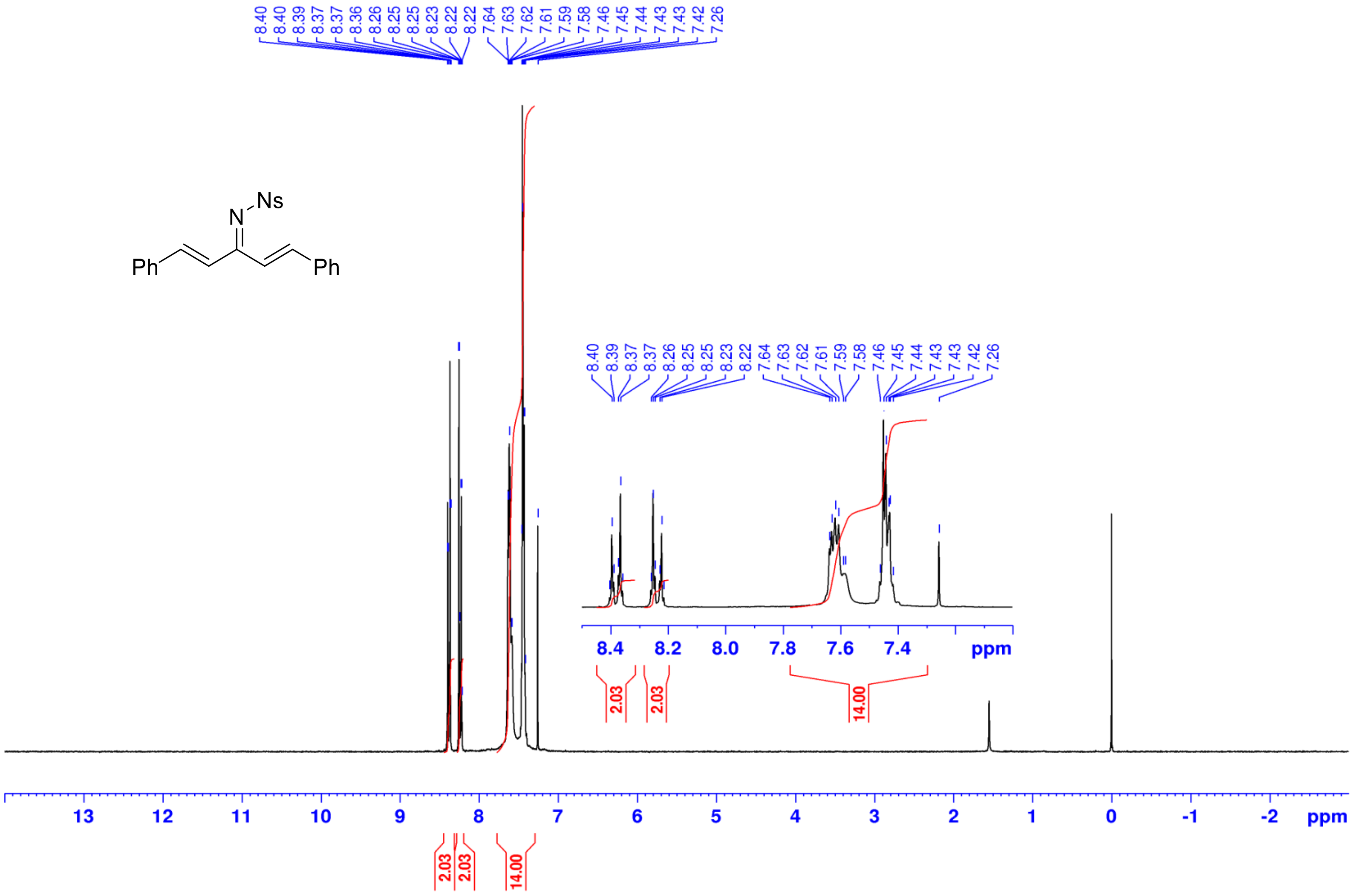

^{1}H NMR spectrum of **2a** (300.1 MHz, $CDCl_3$)

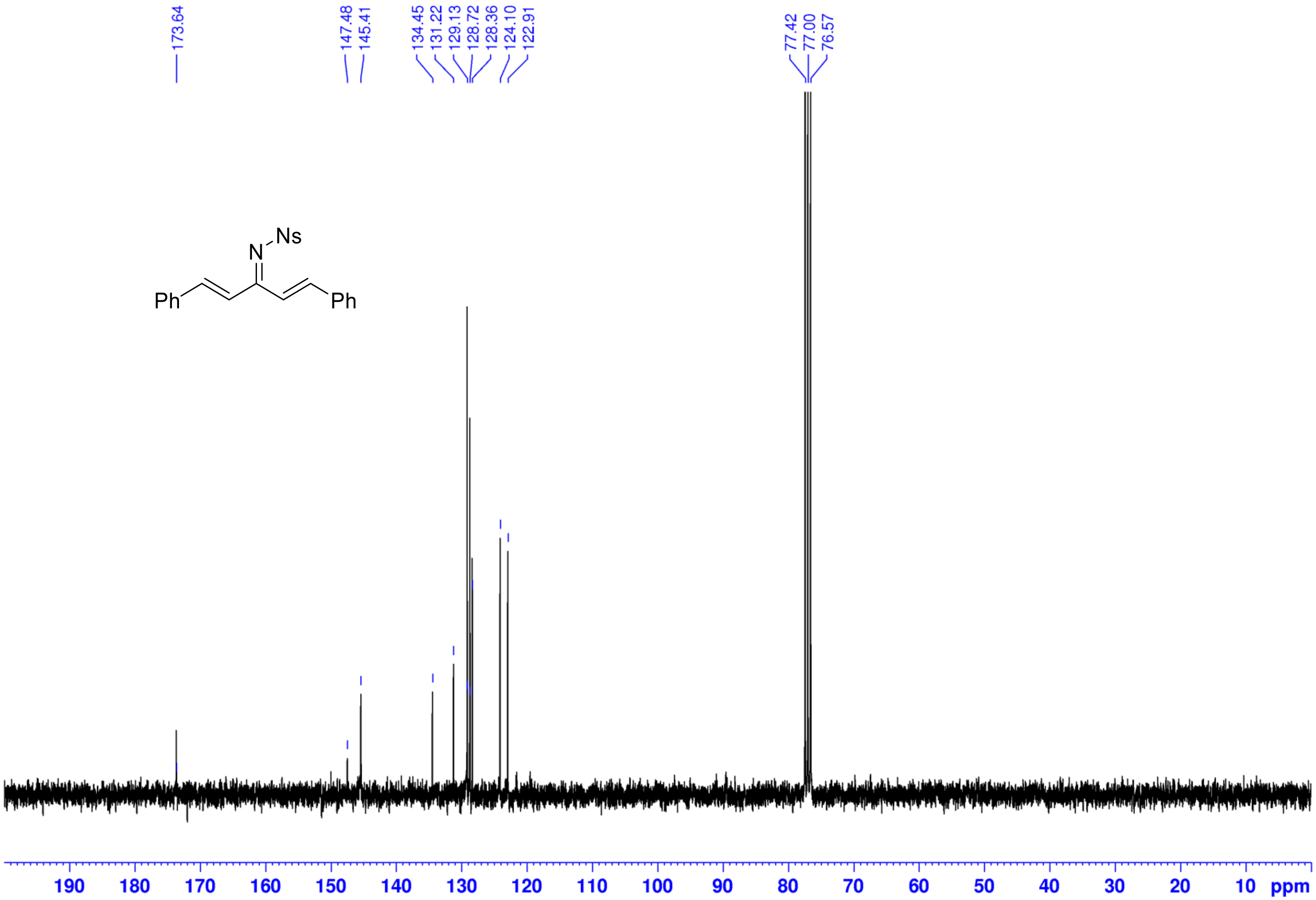

^{13}C NMR spectrum of **2a** (75.5 MHz, $CDCl_3$)

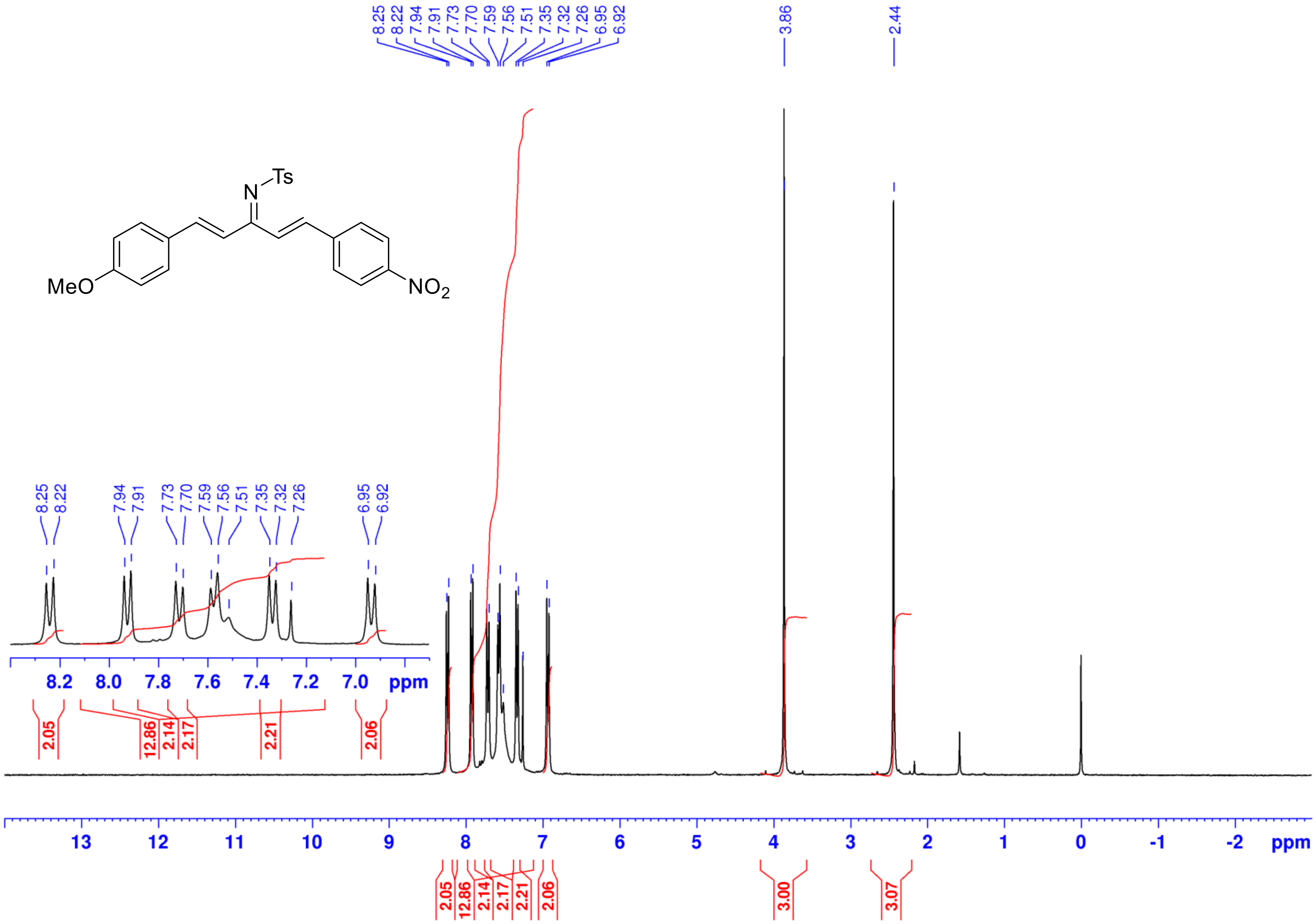

^{1}H NMR spectrum of **2c** (300.1 MHz, $CDCl_3$)

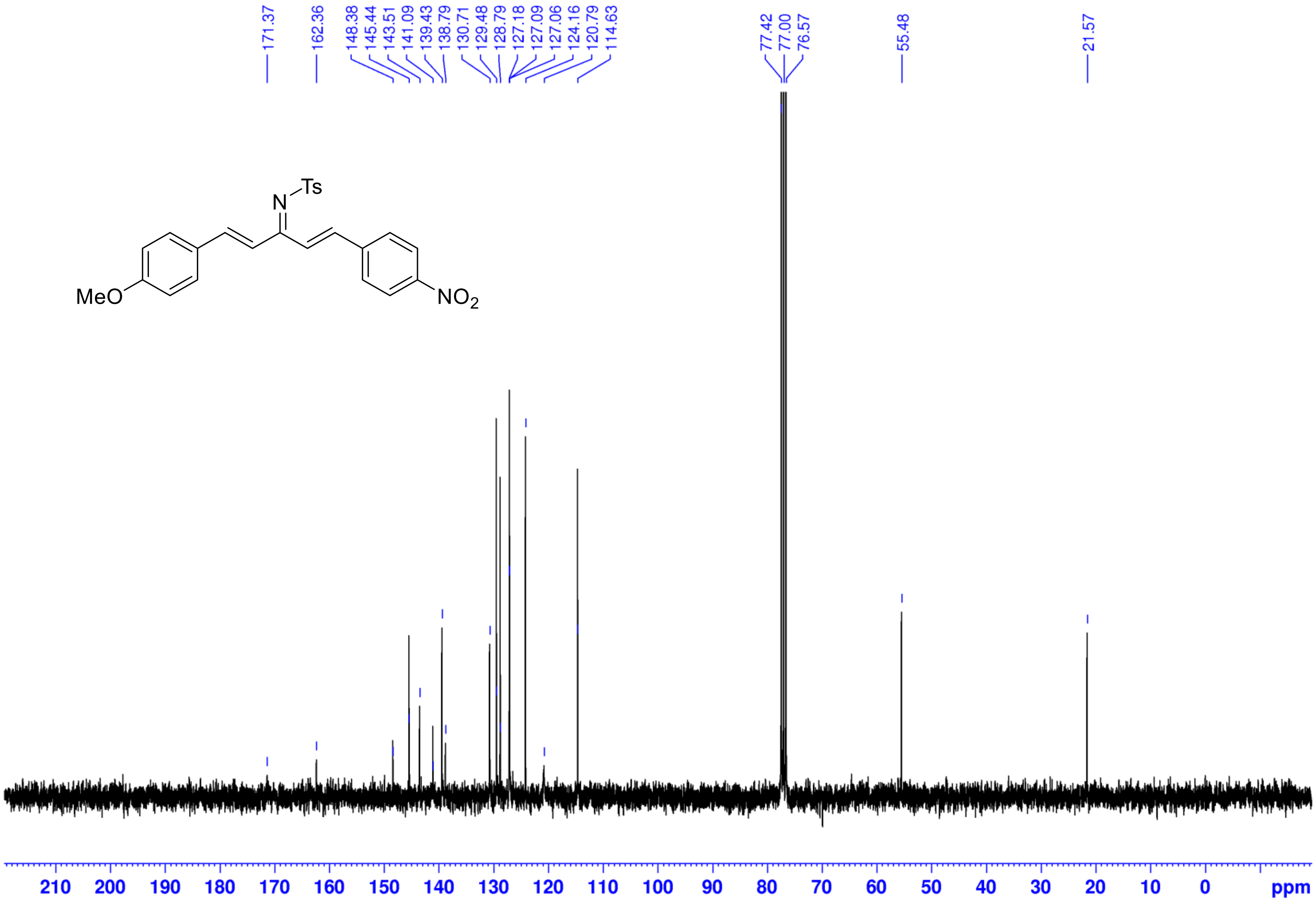

^{13}C NMR spectrum of **2c** (75.5 MHz, $CDCl_3$)

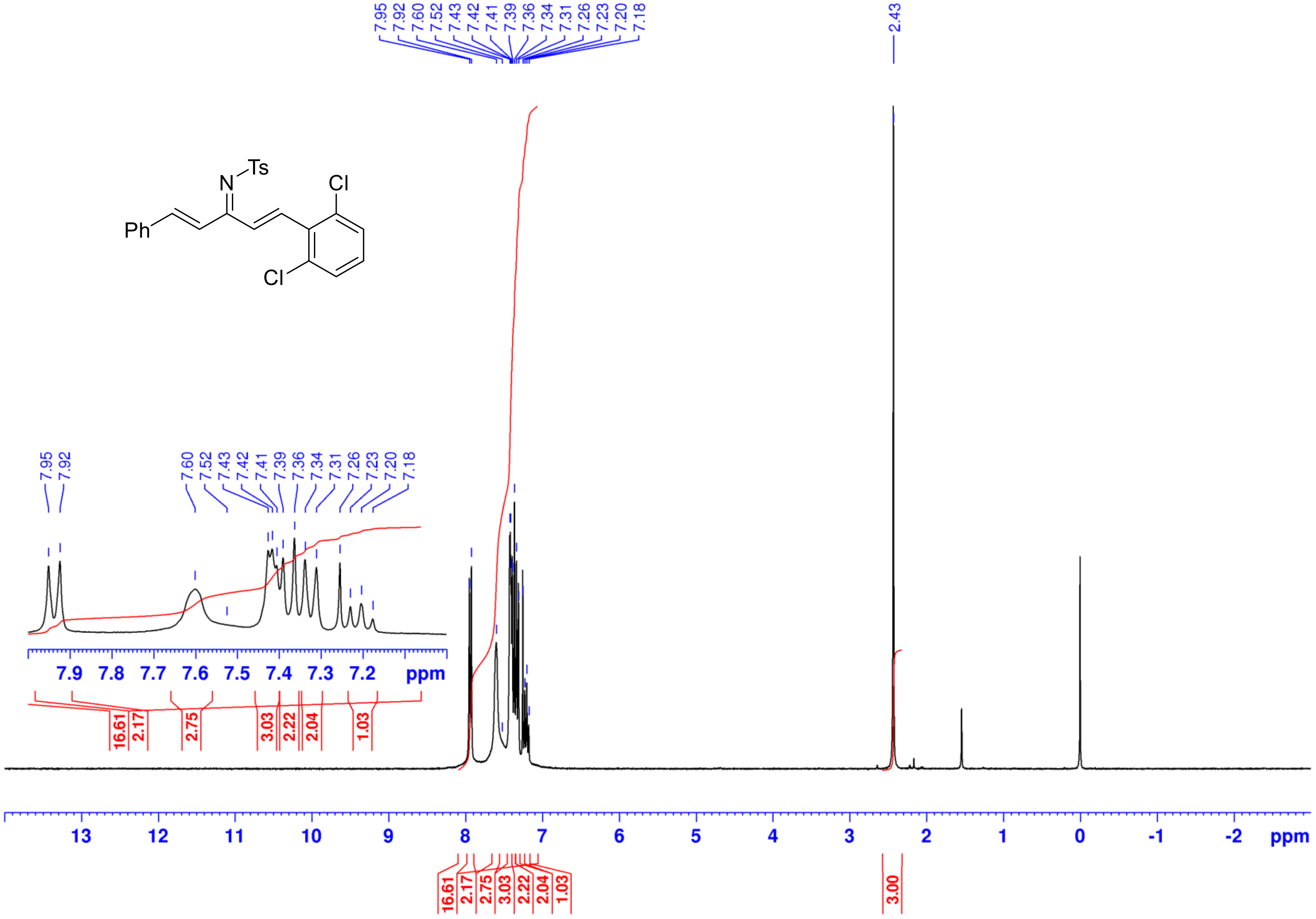

^{1}H NMR spectrum of **2d** (300.1 MHz, $CDCl_3$)

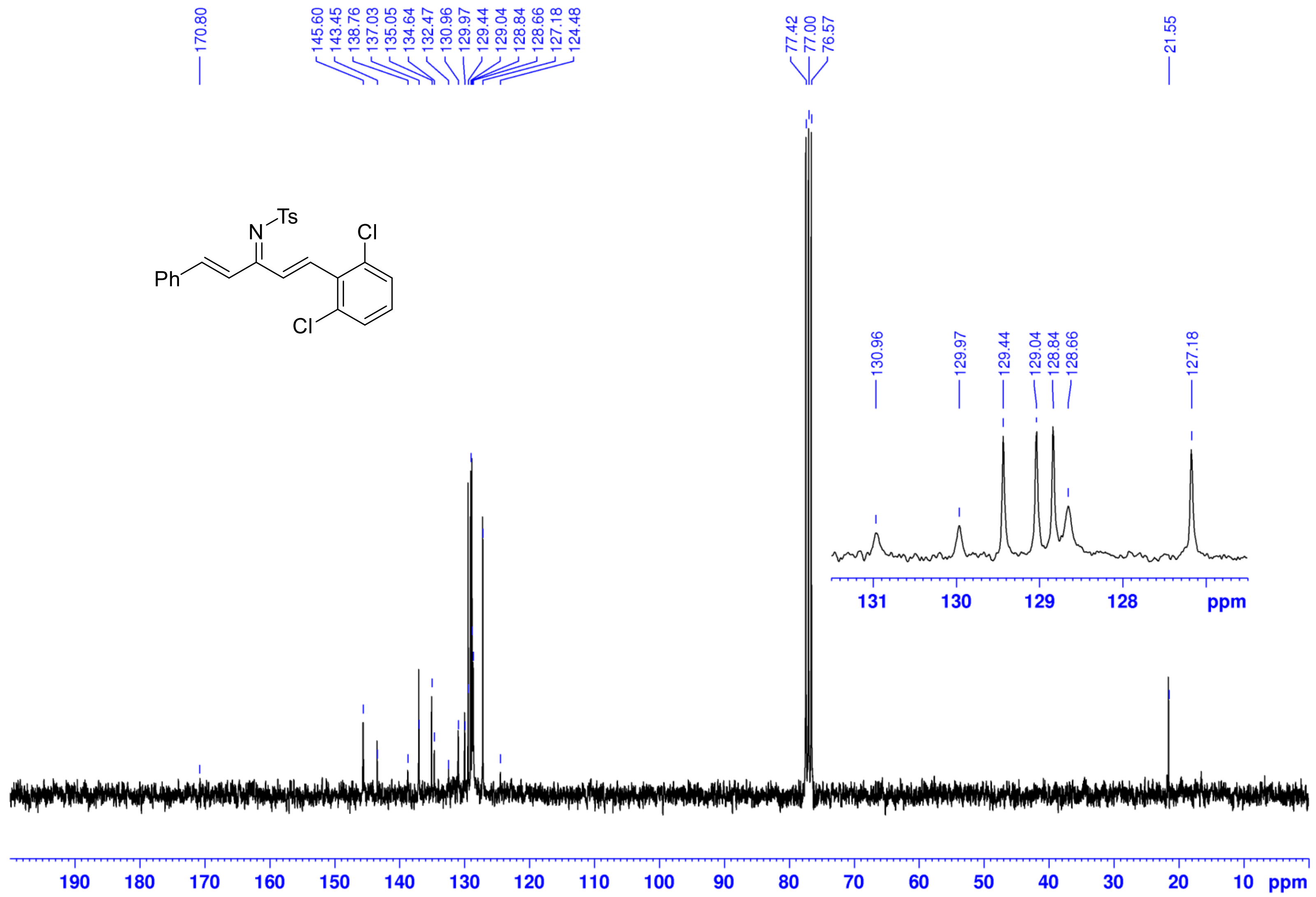

^{13}C NMR spectrum of **2d** (75.5 MHz, $CDCl_3$)

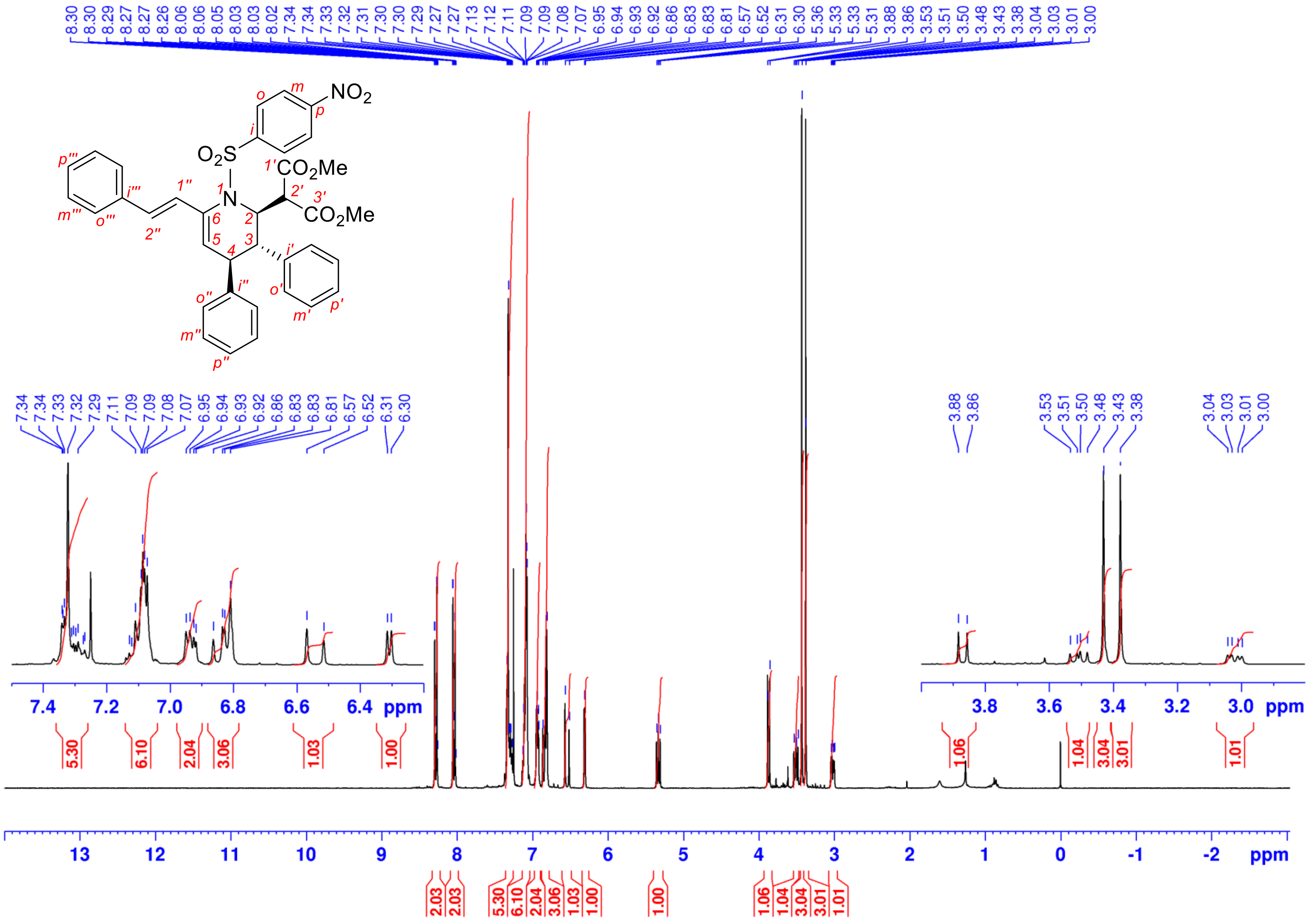

^{1}H NMR spectrum of **3a** (300.1 MHz, $CDCl_3$)

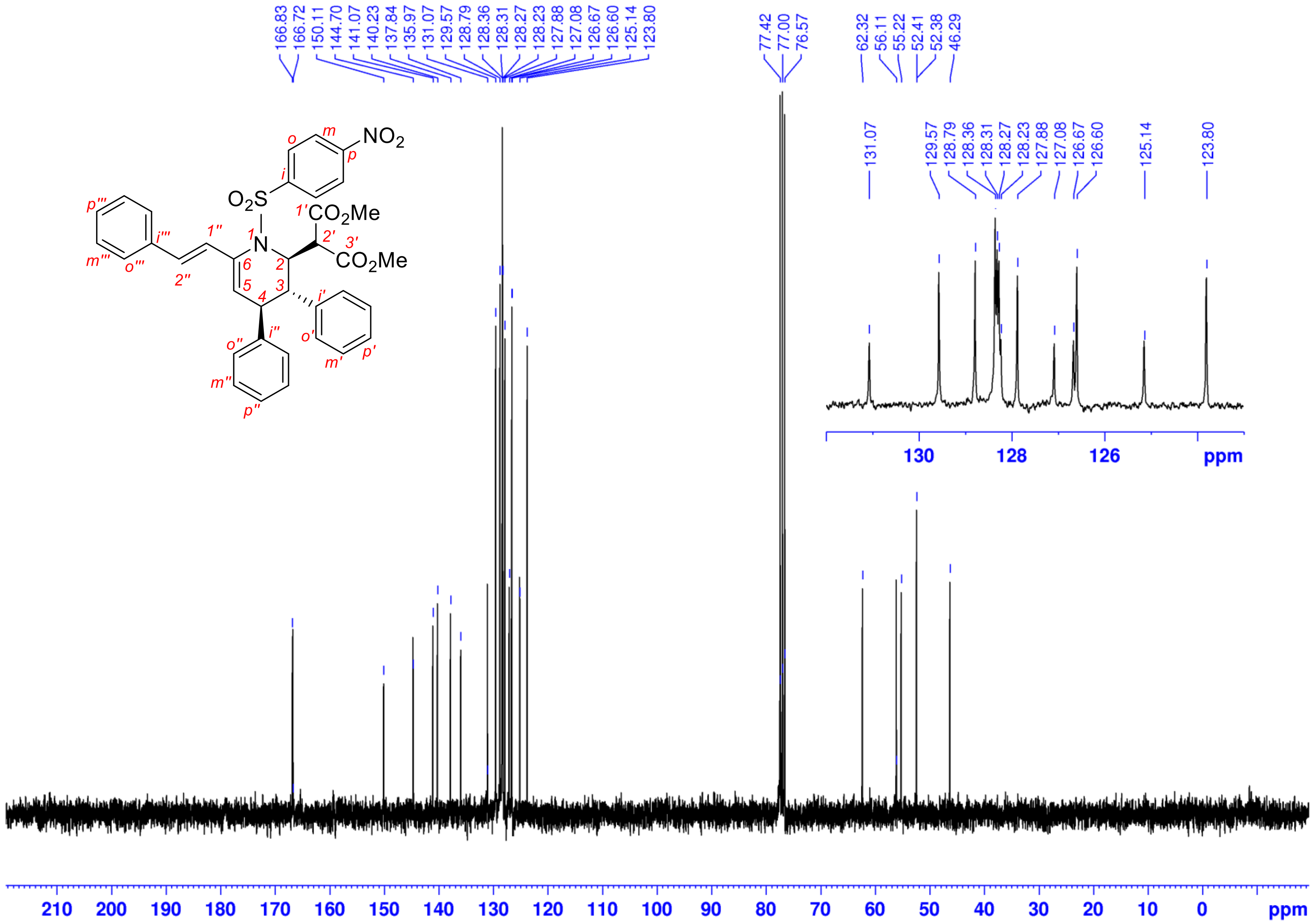

^{13}C NMR spectrum of **3a** (75.5 MHz, $CDCl_3$)

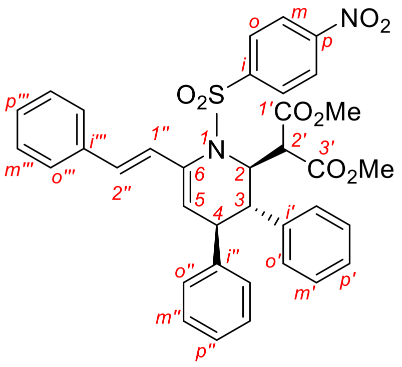

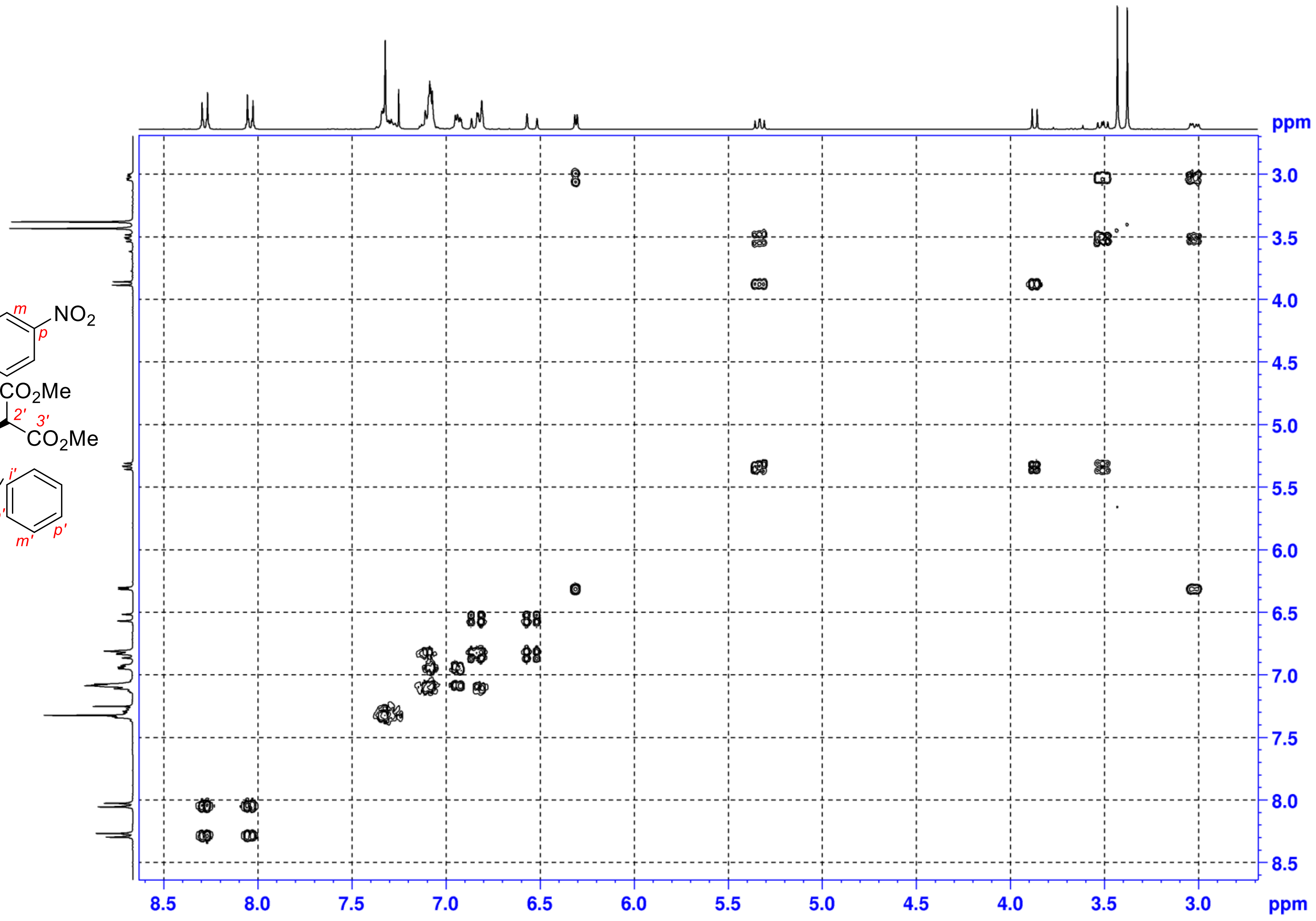

^{1}H,^{1}H-COSY NMR spectrum of **3a** (300.1 MHz, $CDCl_3$)

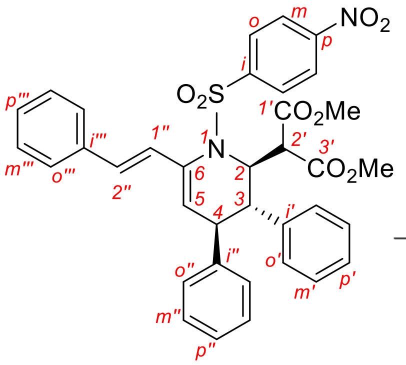

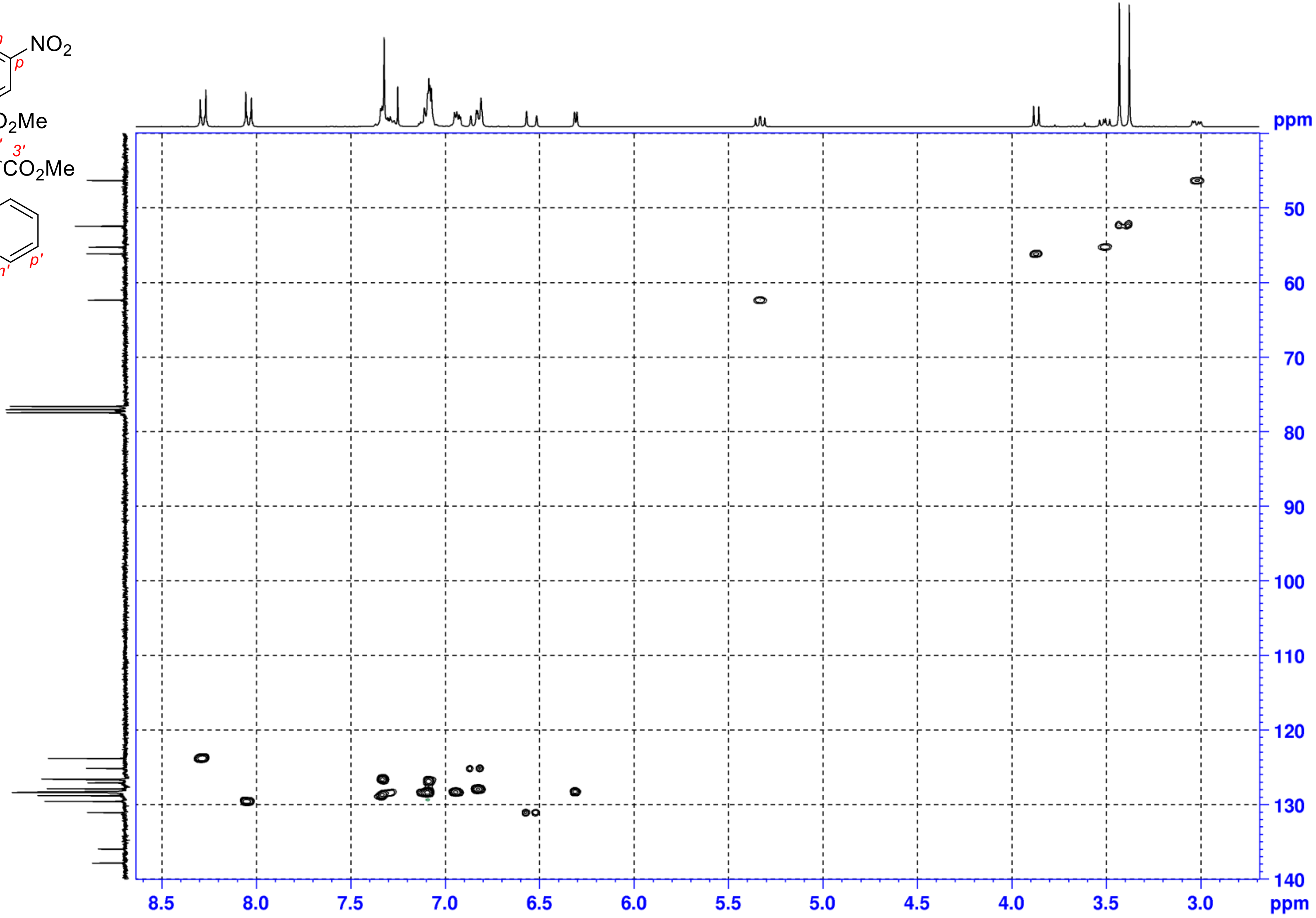

^{1}H,^{13}C-HSQC NMR spectrum of **3a** (^{1}H: 300.1 MHz; ^{13}C: 75.5 MHz; $CDCl_3$)

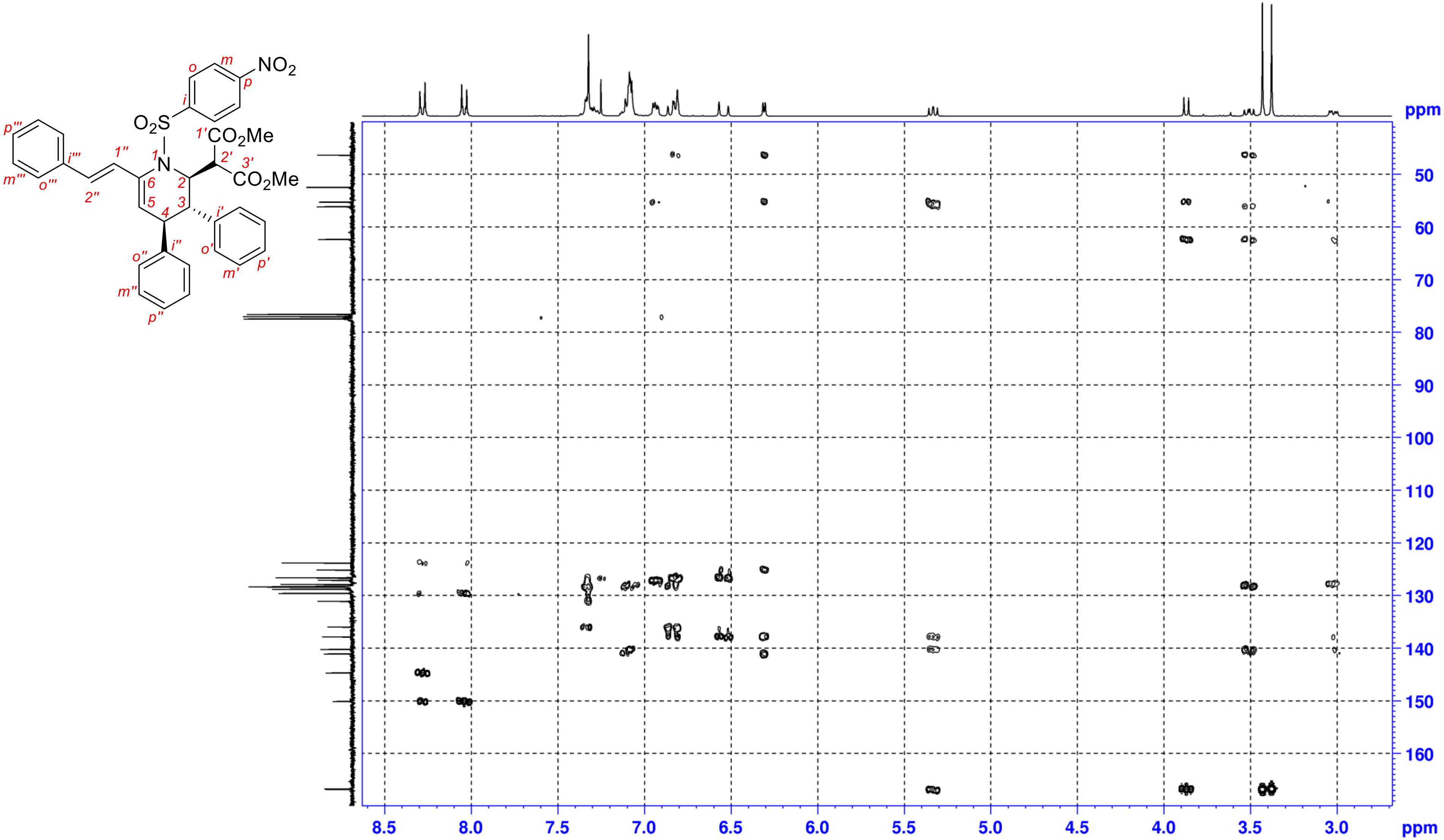

^{1}H,^{13}C-HMBC NMR spectrum of **3a** (^{1}H: 300.1 MHz; ^{13}C: 75.5 MHz; $CDCl_3$)

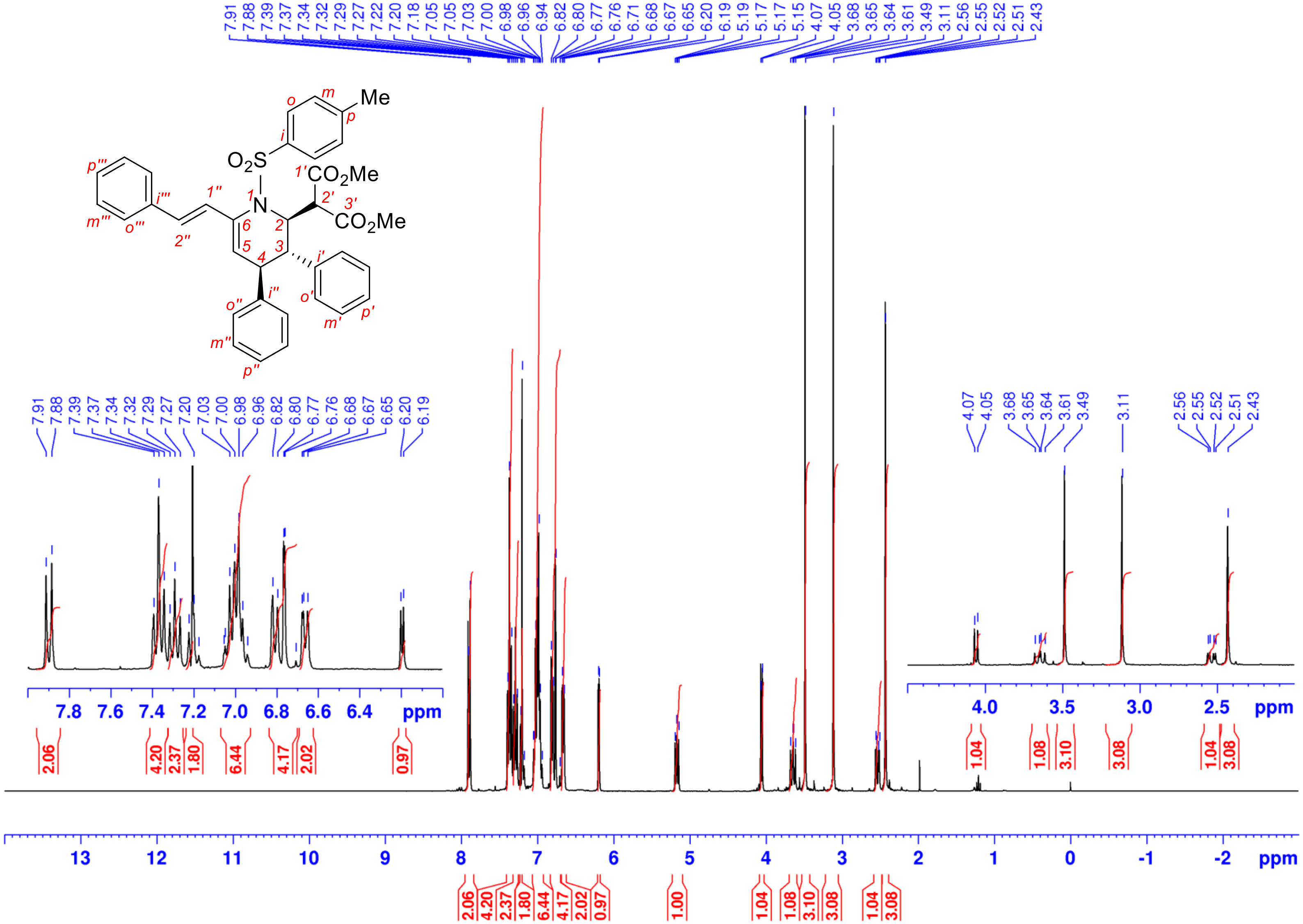

^{1}H NMR spectrum of **3b** (300.1 MHz, $CDCl_3$)

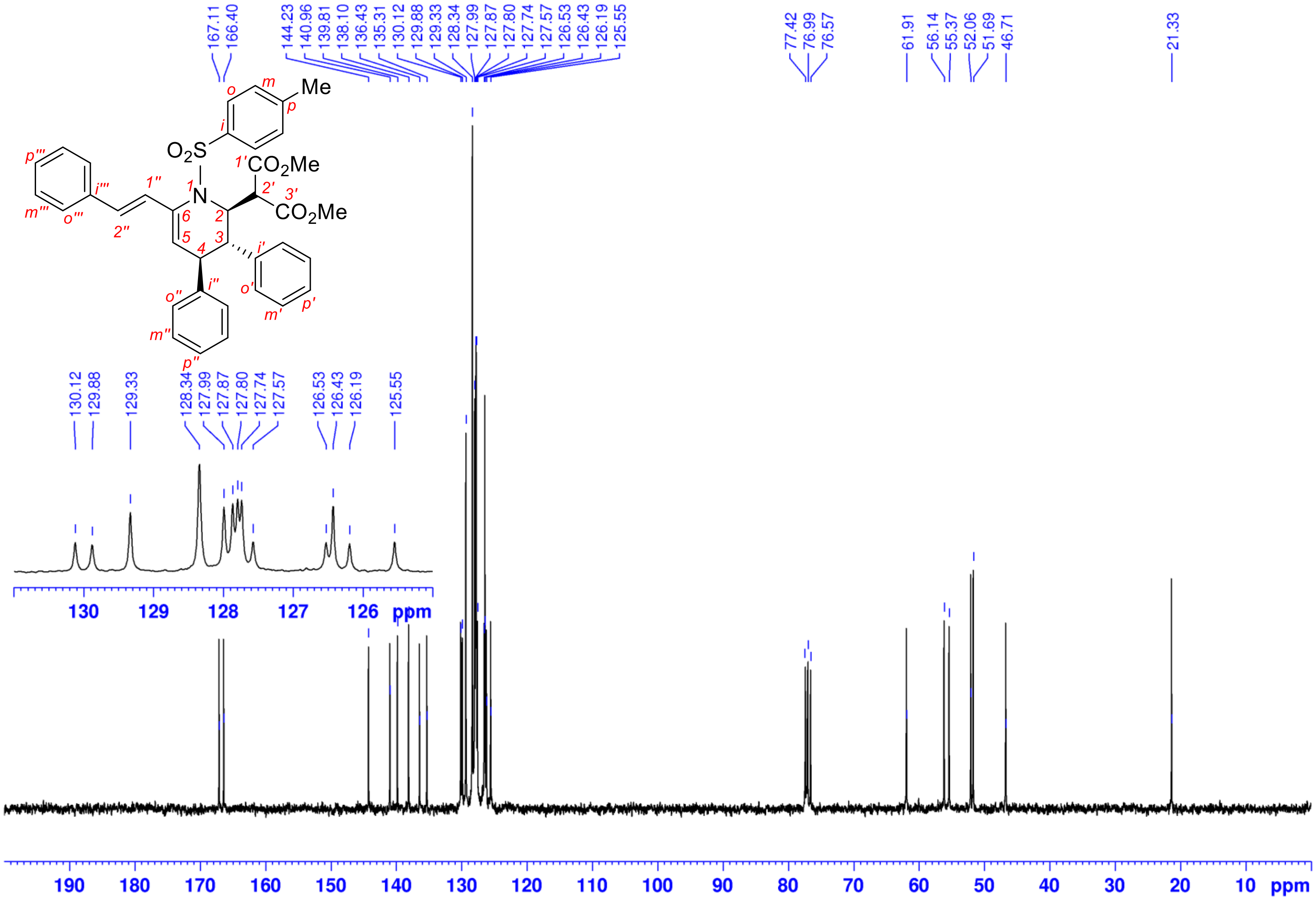

^{13}C NMR spectrum of **3b** (75.5 MHz, $CDCl_3$)

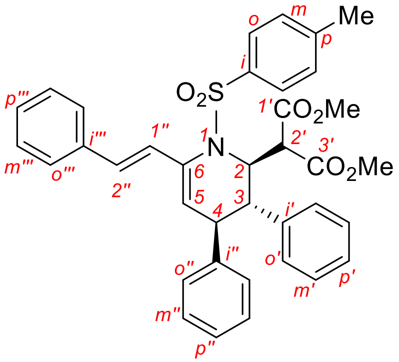

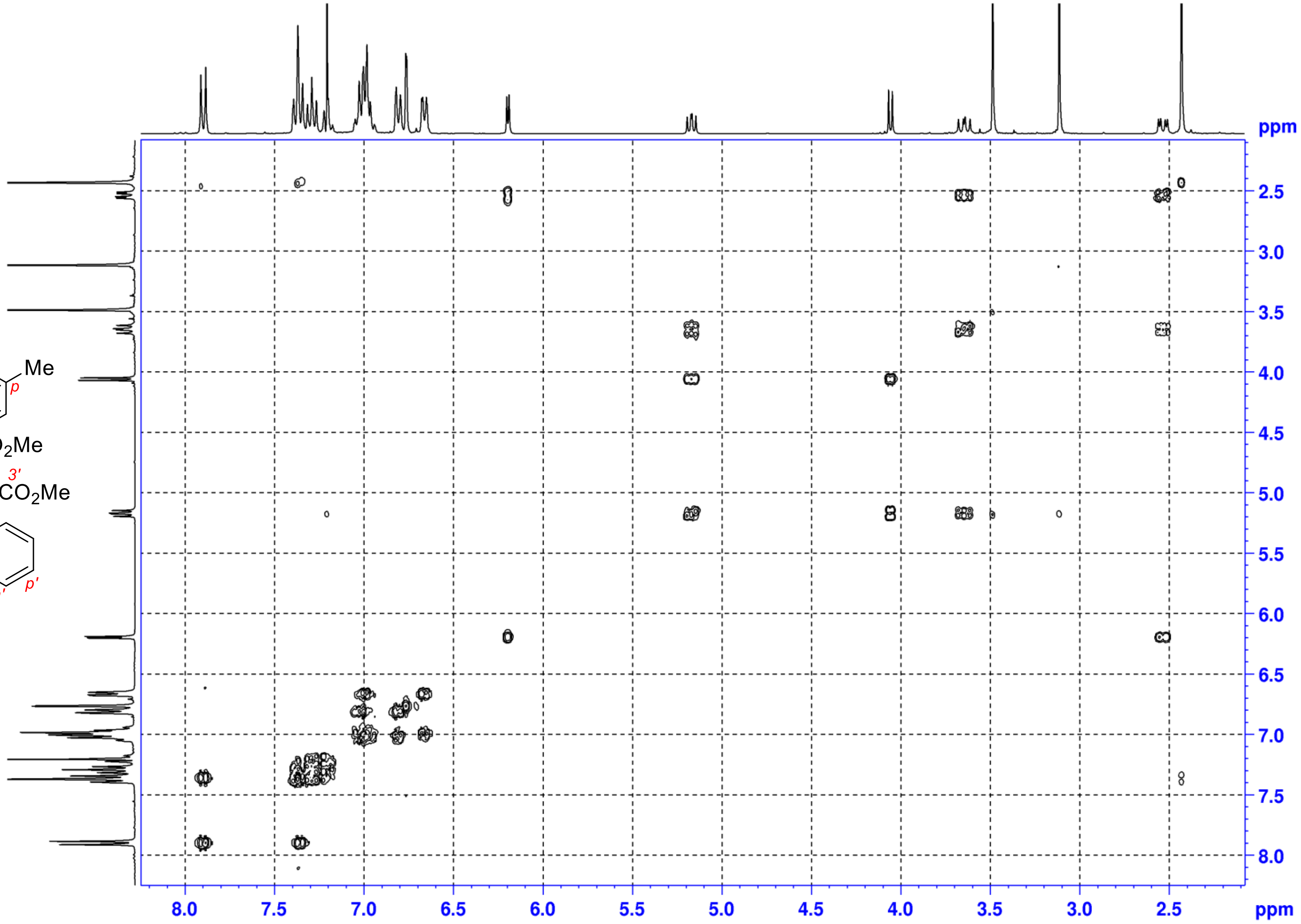

$^{1}H,^{1}H$-COSY NMR spectrum of **3b** (300.1 MHz, $CDCl_3$)

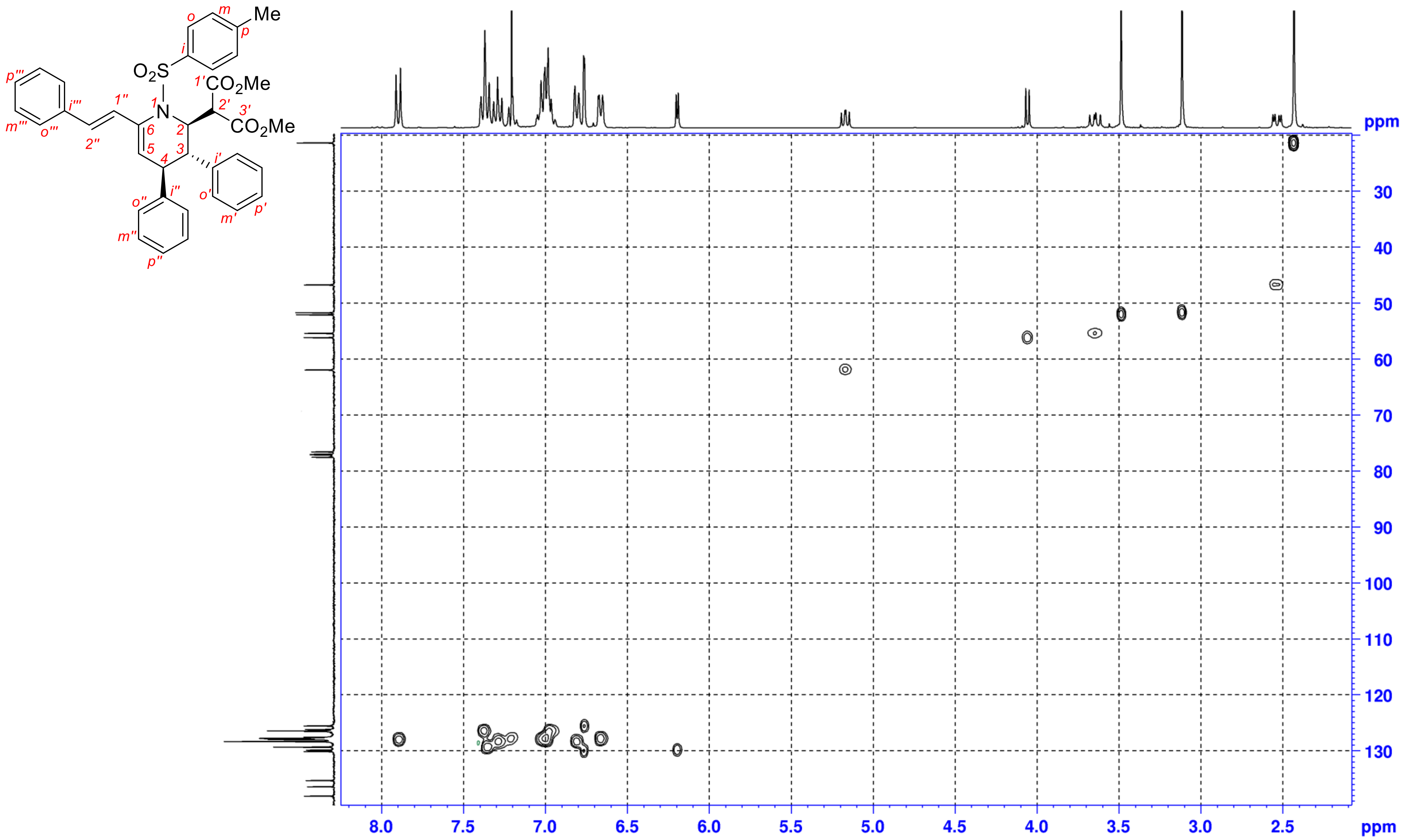

$^{1}H,^{13}C$-HSQC NMR spectrum of **3b** (^{1}H: 300.1 MHz; ^{13}C: 75.5 MHz; $CDCl_3$)

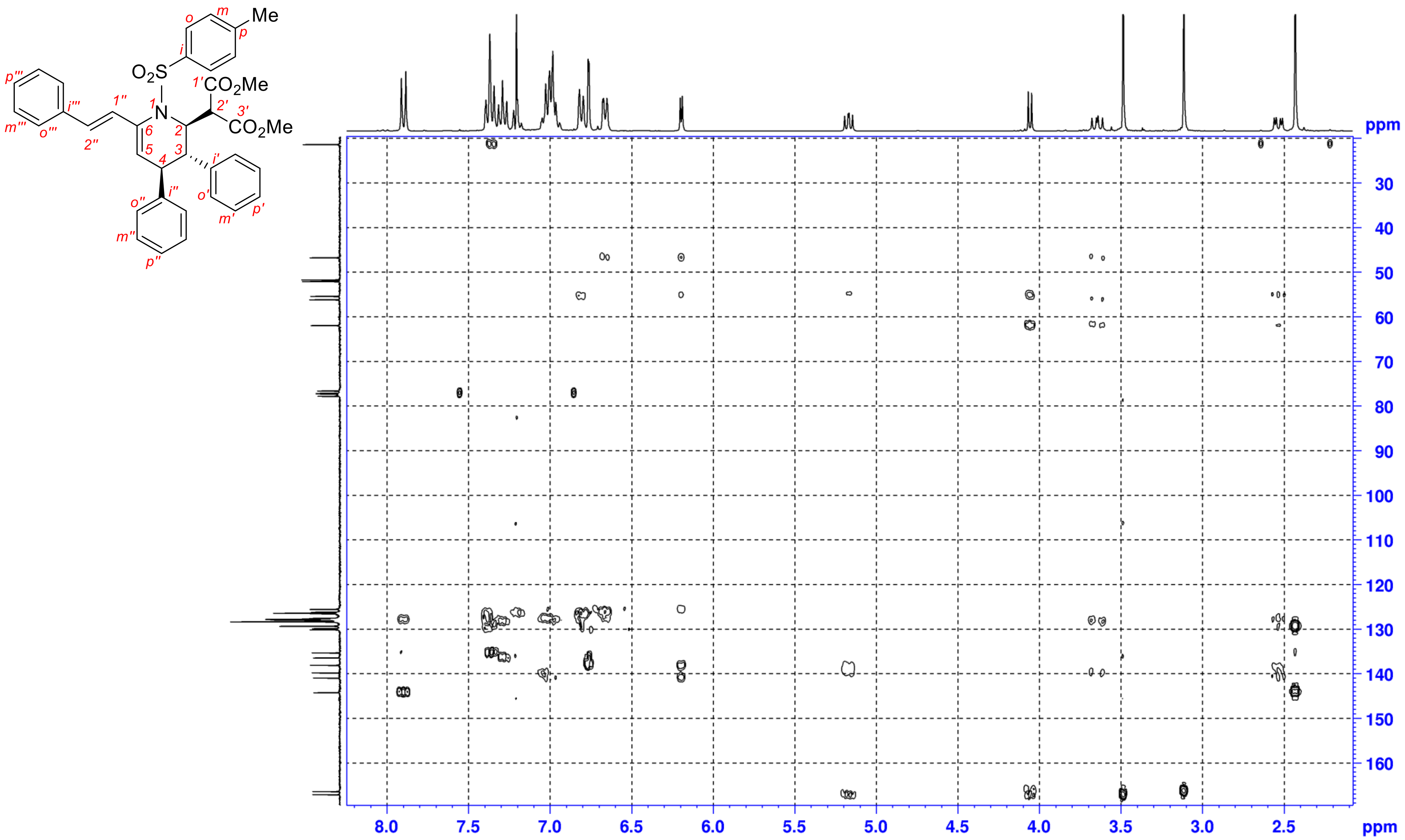

$^{1}H,^{13}C$-HMBC NMR spectrum of **3b** (^{1}H: 300.1 MHz; ^{13}C: 75.5 MHz; $CDCl_3$)

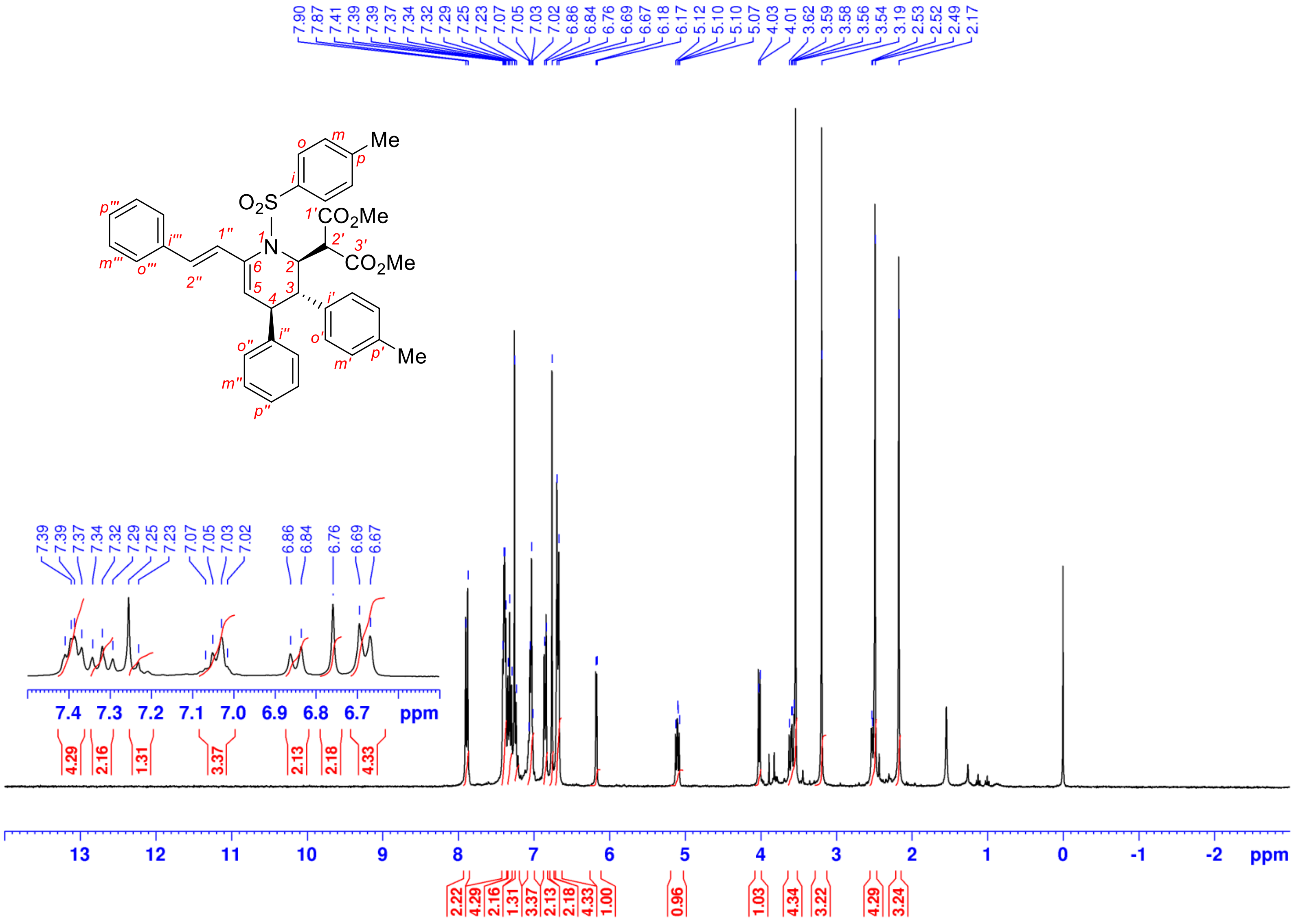

^{1}H NMR spectrum of **3c** (300.1 MHz, $CDCl_3$)

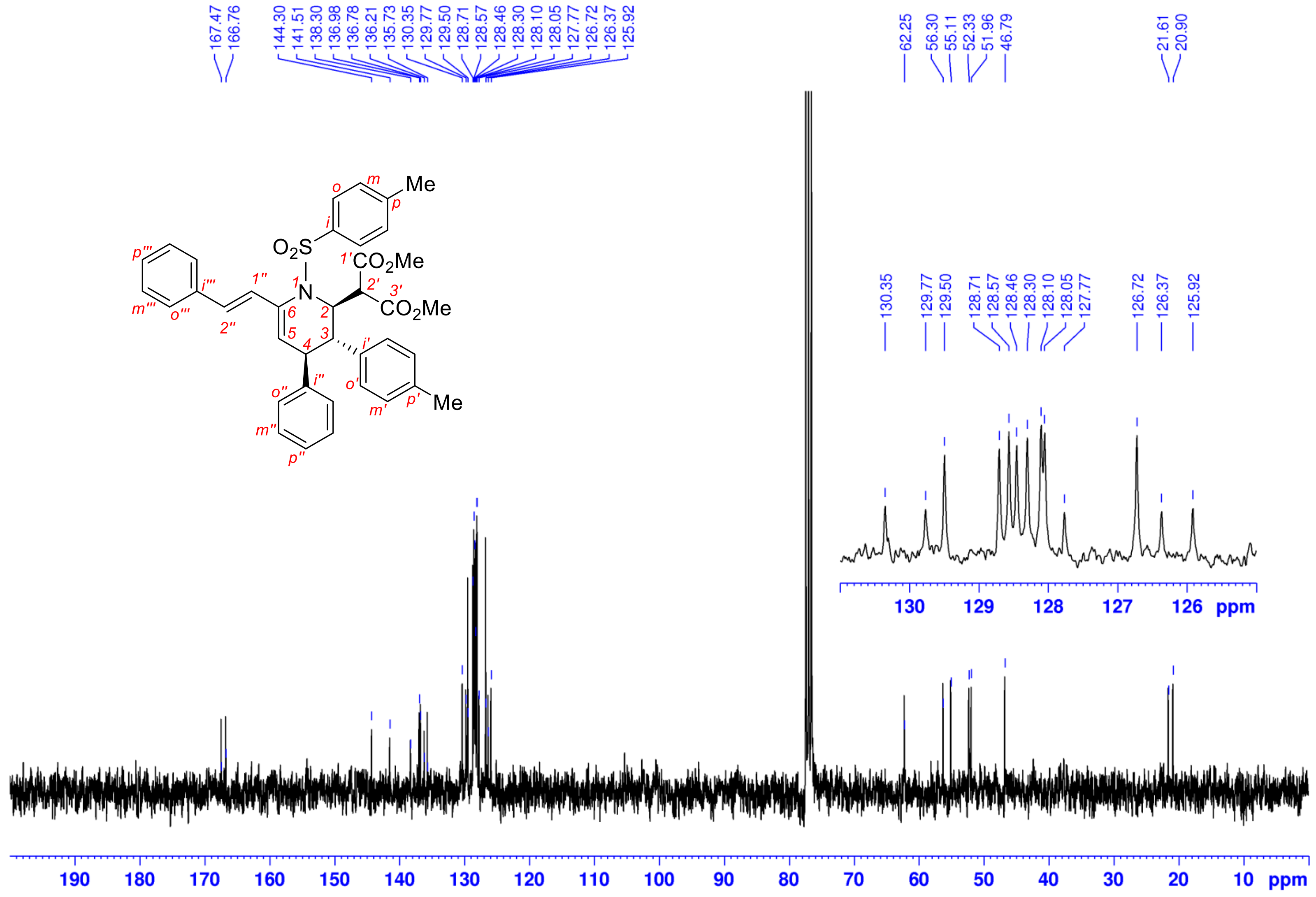

^{13}C NMR spectrum of **3c** (75.5 MHz, $CDCl_3$)

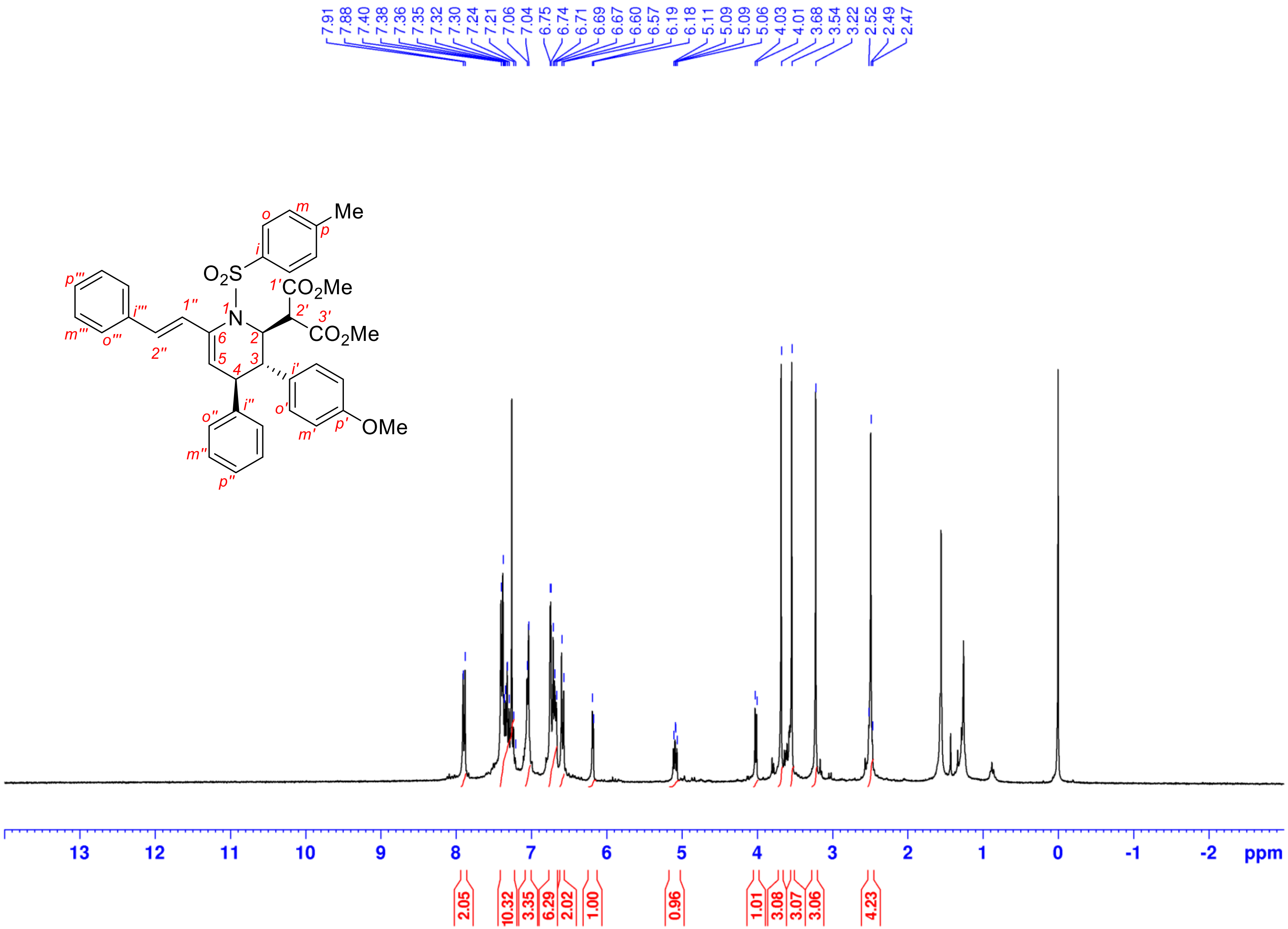

^{1}H NMR spectrum of **3d** (300.1 MHz, $CDCl_3$)

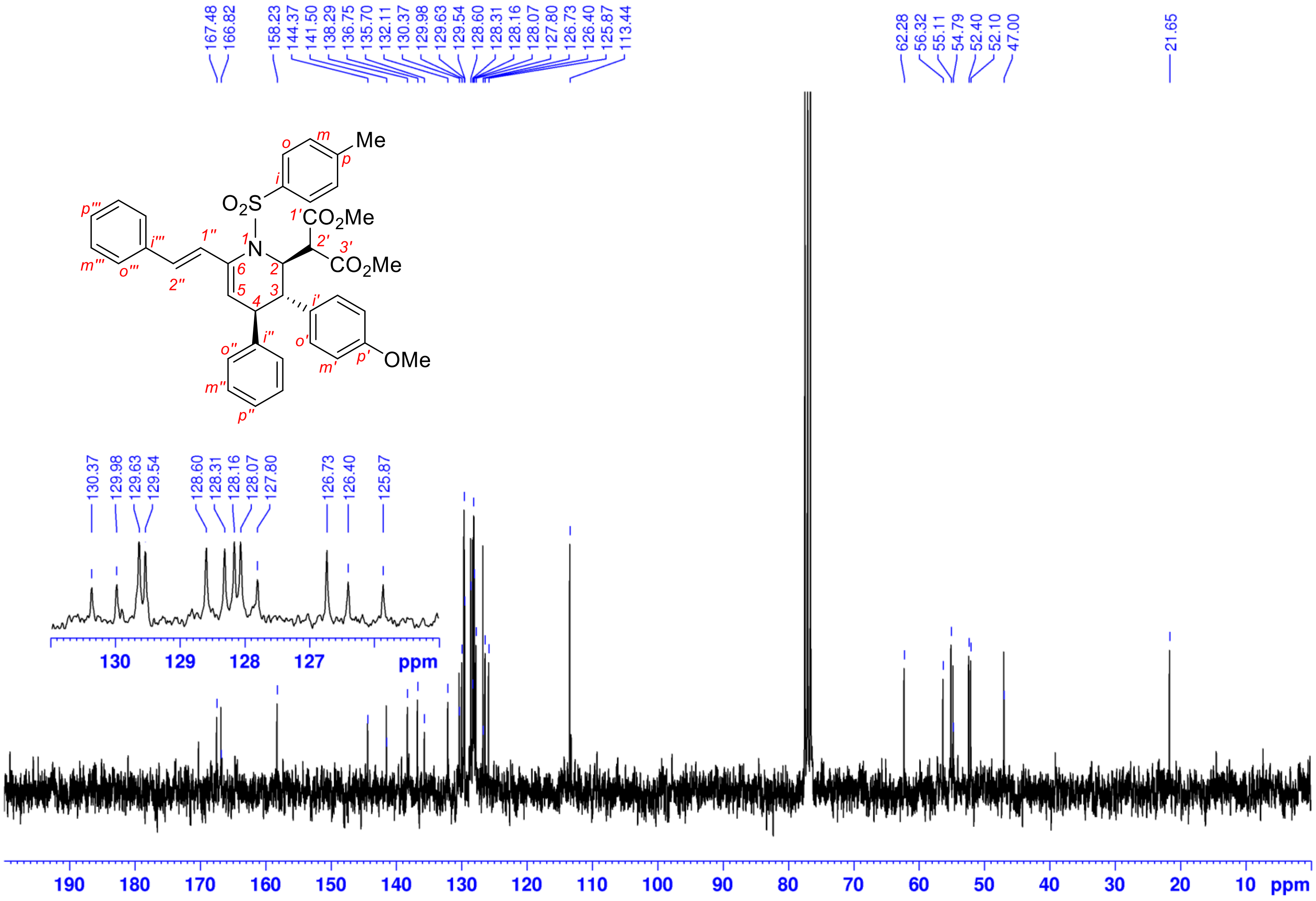

^{13}C NMR spectrum of **3d** (75.5 MHz, $CDCl_3$)

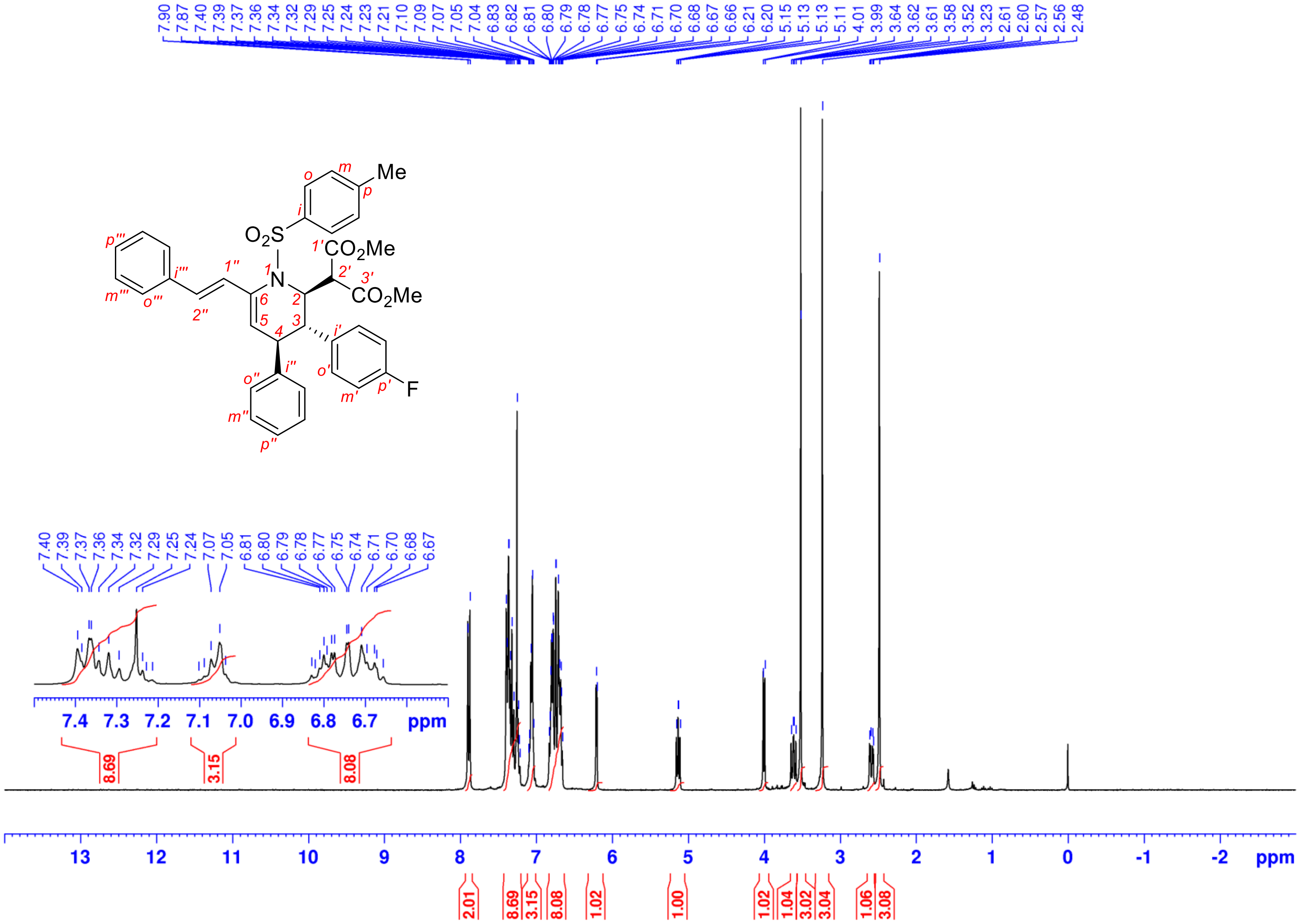

^{1}H NMR spectrum of **3e** (300.1 MHz, $CDCl_3$)

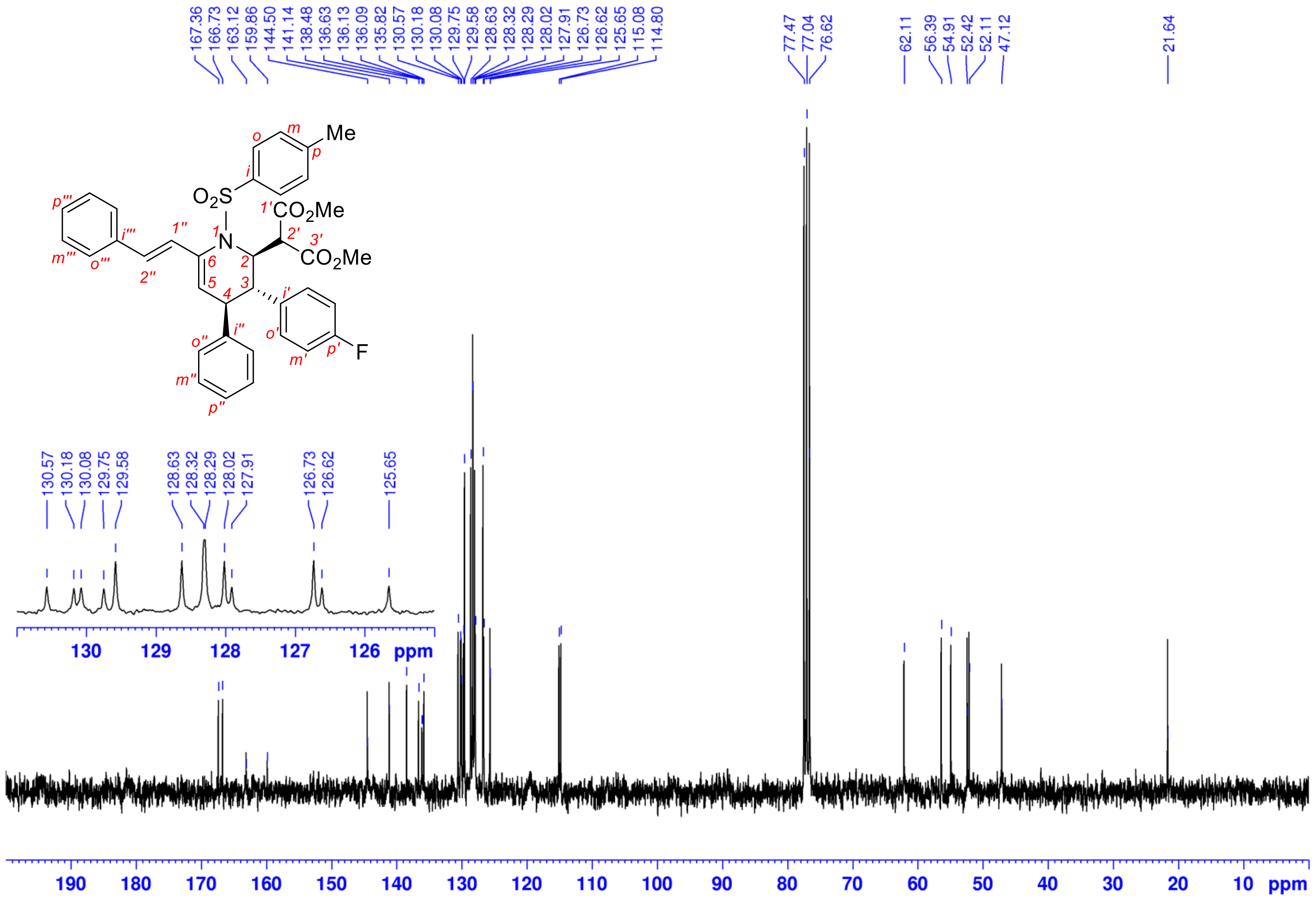

^{13}C NMR spectrum of **3e** (75.5 MHz, $CDCl_3$)

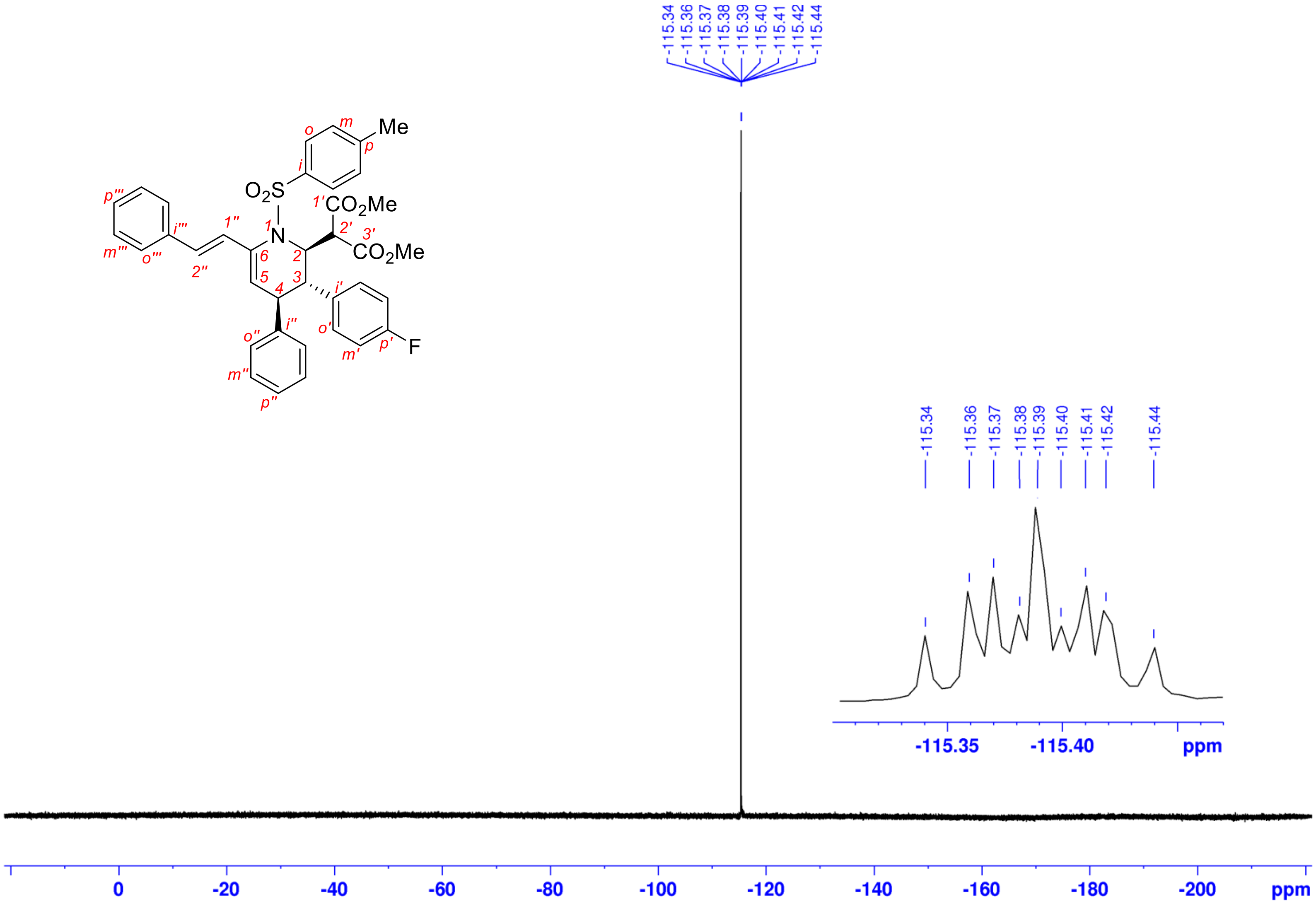

^{19}F NMR spectrum of **3e** (282.5 MHz, $CDCl_3$)

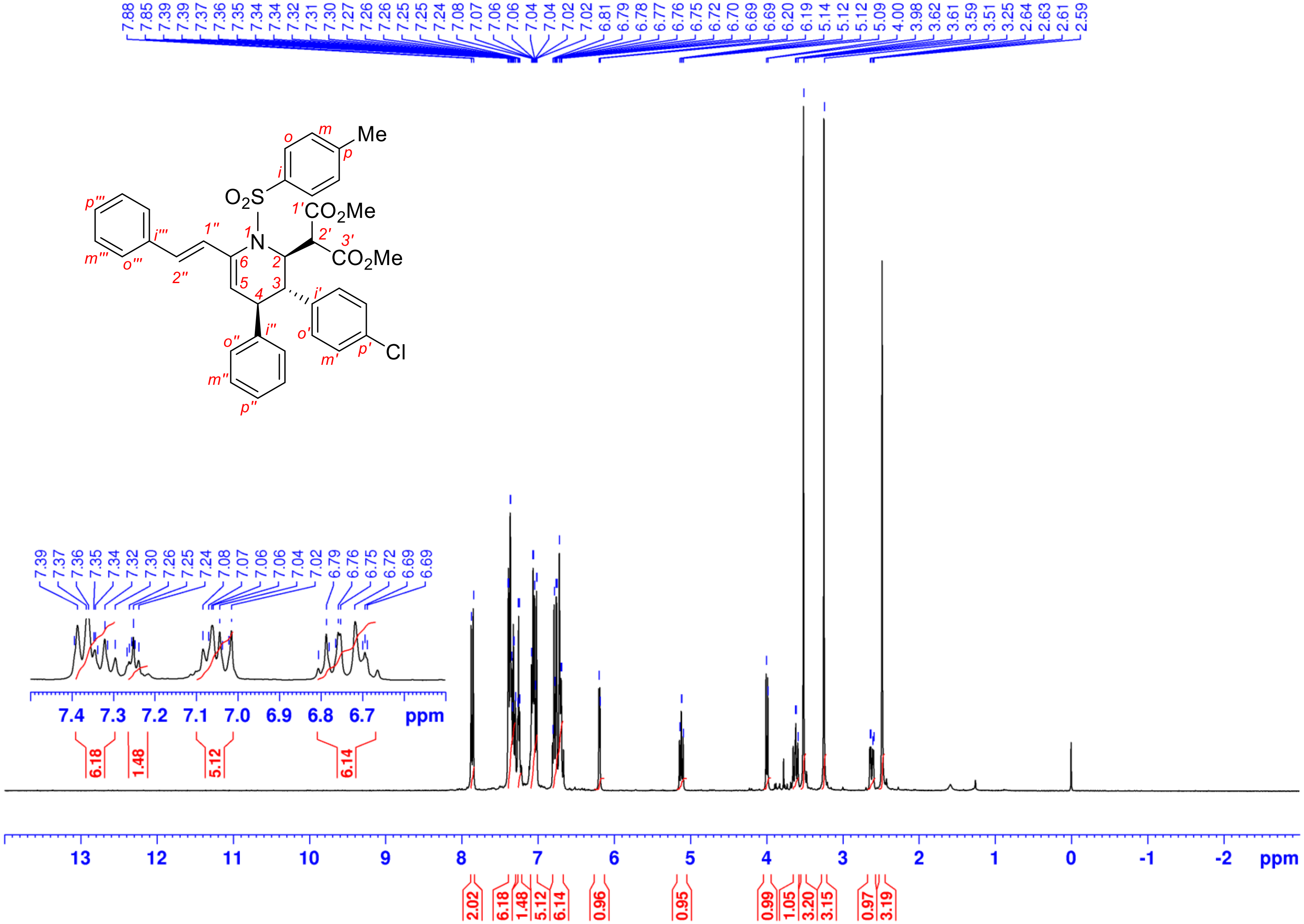

^{1}H NMR spectrum of **3f** (300.1 MHz, $CDCl_3$)

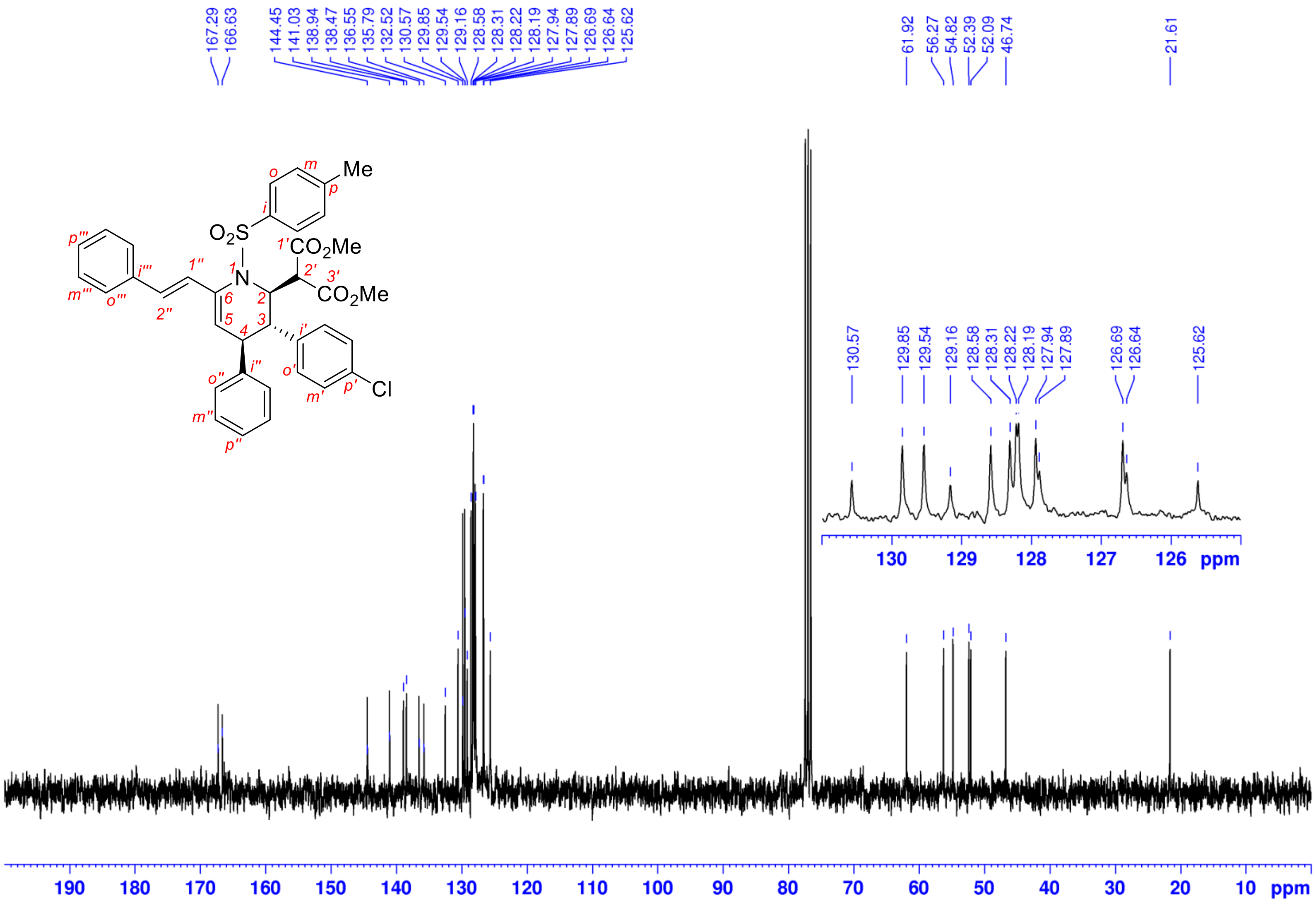

^{13}C NMR spectrum of **3f** (75.5 MHz, $CDCl_3$)

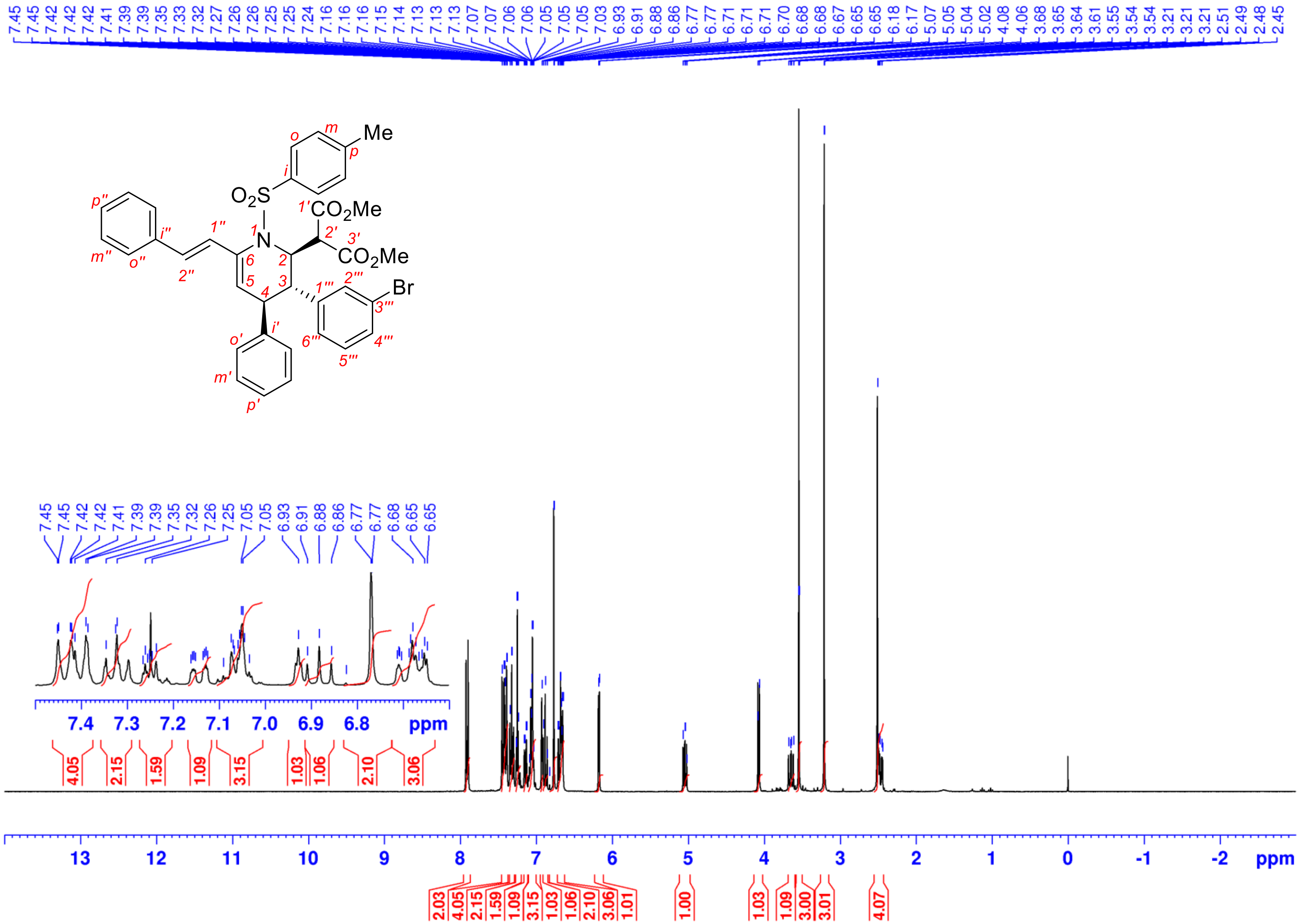

^{1}H NMR spectrum of **3g** (300.1 MHz, $CDCl_3$)

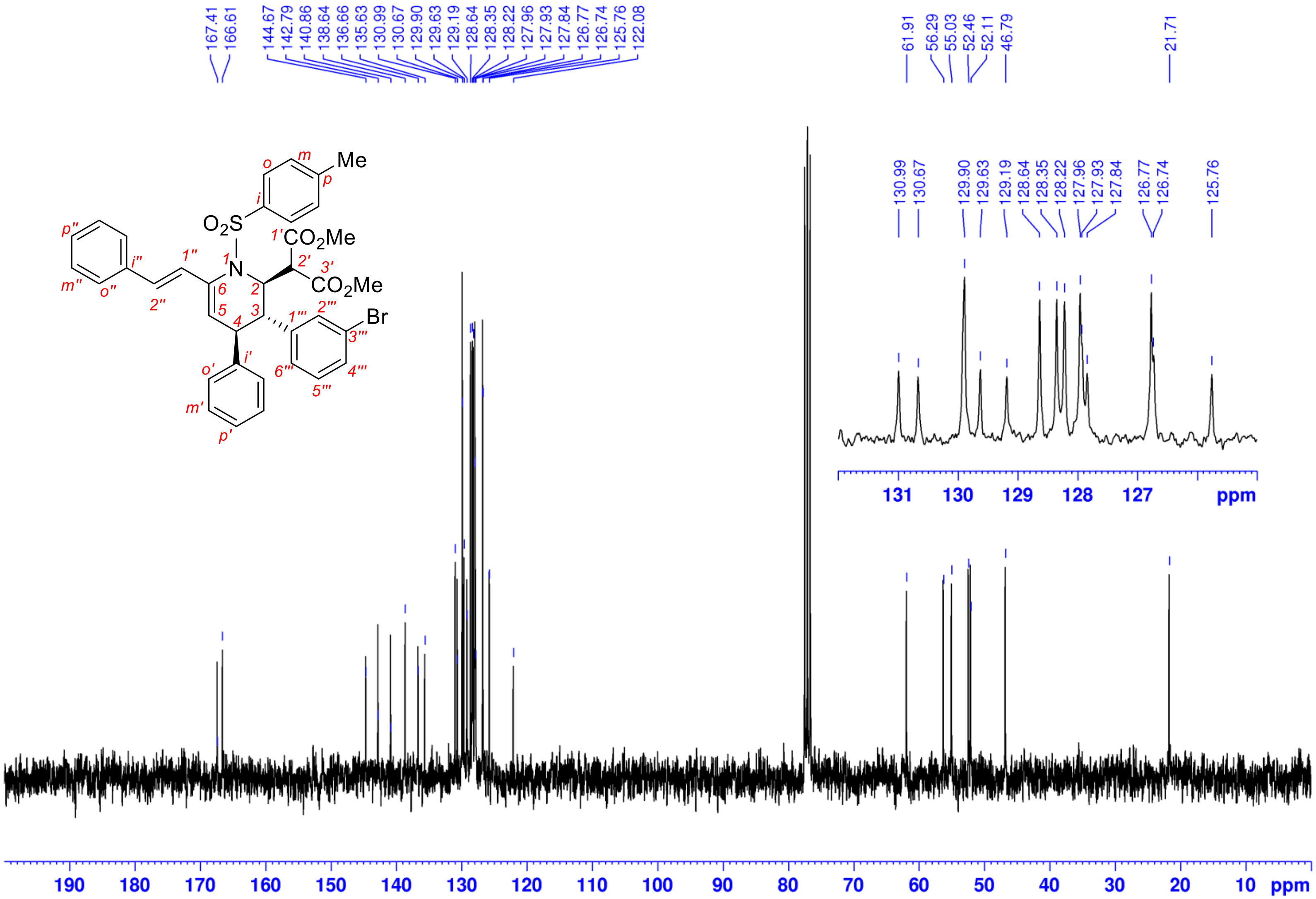

^{13}C NMR spectrum of **3g** (75.5 MHz, $CDCl_3$)

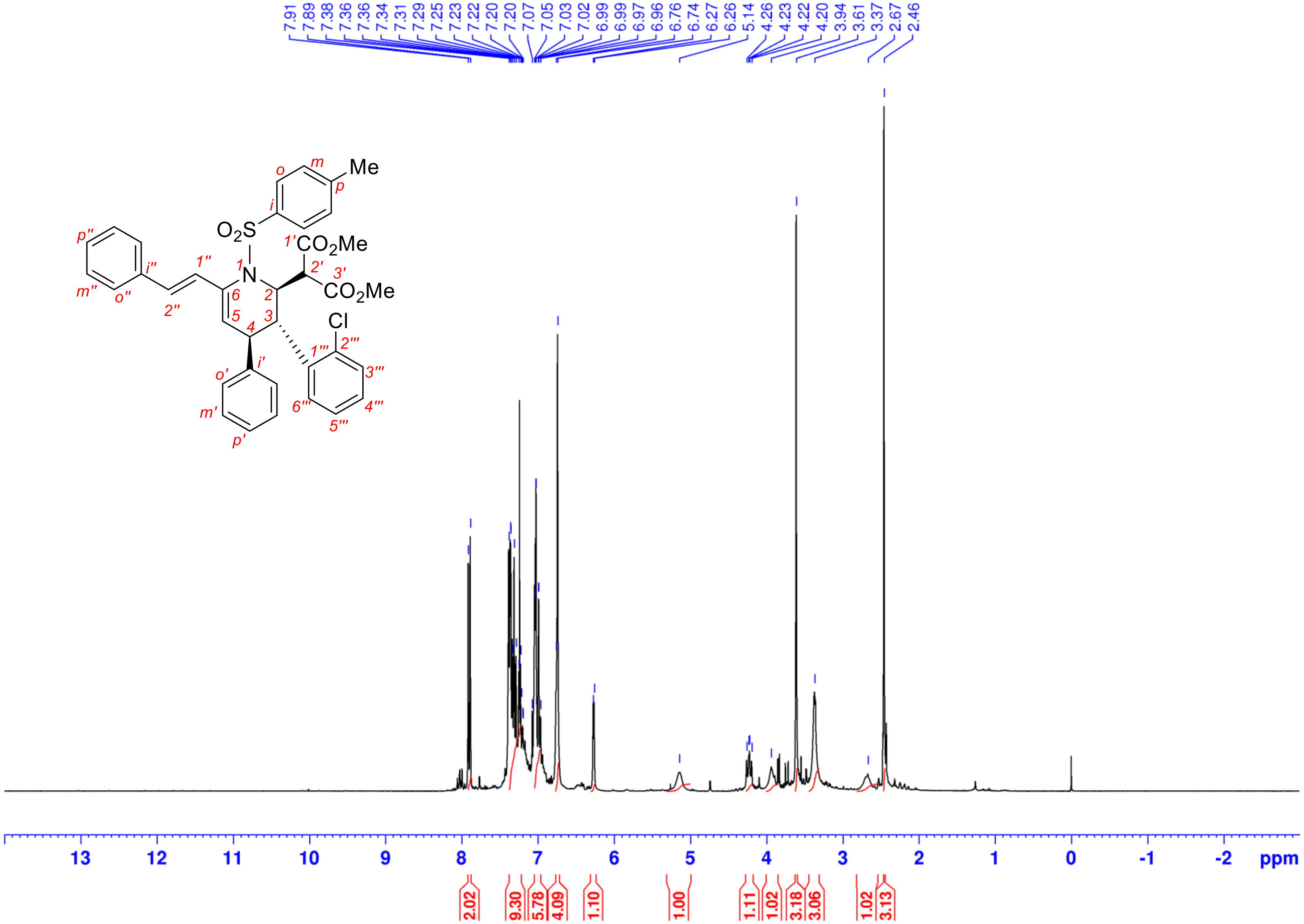

^{1}H NMR spectrum of **3h** (300.1 MHz, $CDCl_3$)

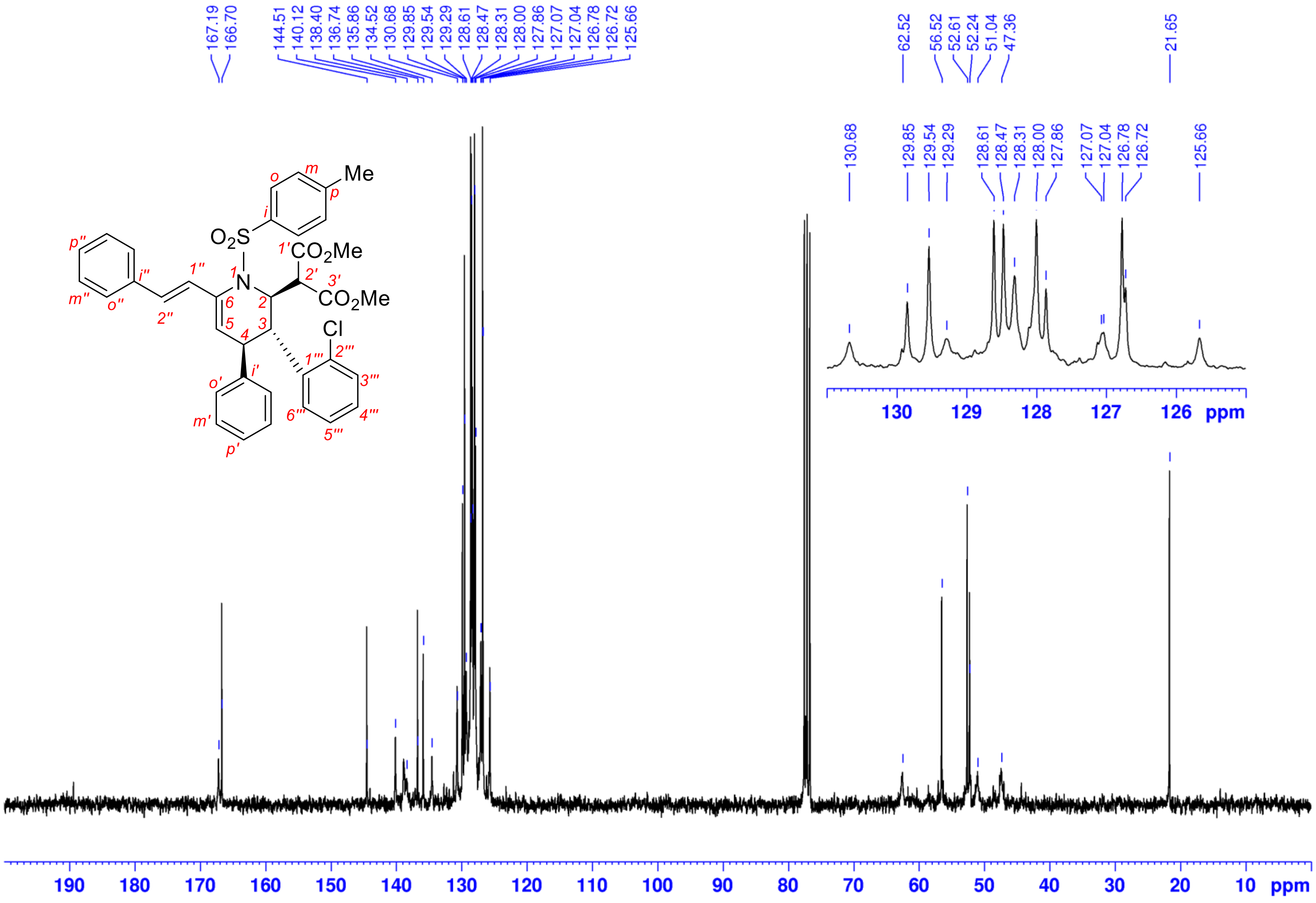

^{13}C NMR spectrum of **3h** (75.5 MHz, $CDCl_3$)

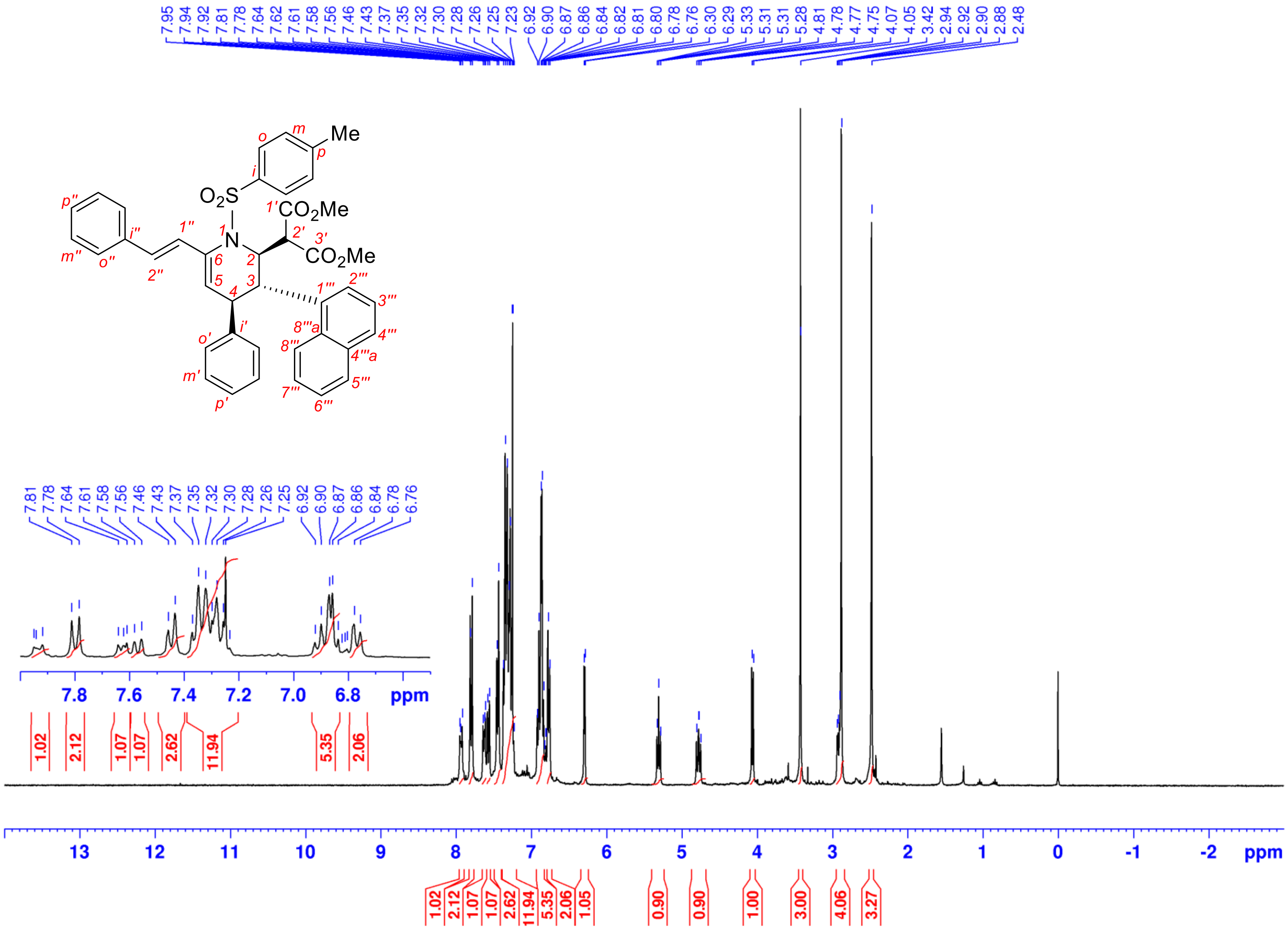

^{1}H NMR spectrum of **3i** (300.1 MHz, $CDCl_3$)

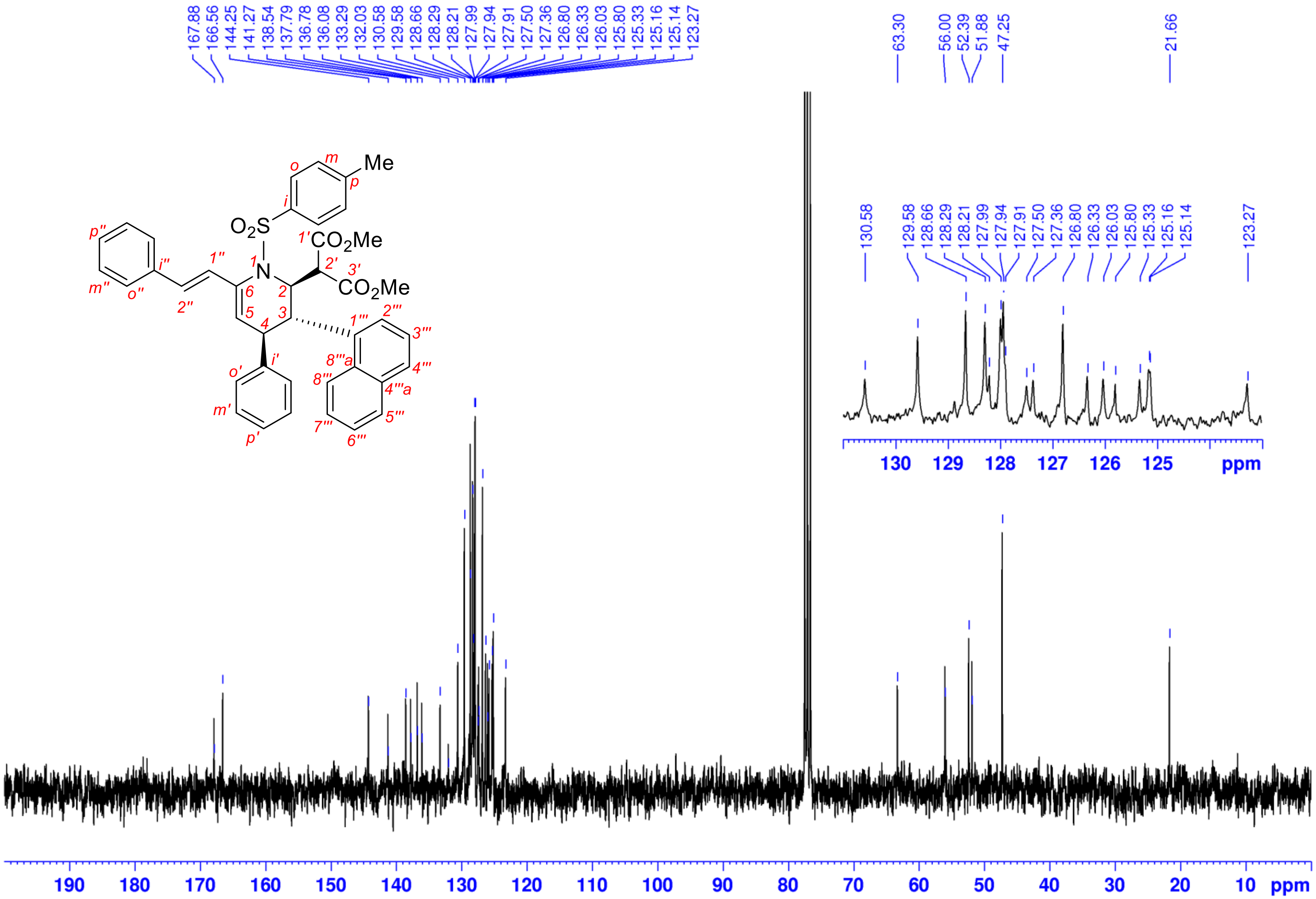

^{13}C NMR spectrum of **3i** (75.5 MHz, $CDCl_3$)

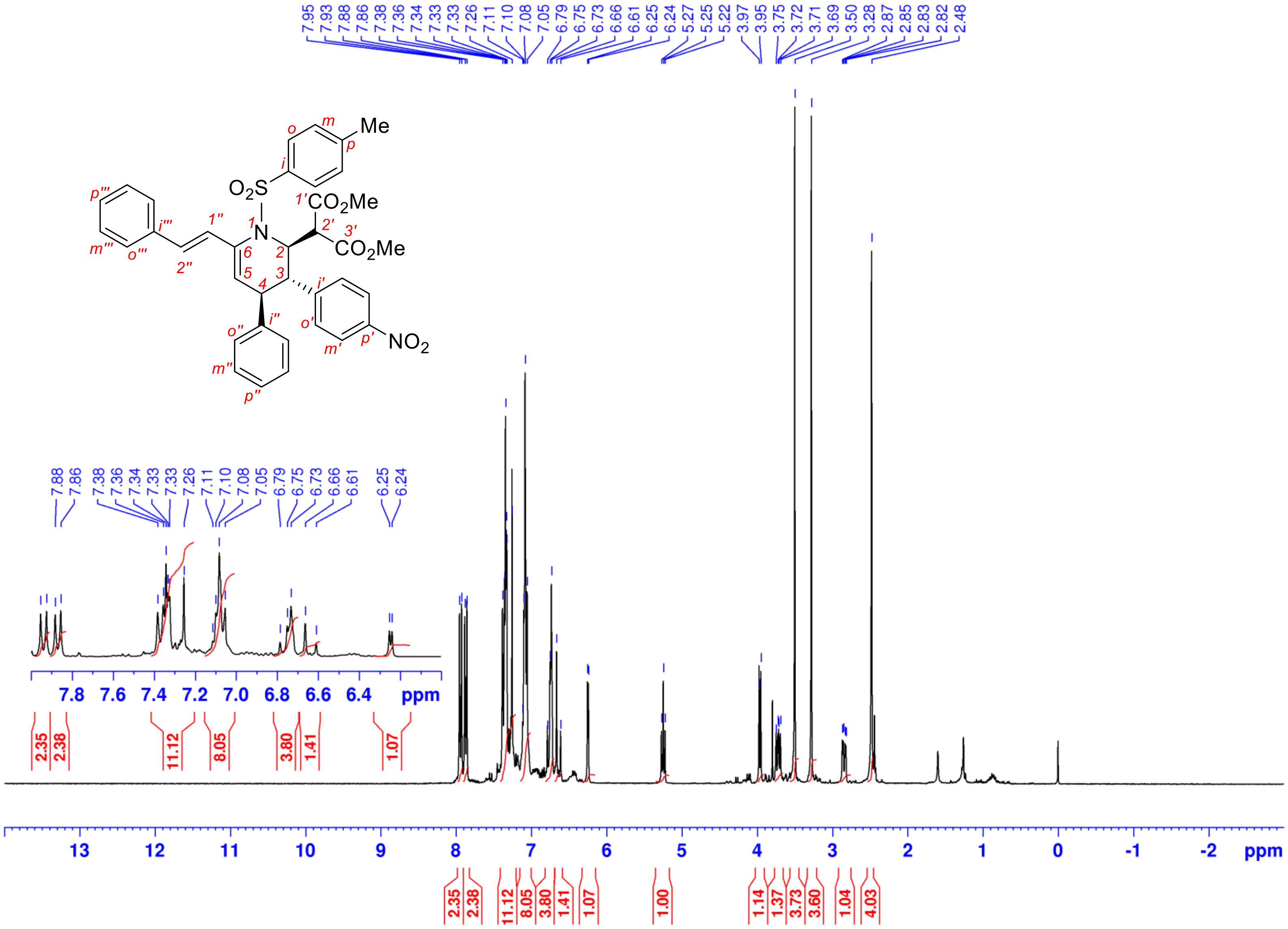

^{1}H NMR spectrum of **3j** (300.1 MHz, $CDCl_3$)

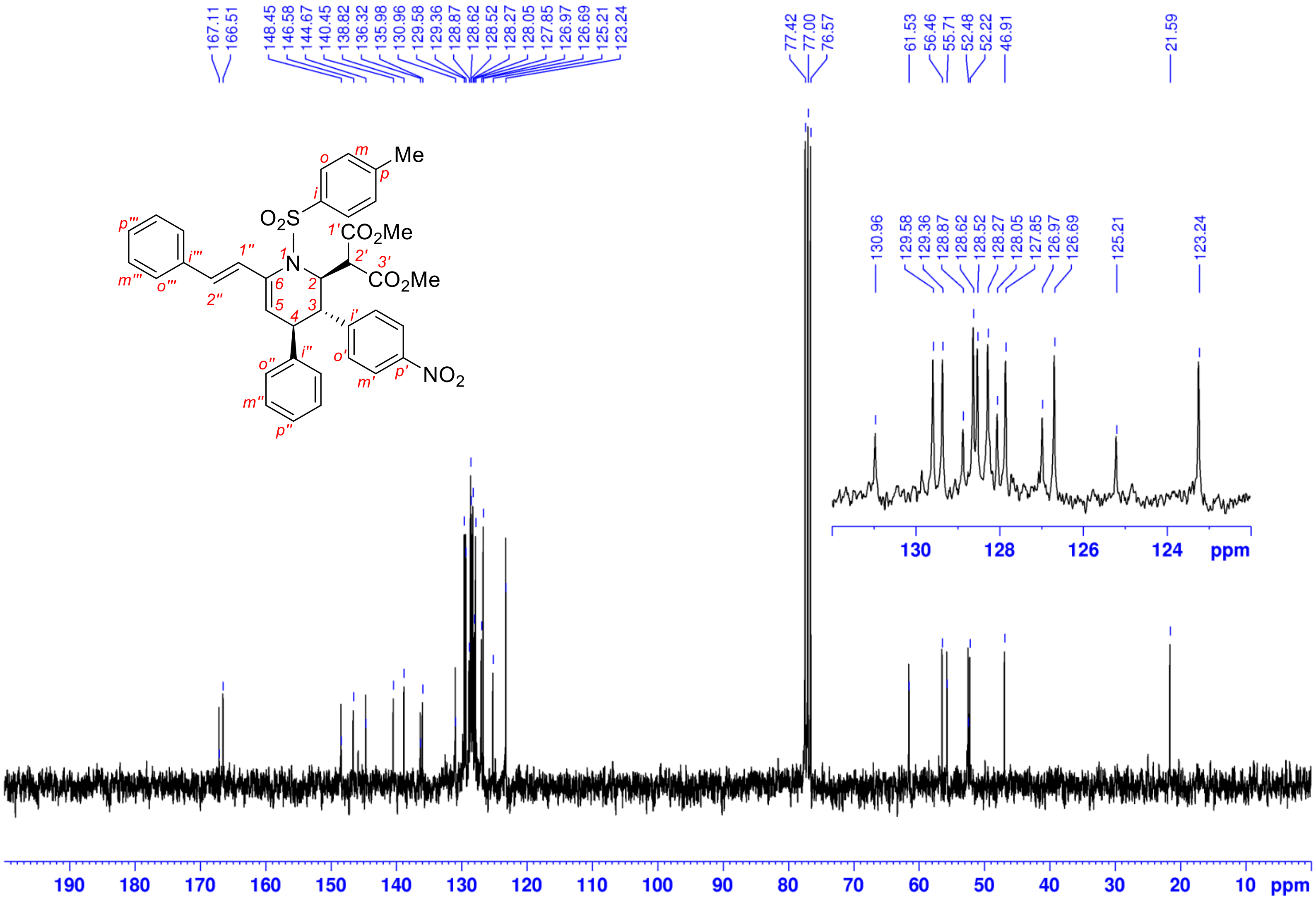

^{13}C NMR spectrum of **3j** (75.5 MHz, $CDCl_3$)

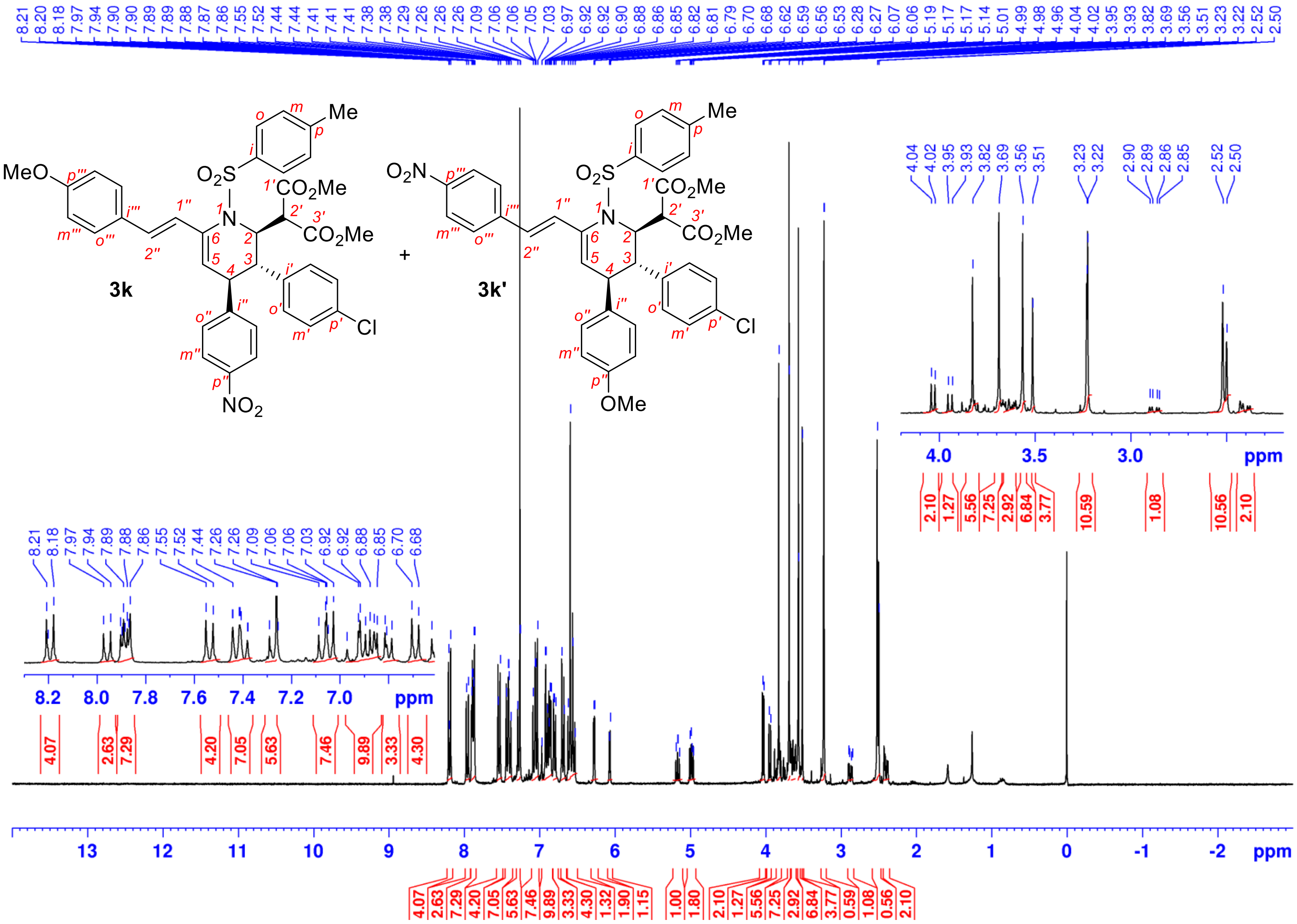

^{1}H NMR spectrum of **3k+3k'** (300.1 MHz, $CDCl_3$)

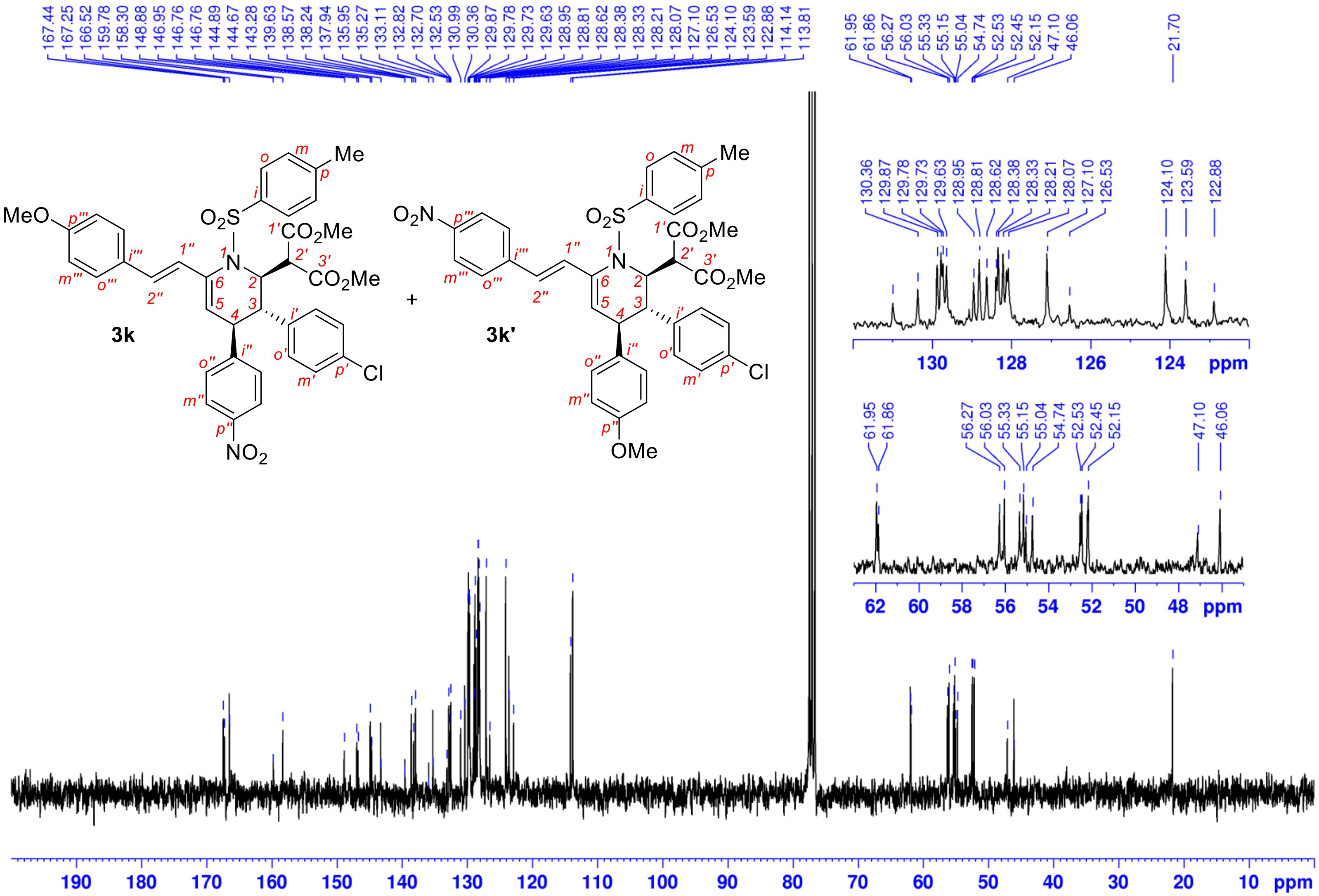

^{13}C NMR spectrum of **3k+3k'** (75.5 MHz, $CDCl_3$)

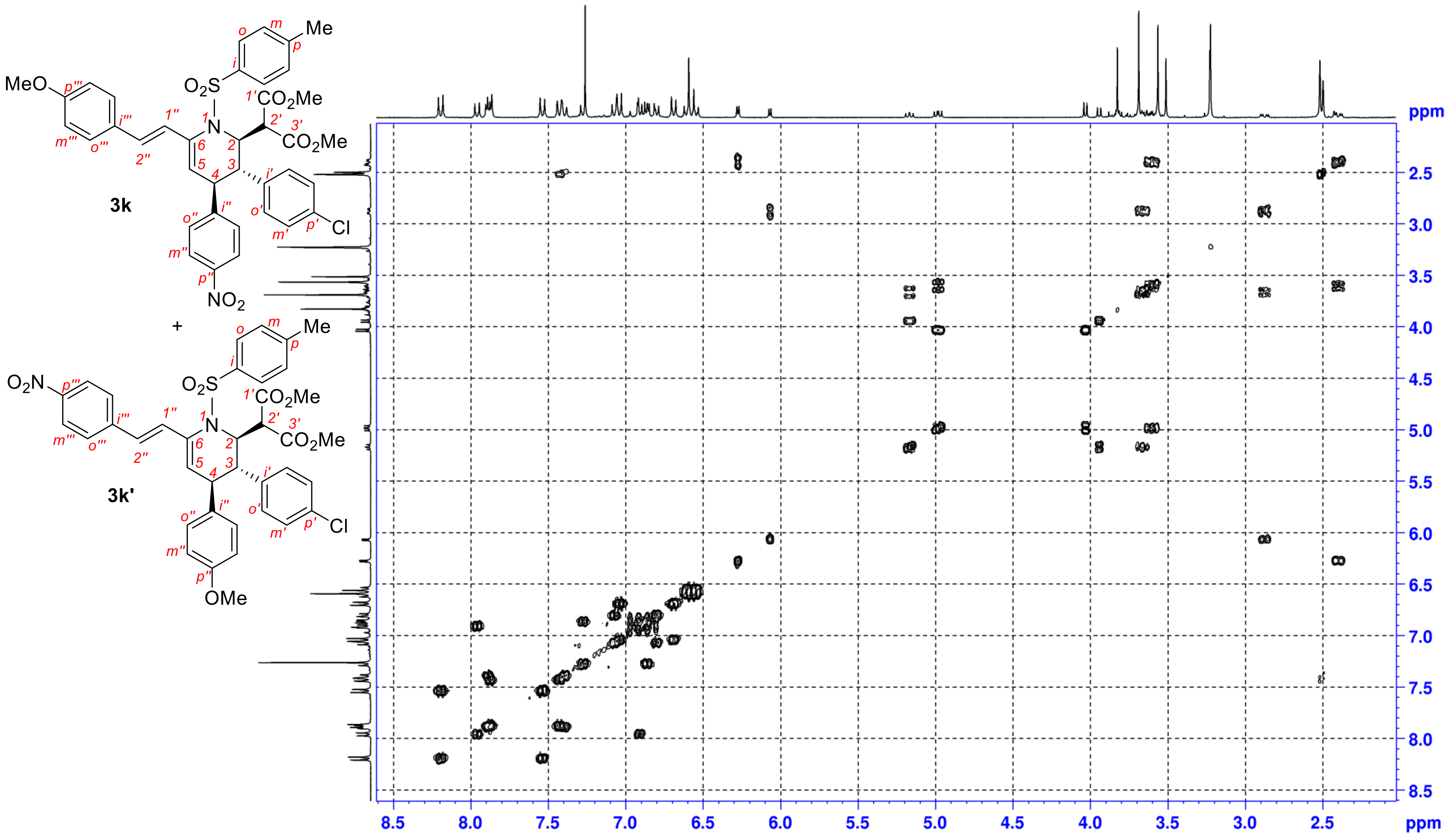

^{1}H,^{1}H-COSY NMR spectrum of **3k+3k'** (300.1 MHz, $CDCl_3$)

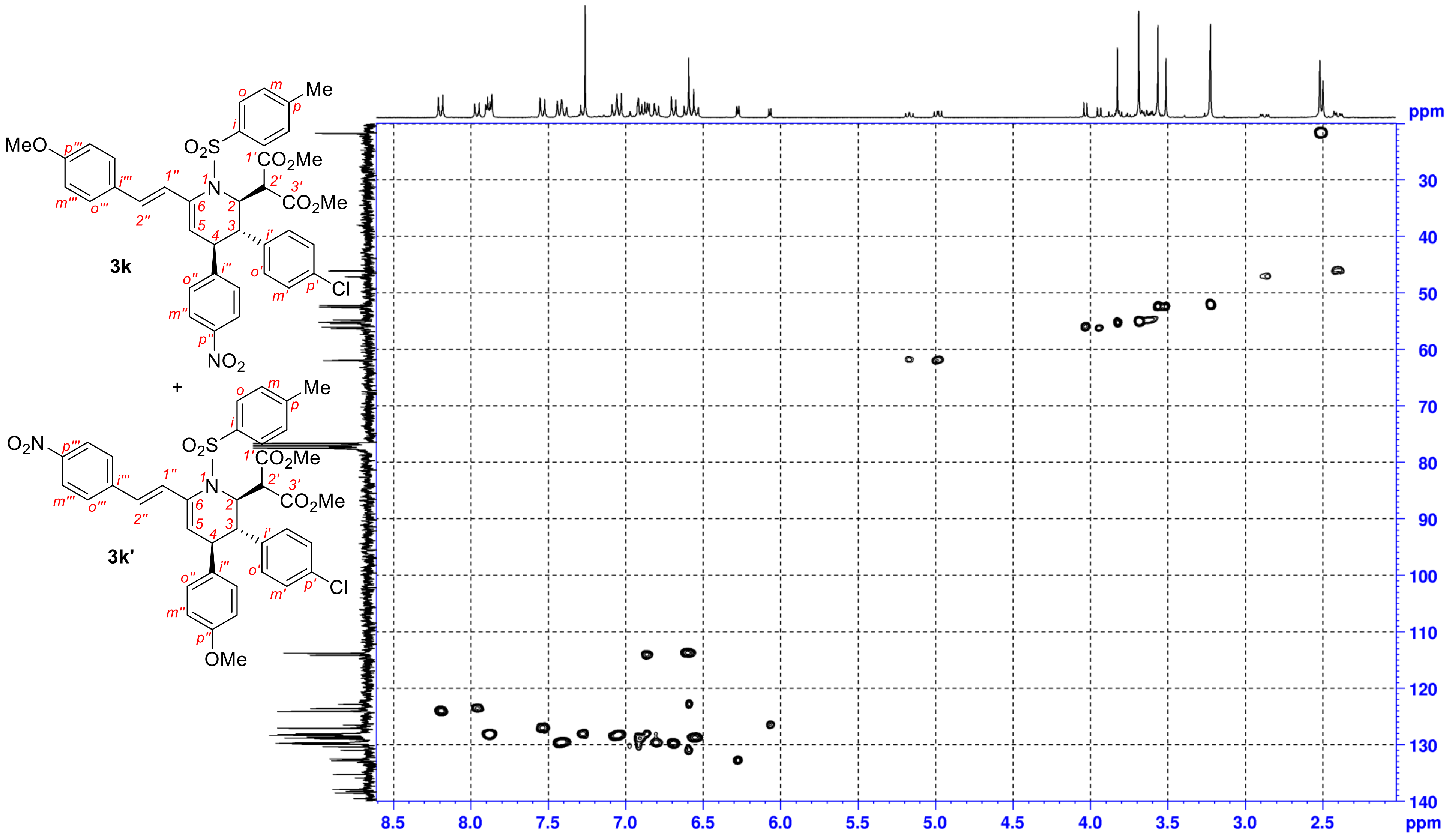

^{1}H,^{13}C-HSQC NMR spectrum of **3k+3k'** (^{1}H: 300.1 MHz; ^{13}C: 75.5 MHz; $CDCl_3$)

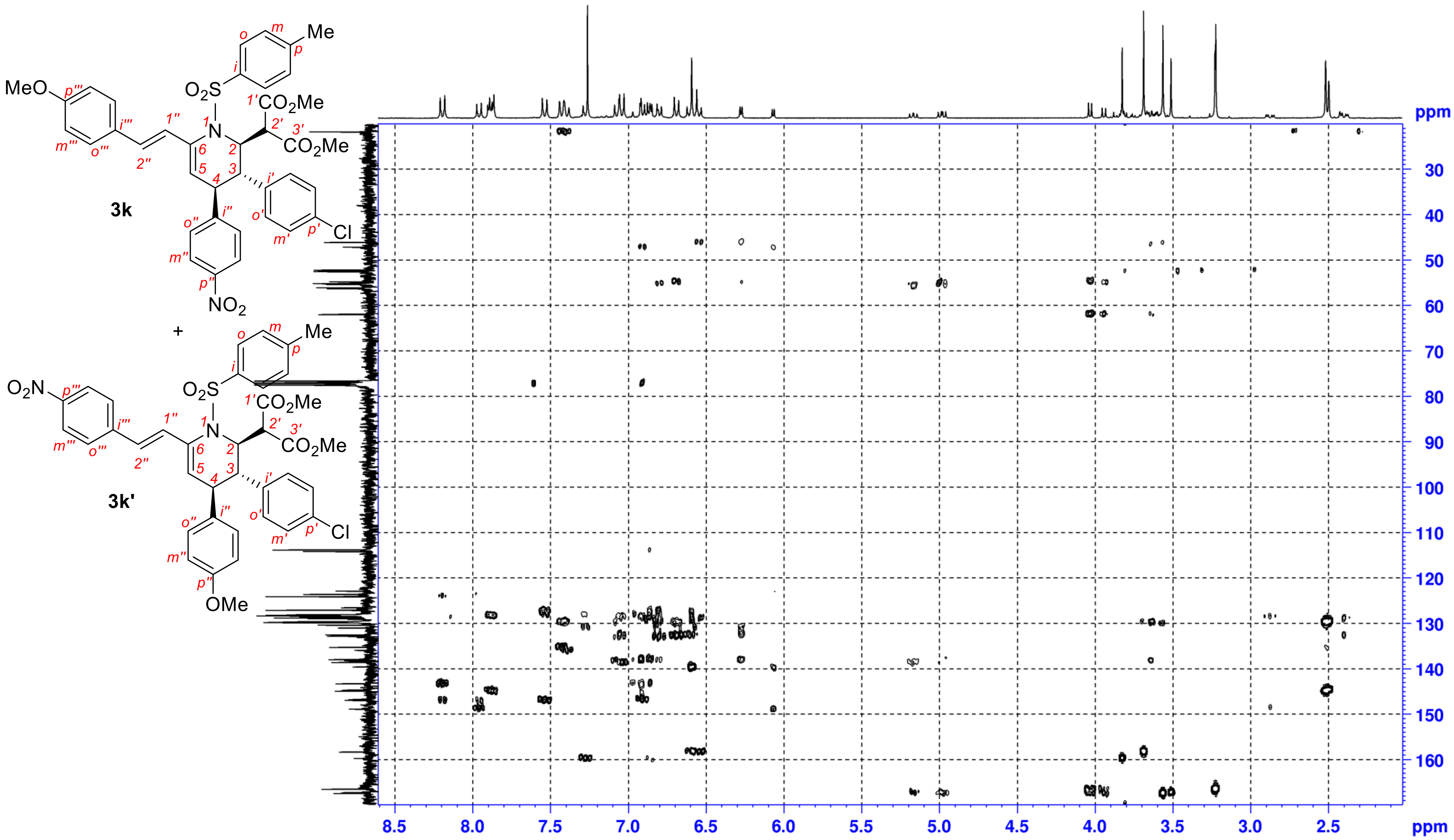

^{1}H,^{13}C-HMBC NMR spectrum of **3k+3k'** (^{1}H: 300.1 MHz; ^{13}C: 75.5 MHz; $CDCl_3$)

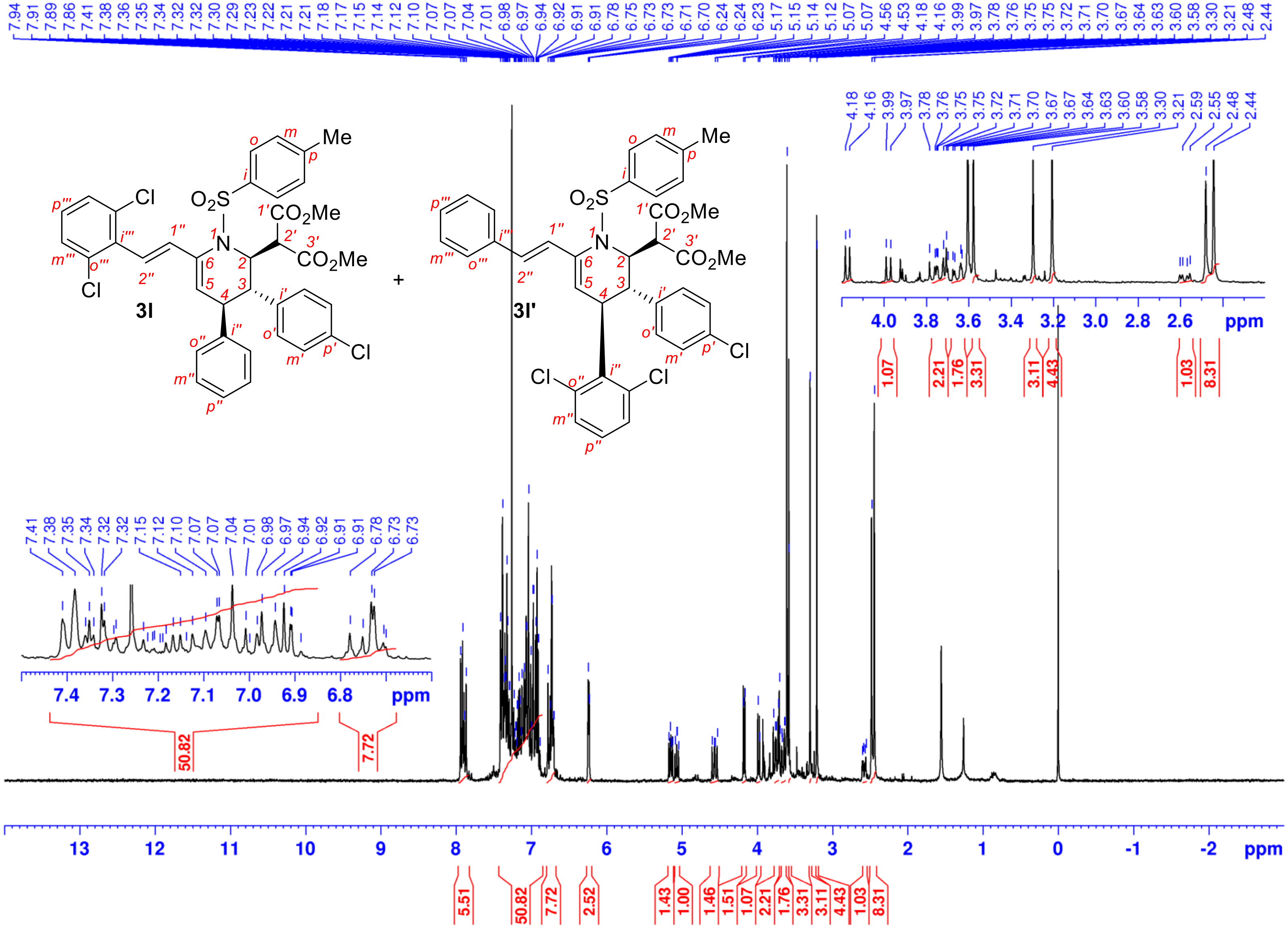

^{1}H NMR spectrum of **3l+3l'** (300.1 MHz, $CDCl_3$)

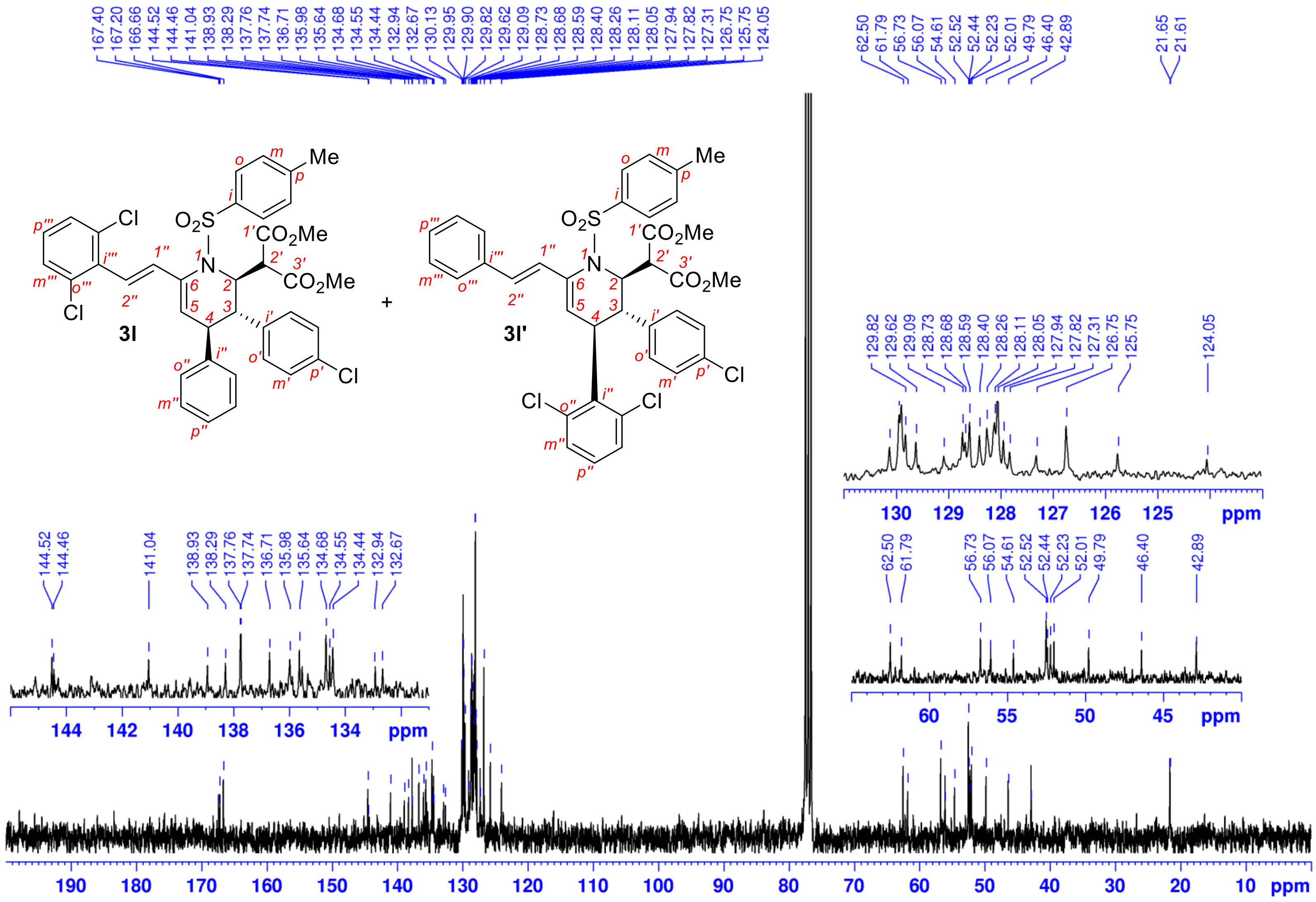

^{13}C NMR spectrum of **3l+3l’** (75.5 MHz, $CDCl_3$)

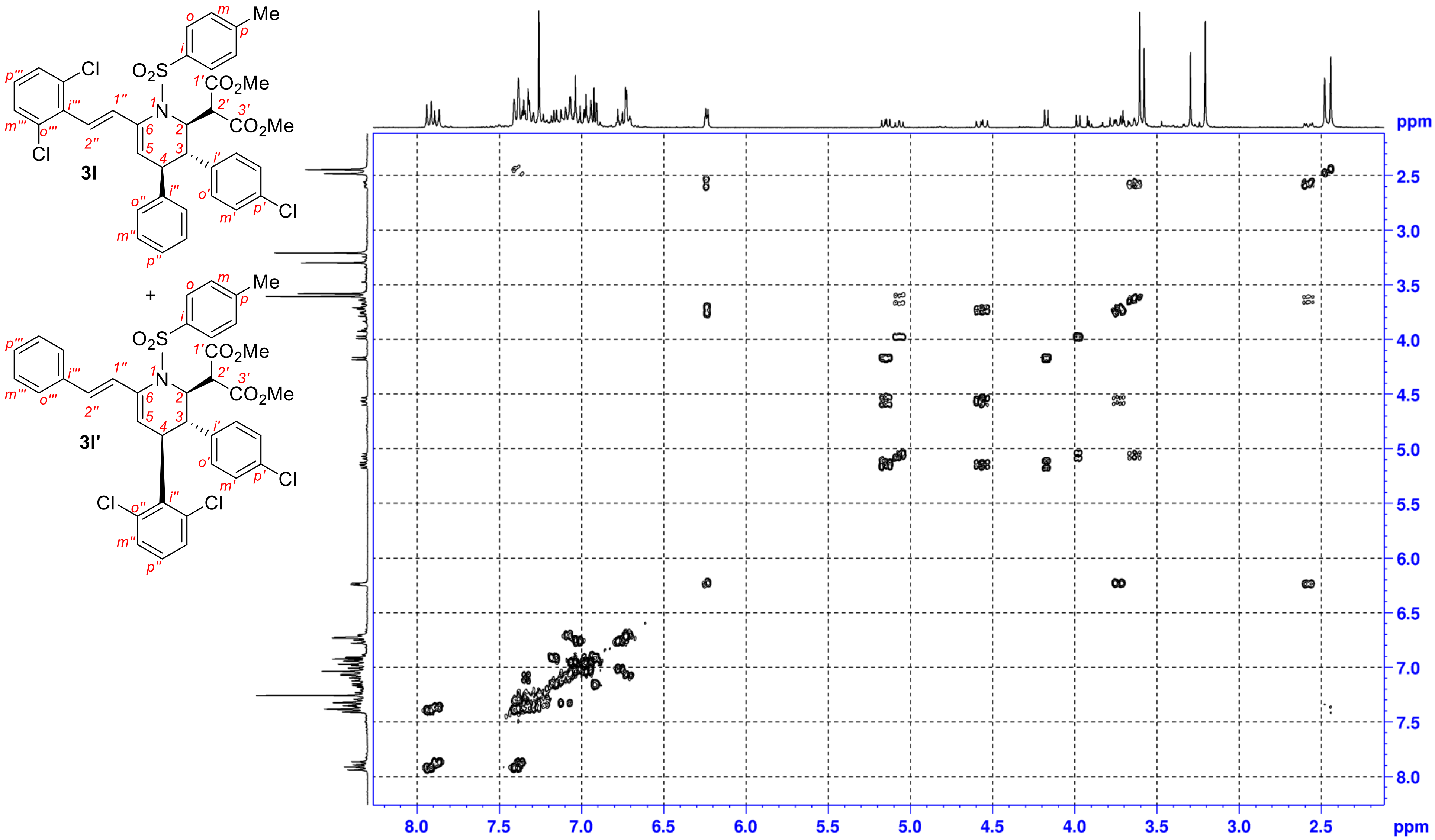

^{1}H,^{1}H-COSY NMR spectrum of **3l+3l’** (300.1 MHz, $CDCl_3$)

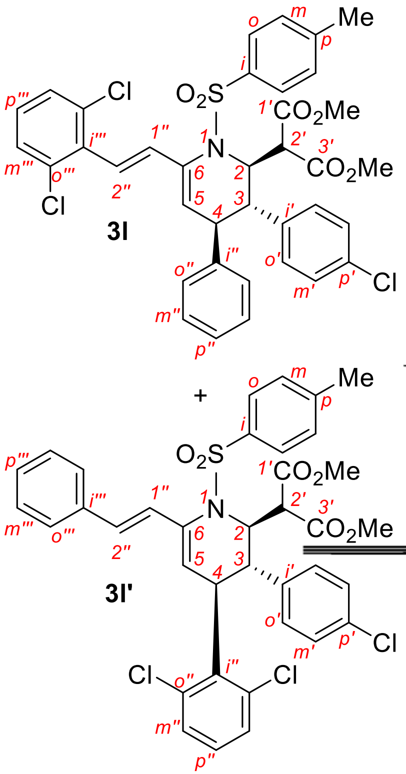

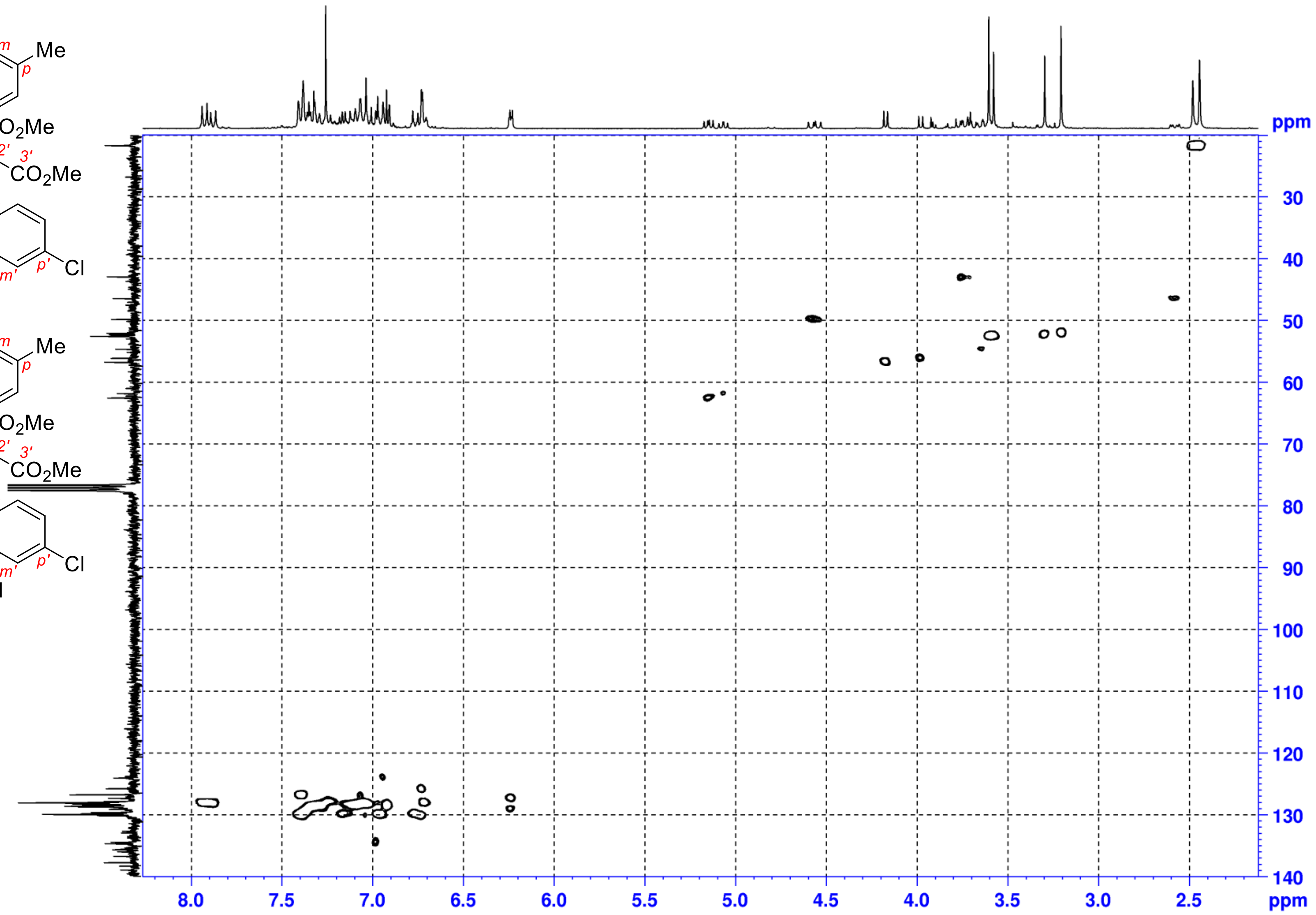

$^1H,^{13}C$-HSQC NMR spectrum of **3l+3l’** (^1H: 300.1 MHz; ^{13}C: 75.5 MHz; $CDCl_3$)

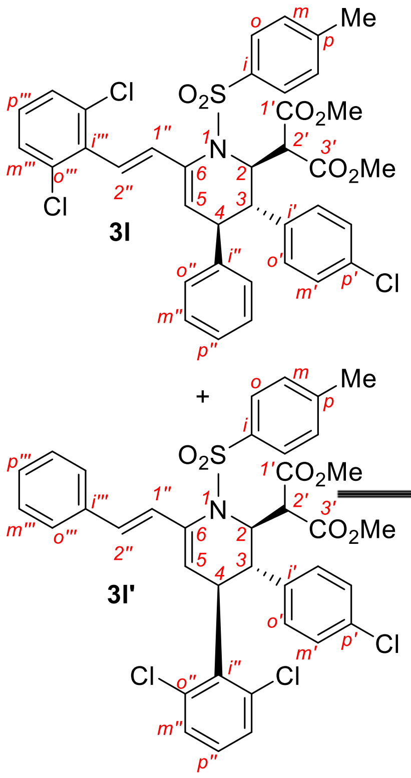

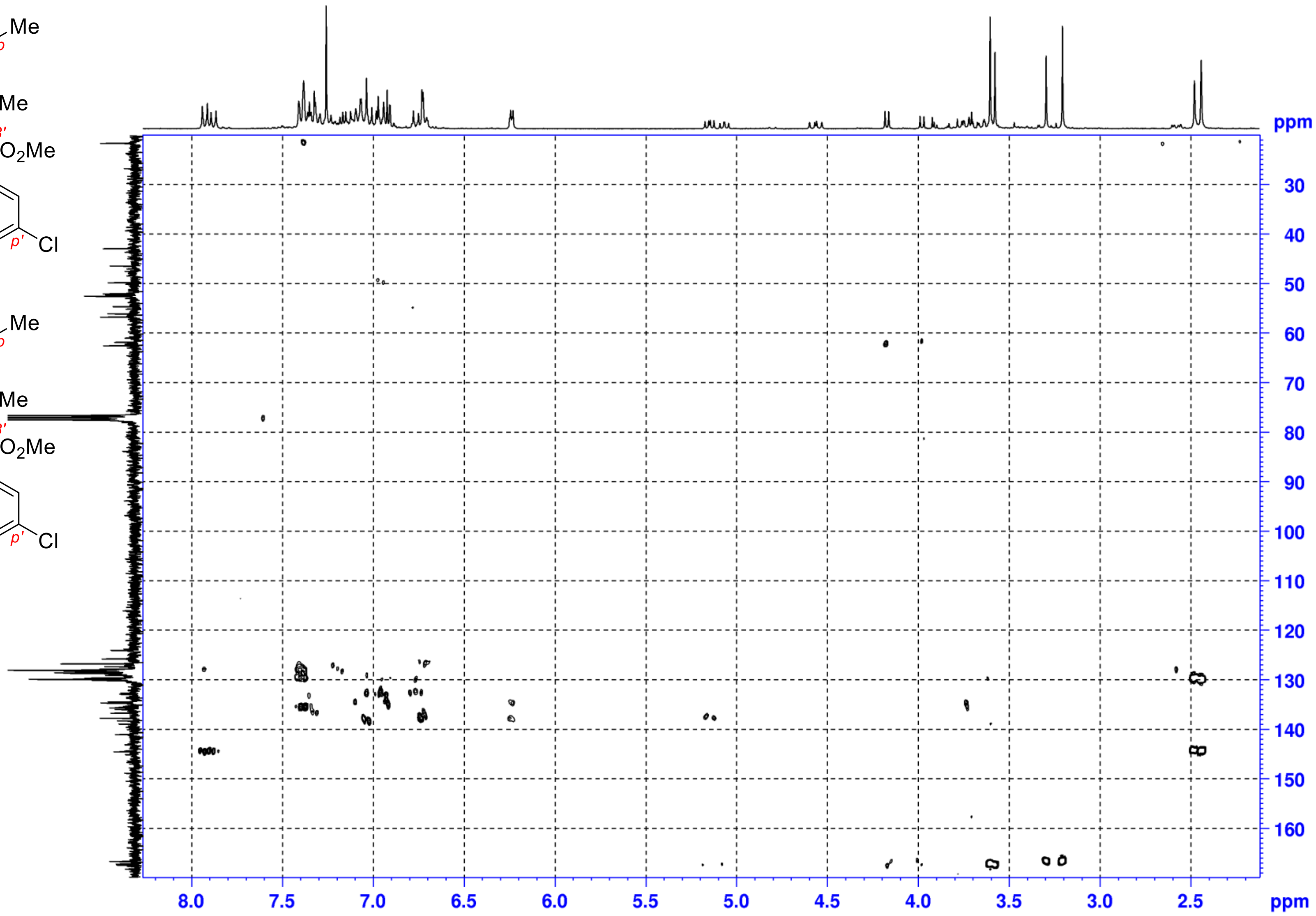

$^{1}H,^{13}C$-HMBC NMR spectrum of **3l+3l'** (^{1}H: 300.1 MHz; ^{13}C: 75.5 MHz; $CDCl_3$)

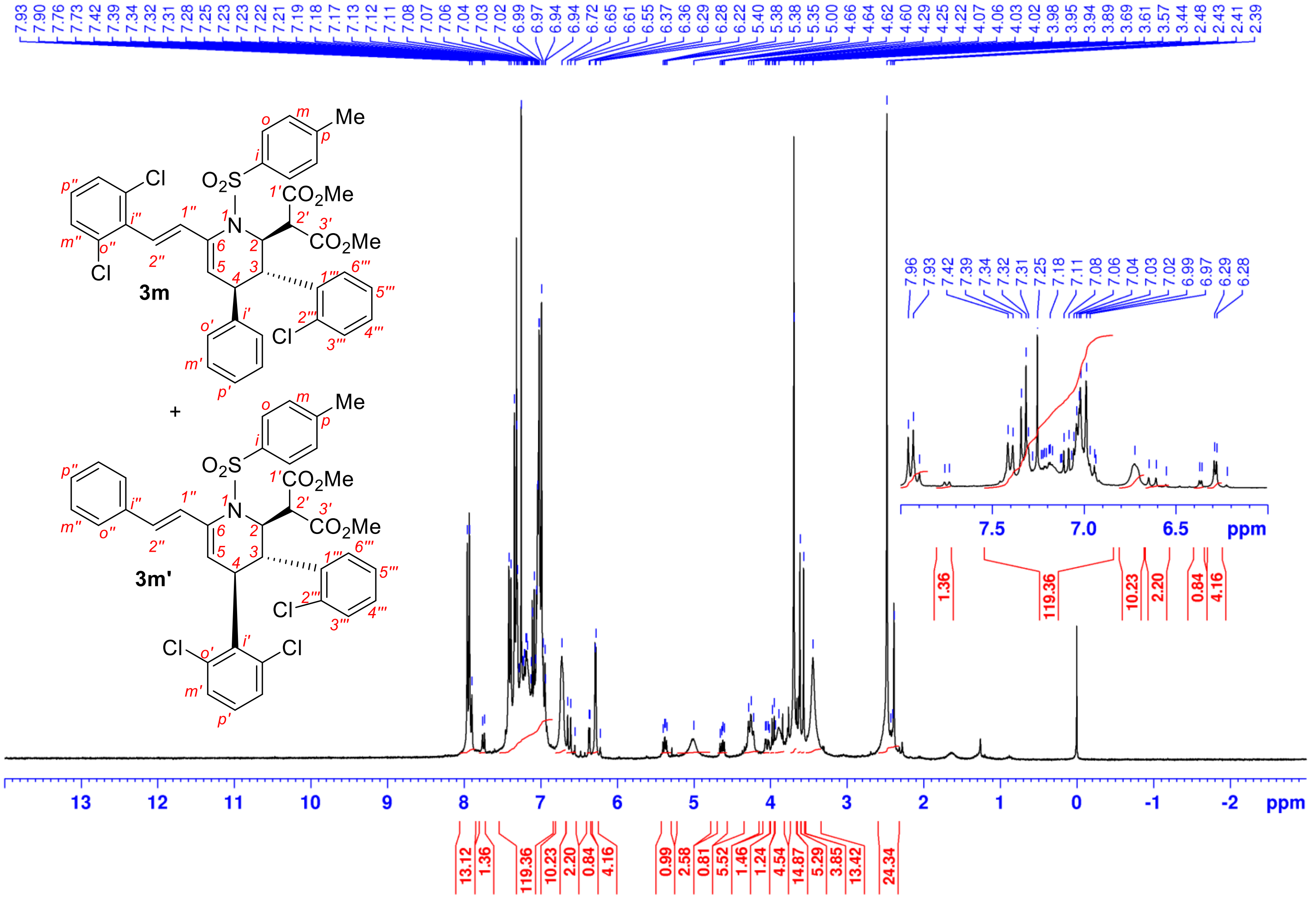

^{1}H NMR spectrum of **3m+3m'** (300.1 MHz, $CDCl_3$)

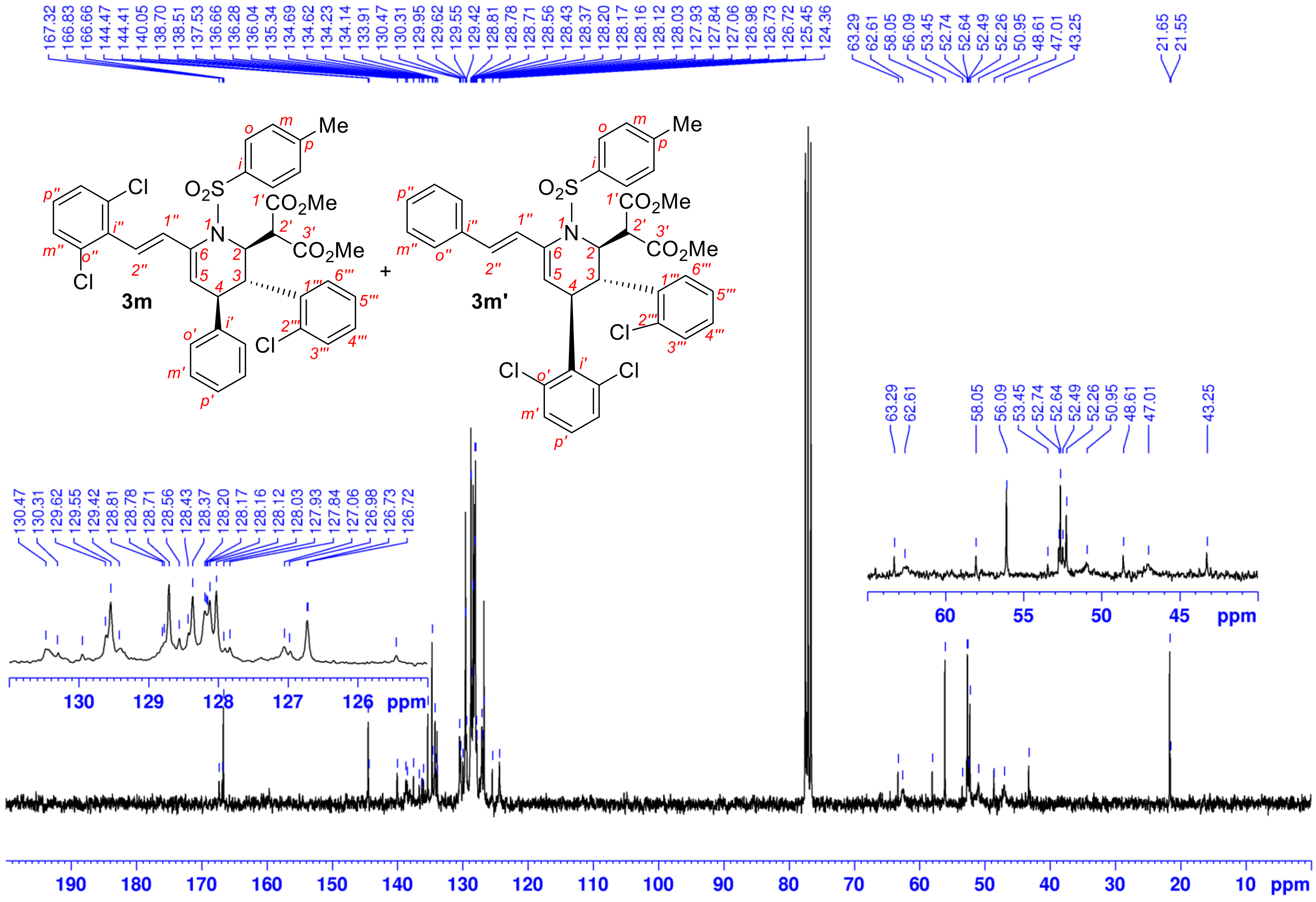

^{13}C NMR spectrum of **3m+3m'** (75.5 MHz, $CDCl_3$)

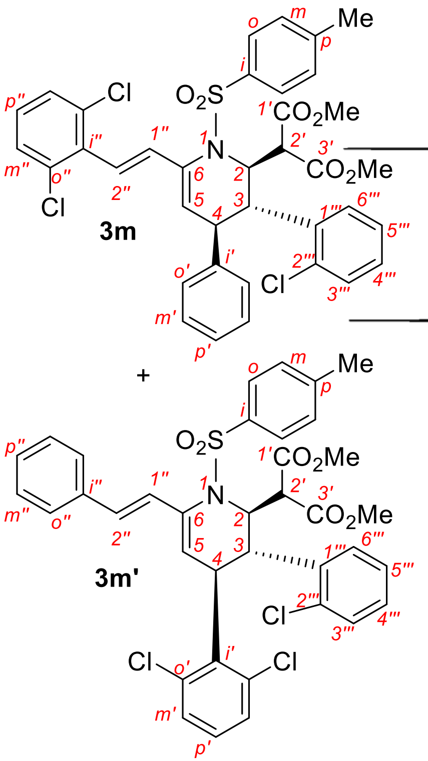

3m + **3m'**

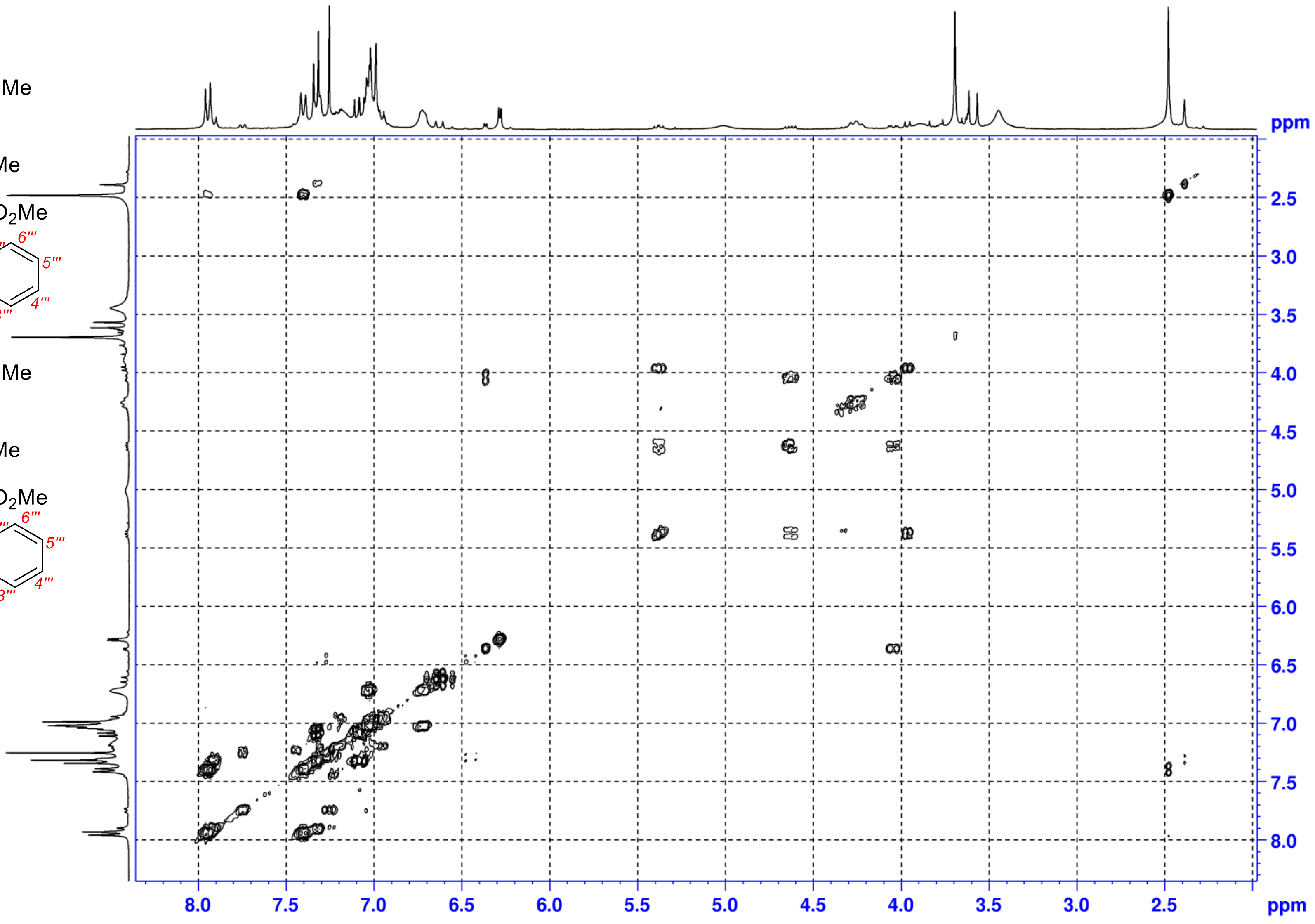

^{1}H,^{1}H-COSY NMR spectrum of **3m+3m’** (300.1 MHz, $CDCl_3$)

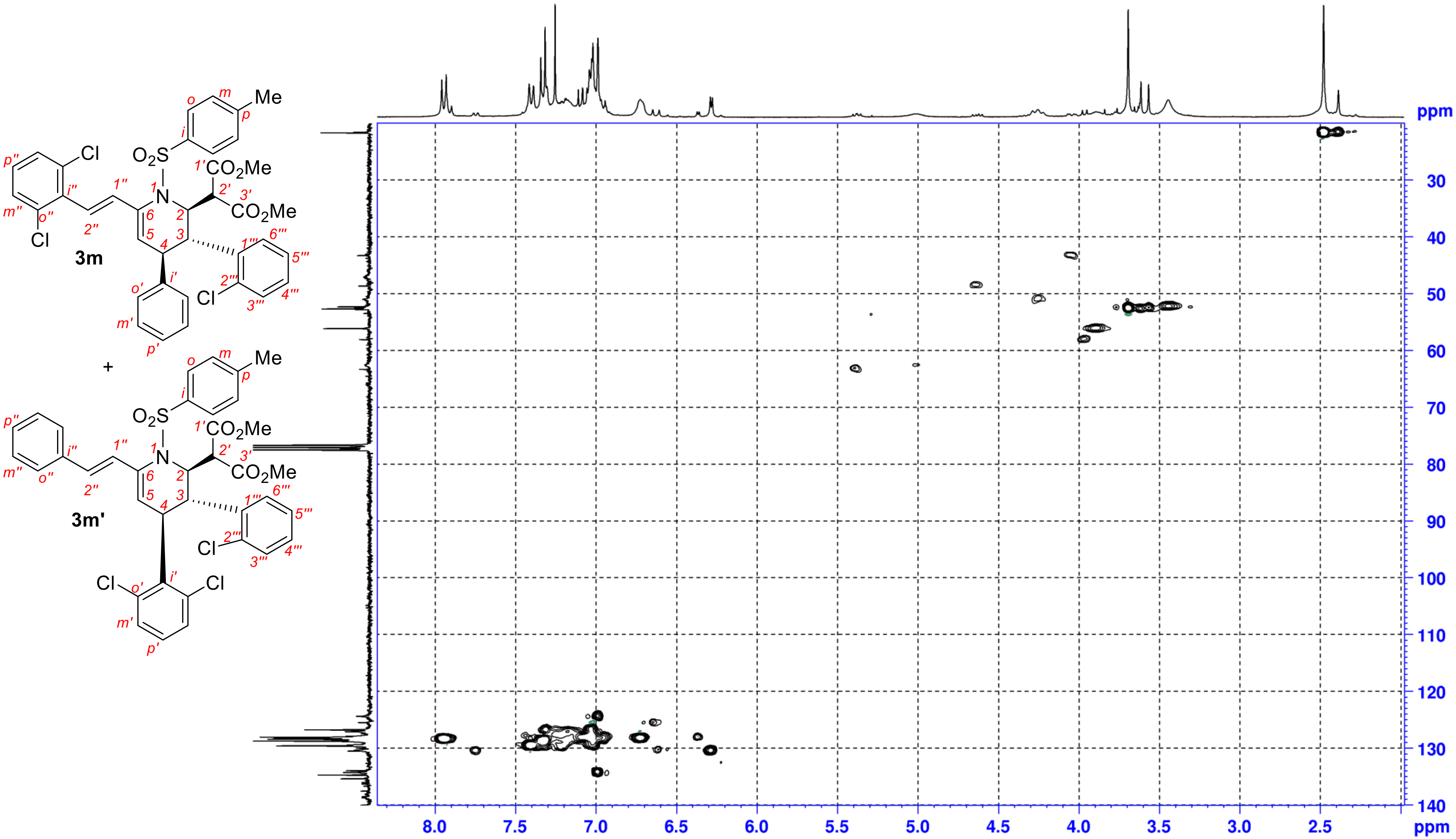

^{1}H,^{13}C-HSQC NMR spectrum of **3m+3m'** (^{1}H: 300.1 MHz; ^{13}C: 75.5 MHz; $CDCl_3$)

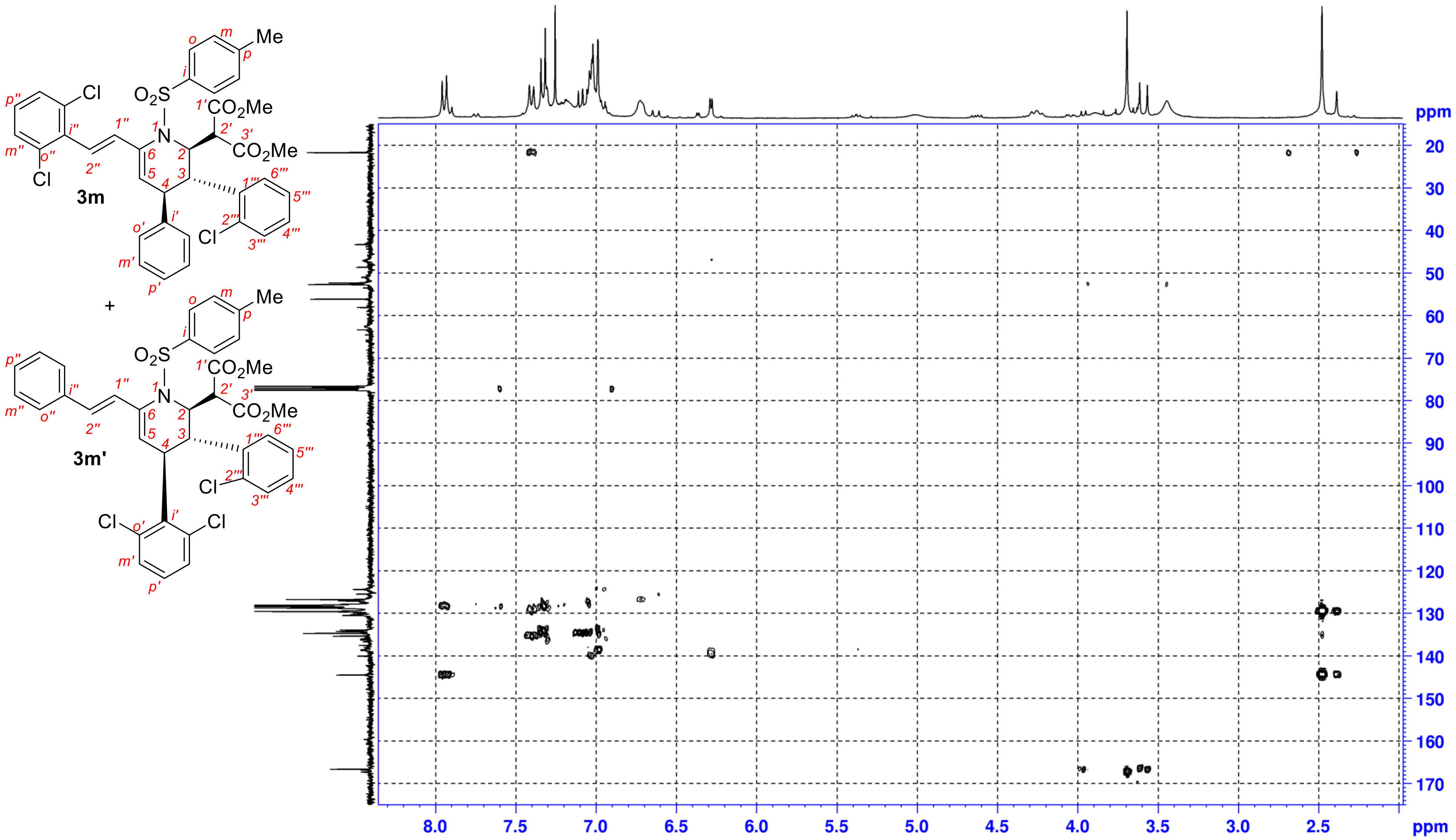

$^{1}H,^{13}C$-HMBC NMR spectrum of **3m+3m'** (^{1}H: 300.1 MHz; ^{13}C: 75.5 MHz; $CDCl_3$)

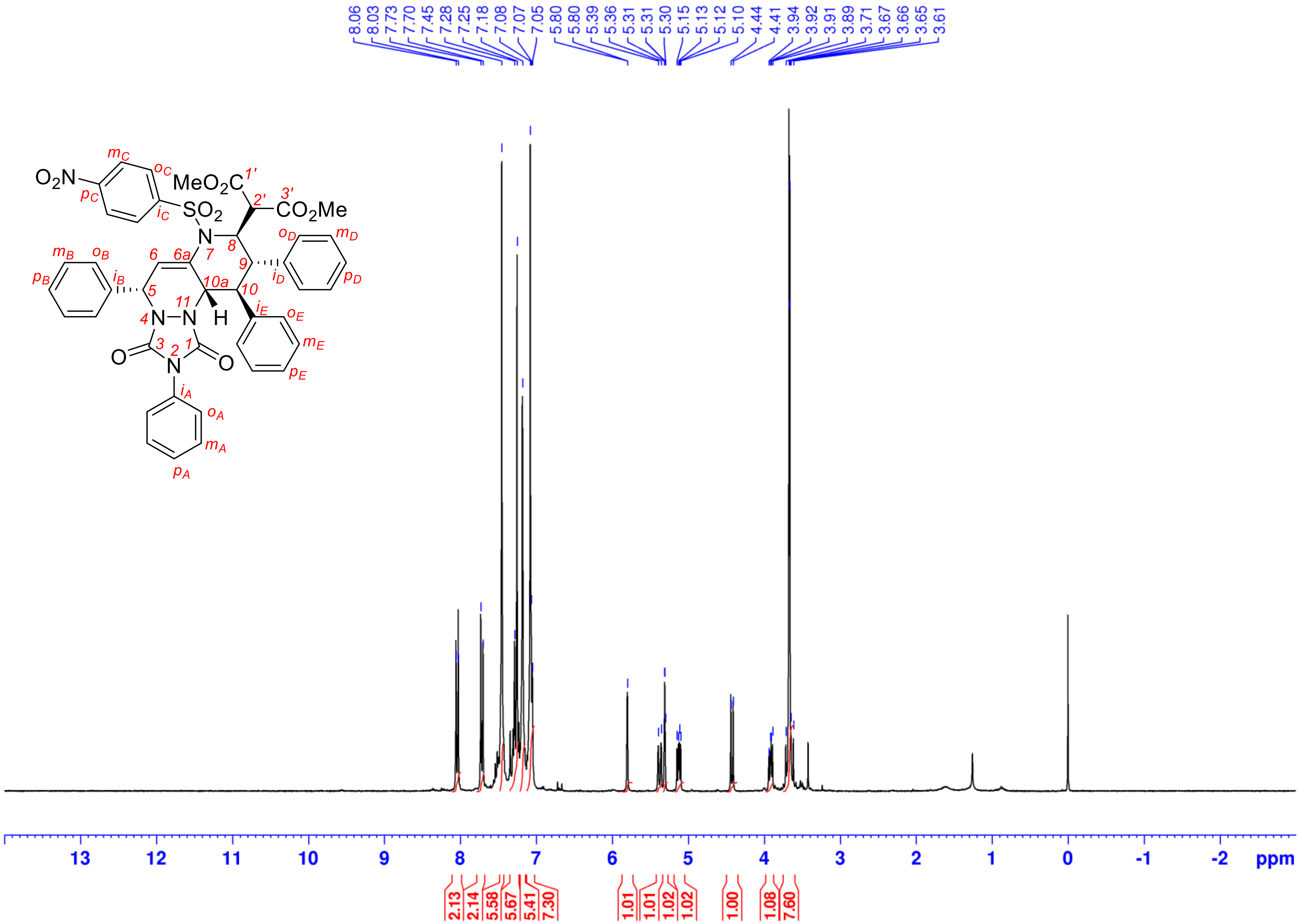

^{1}H NMR spectrum of **5a** (300.1 MHz, $CDCl_3$)

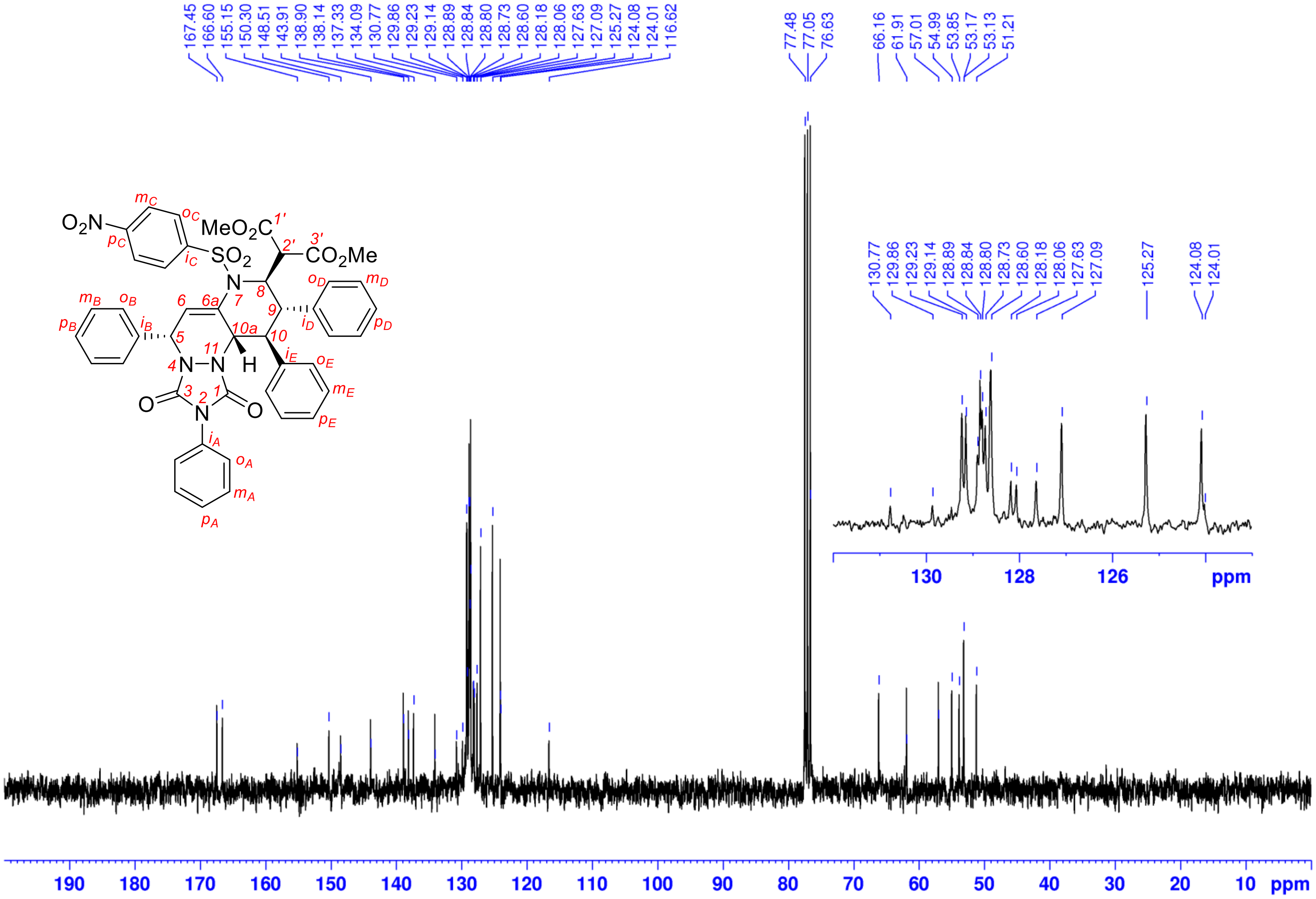

^{13}C NMR spectrum of **5a** (75.5 MHz, $CDCl_3$)

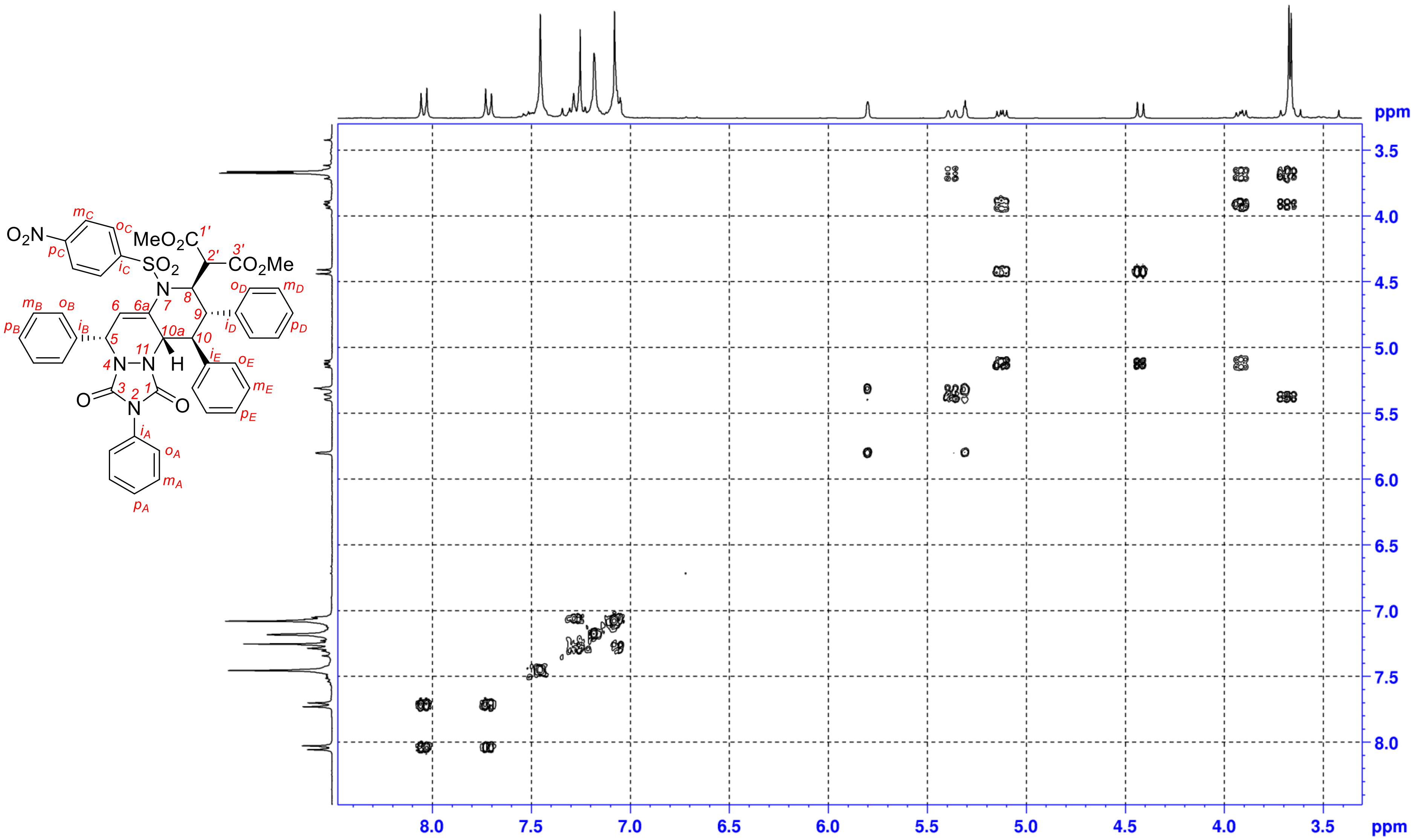

^{1}H,^{1}H-COSY NMR spectrum of **5a** (300.1 MHz, $CDCl_3$)

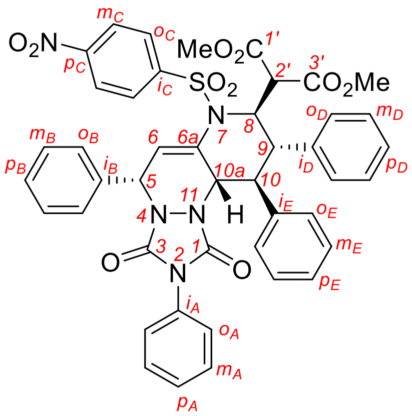

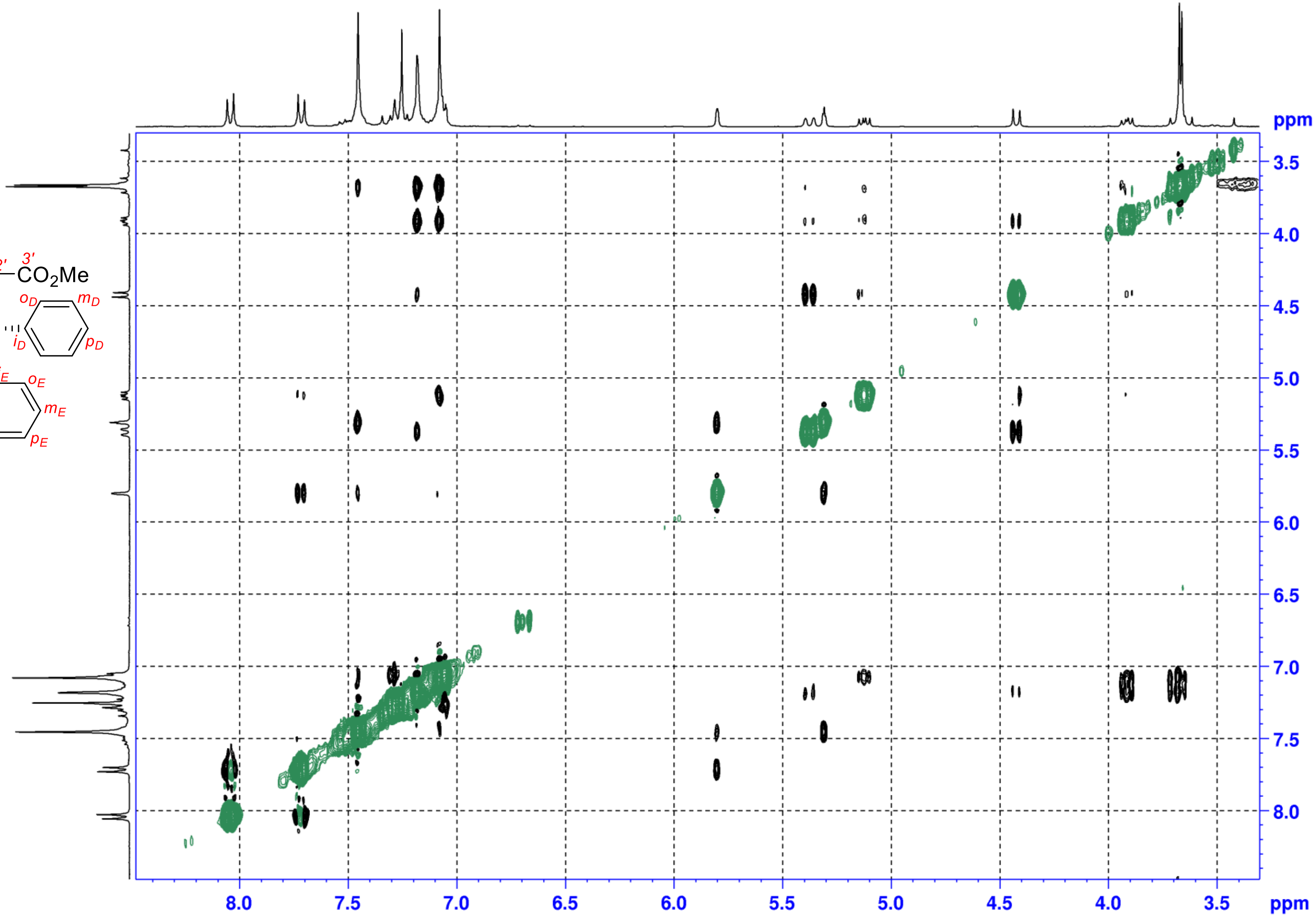

$^1H,^1H$-NOESY NMR spectrum of **5a** (300.1 MHz, $CDCl_3$)

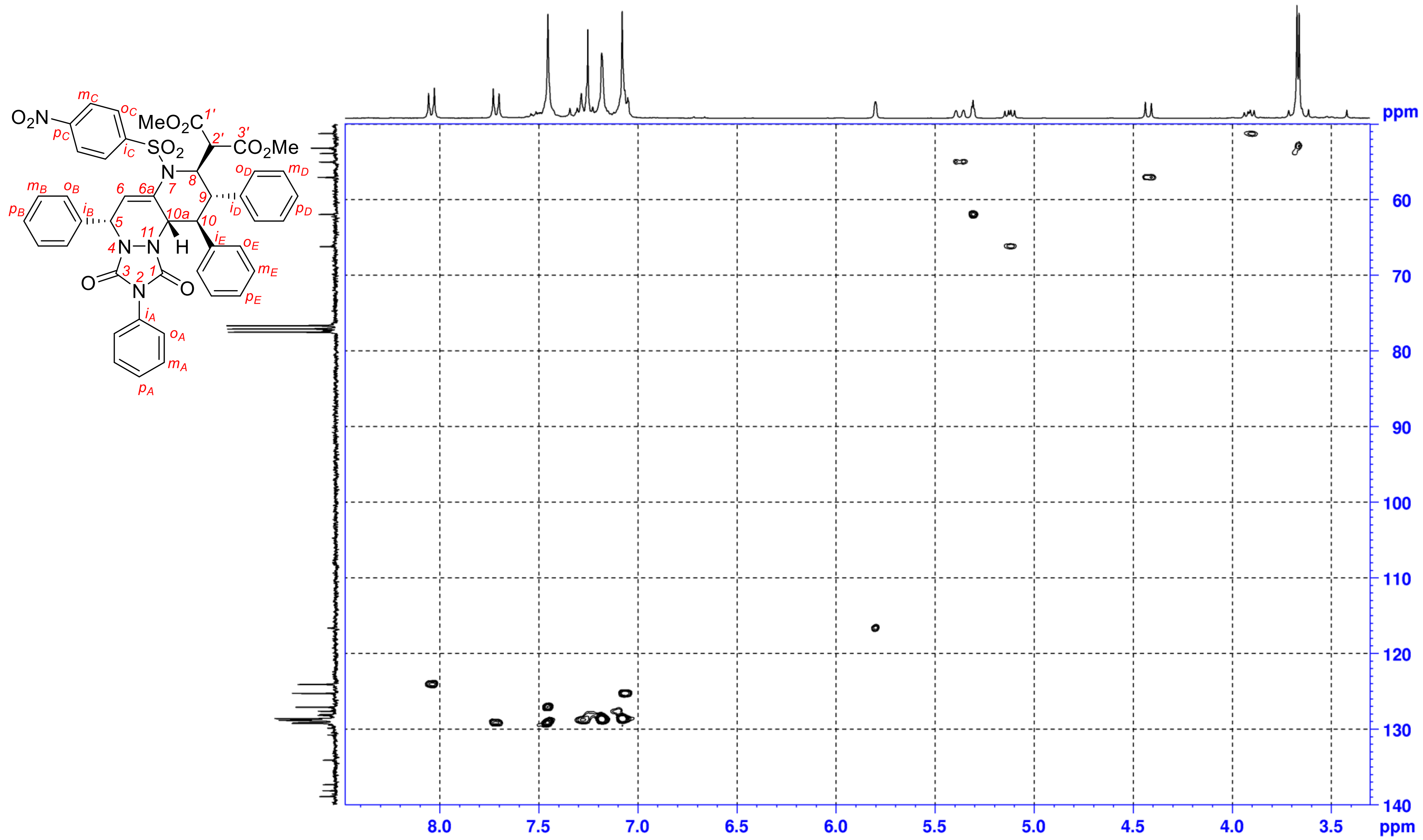

^{1}H,^{13}C-HSQC NMR spectrum of **5a** (^{1}H: 300.1 MHz; ^{13}C: 75.5 MHz; $CDCl_3$)

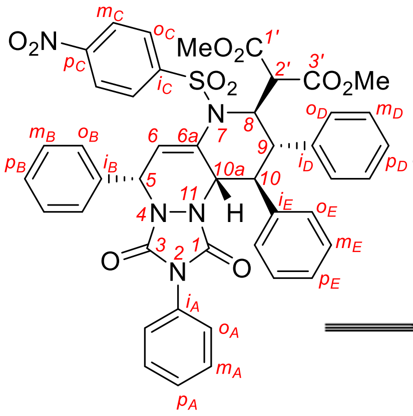

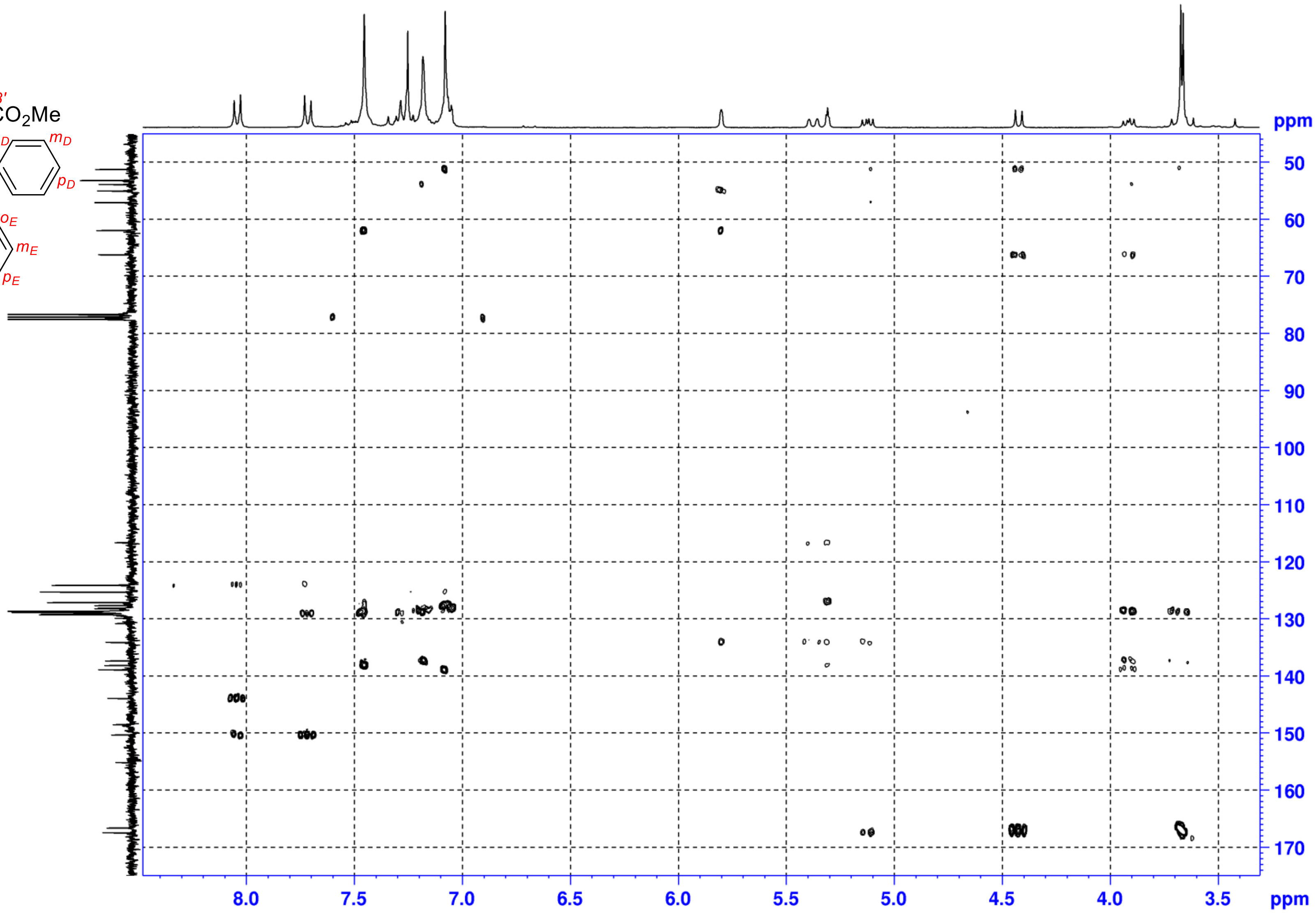

$^{1}H,^{13}C$-HMBC NMR spectrum of **5a** (^{1}H: 300.1 MHz; ^{13}C: 75.5 MHz; $CDCl_3$)

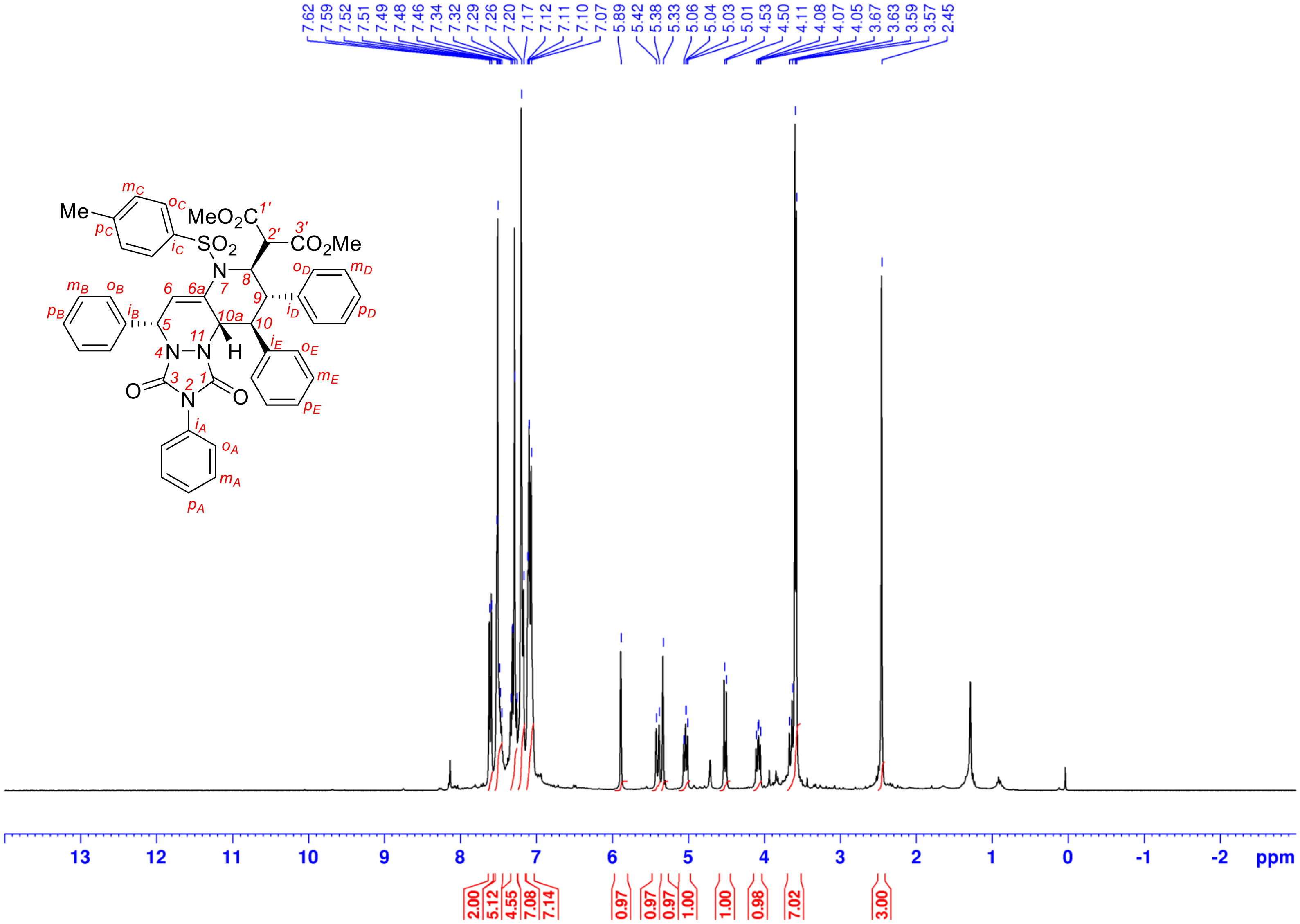

^{1}H NMR spectrum of **5b** (300.1 MHz, $CDCl_3$)

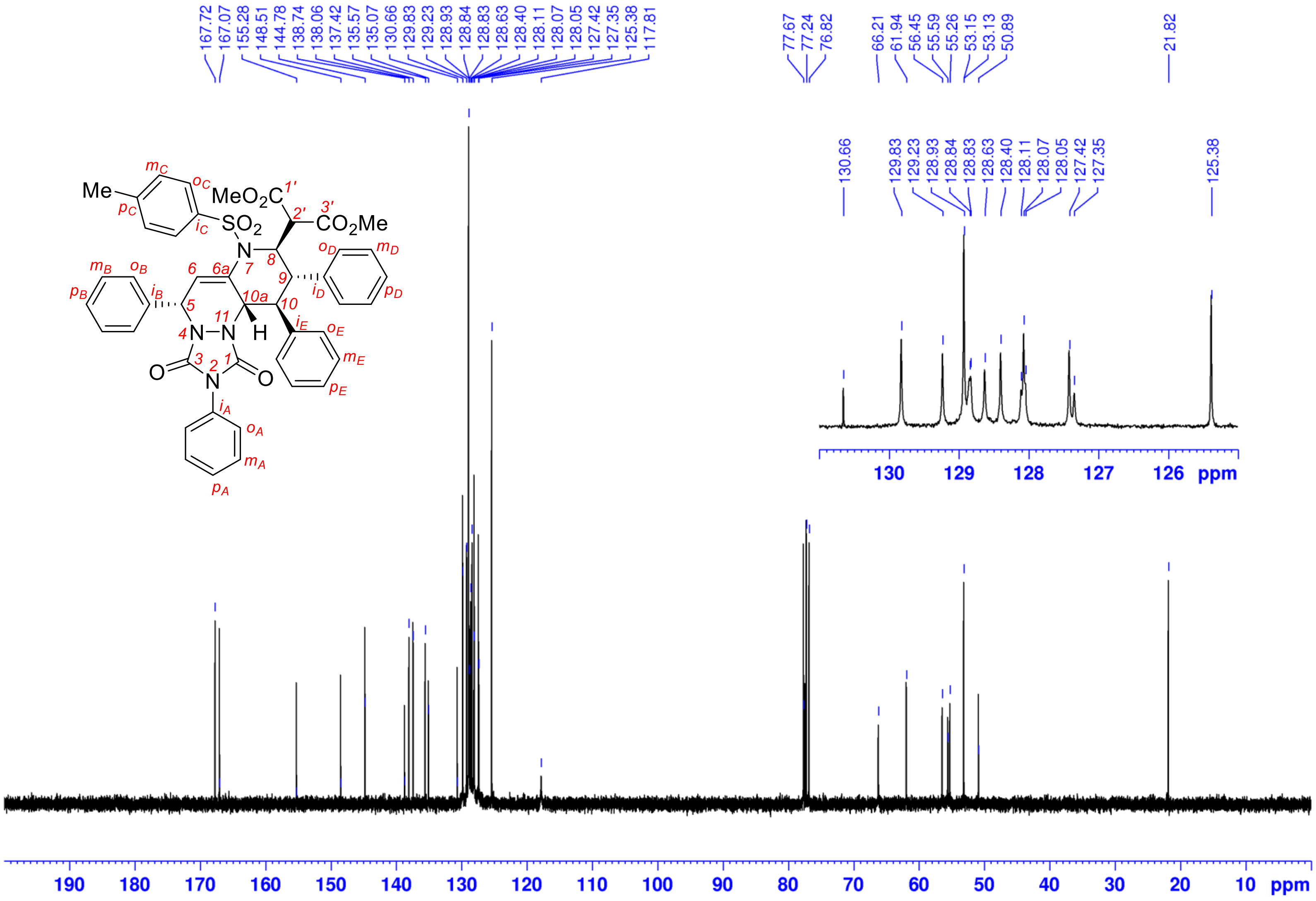

^{13}C NMR spectrum of **5b** (75.5 MHz, $CDCl_3$)

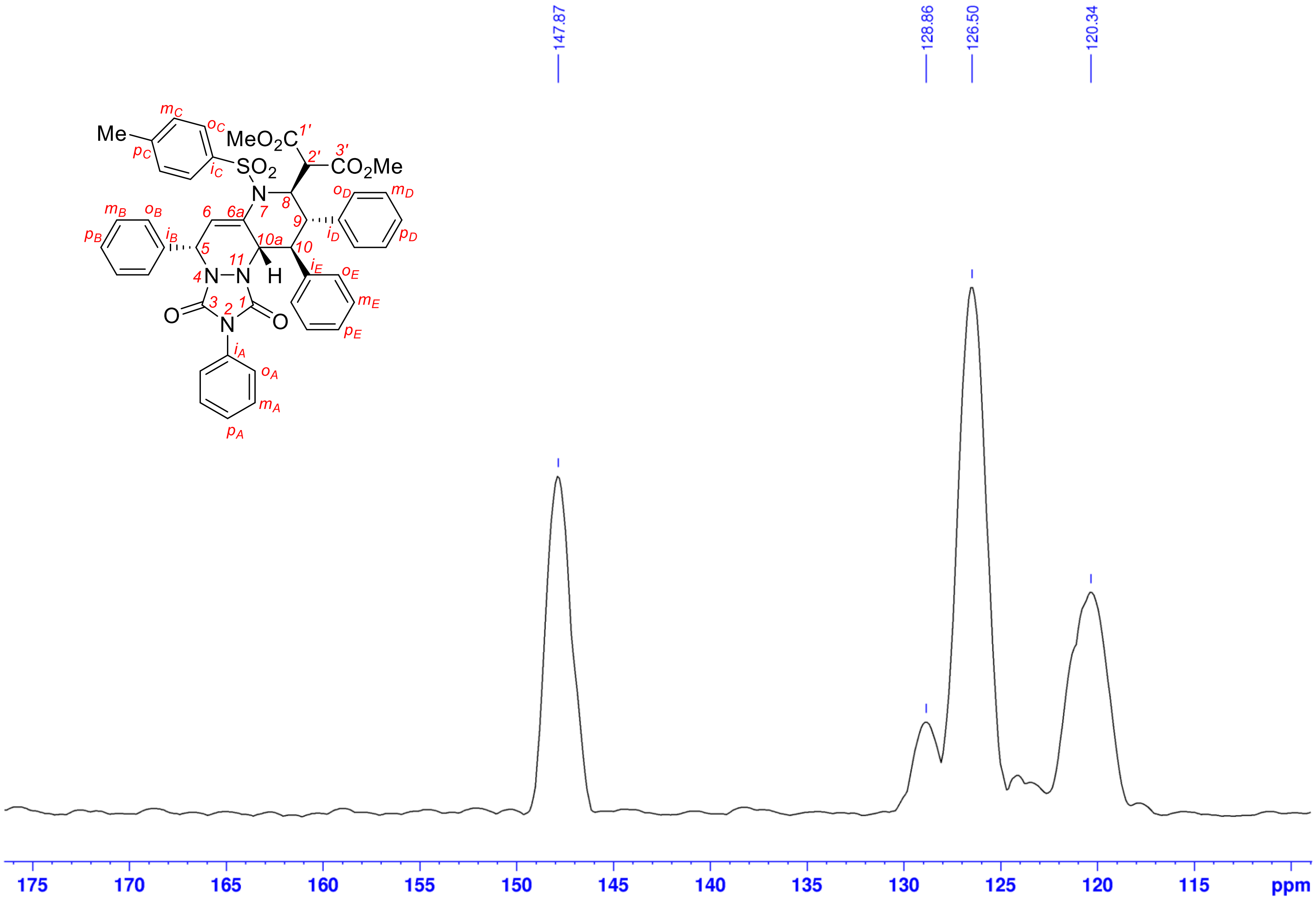

^{15}N NMR spectrum of **5b** (30.4 MHz, $CDCl_3$)

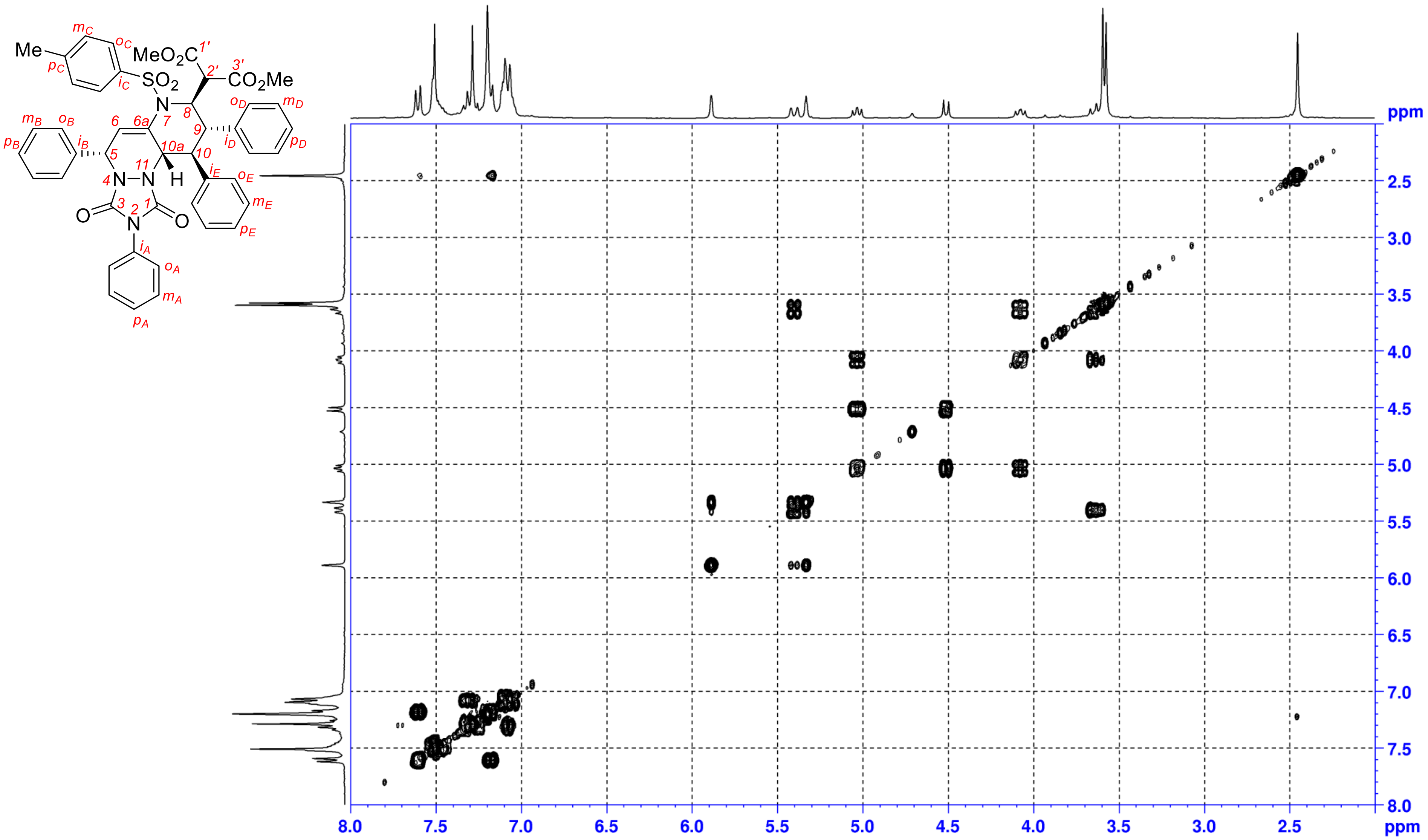

$^{1}H,^{1}H$-COSY NMR spectrum of **5b** (300.1 MHz, $CDCl_3$)

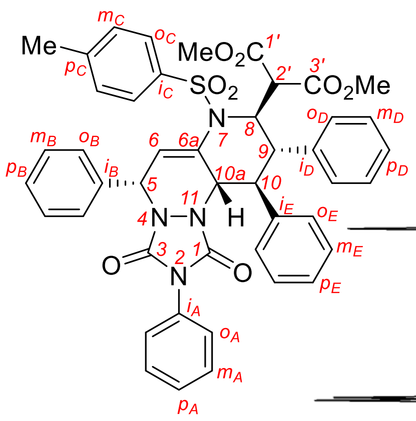

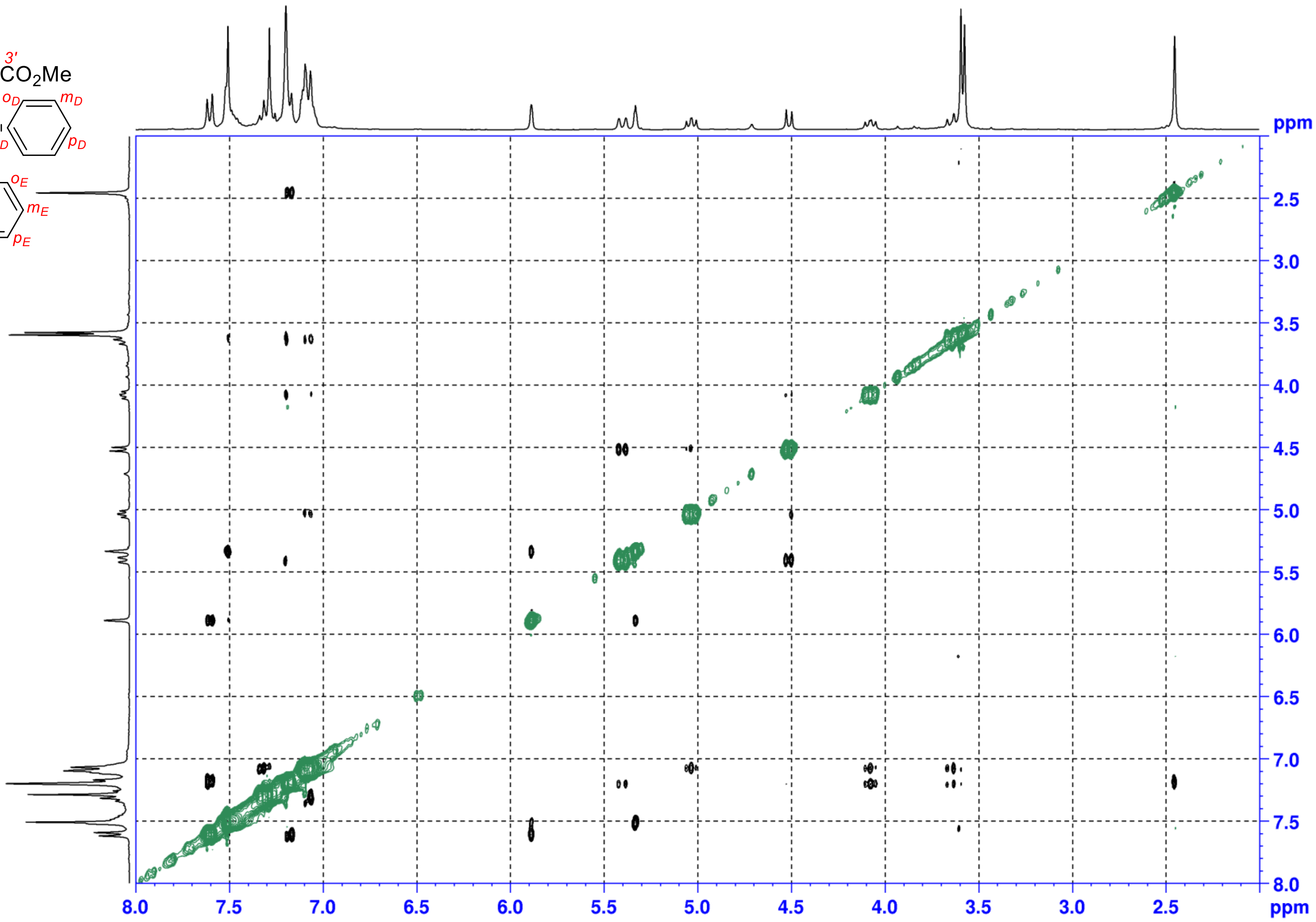

$^{1}H,^{1}H$-NOESY NMR spectrum of **5b** (300.1 MHz, $CDCl_3$)

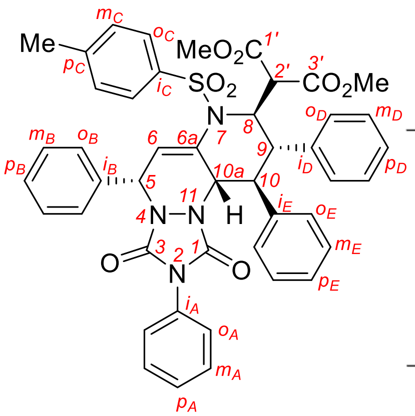

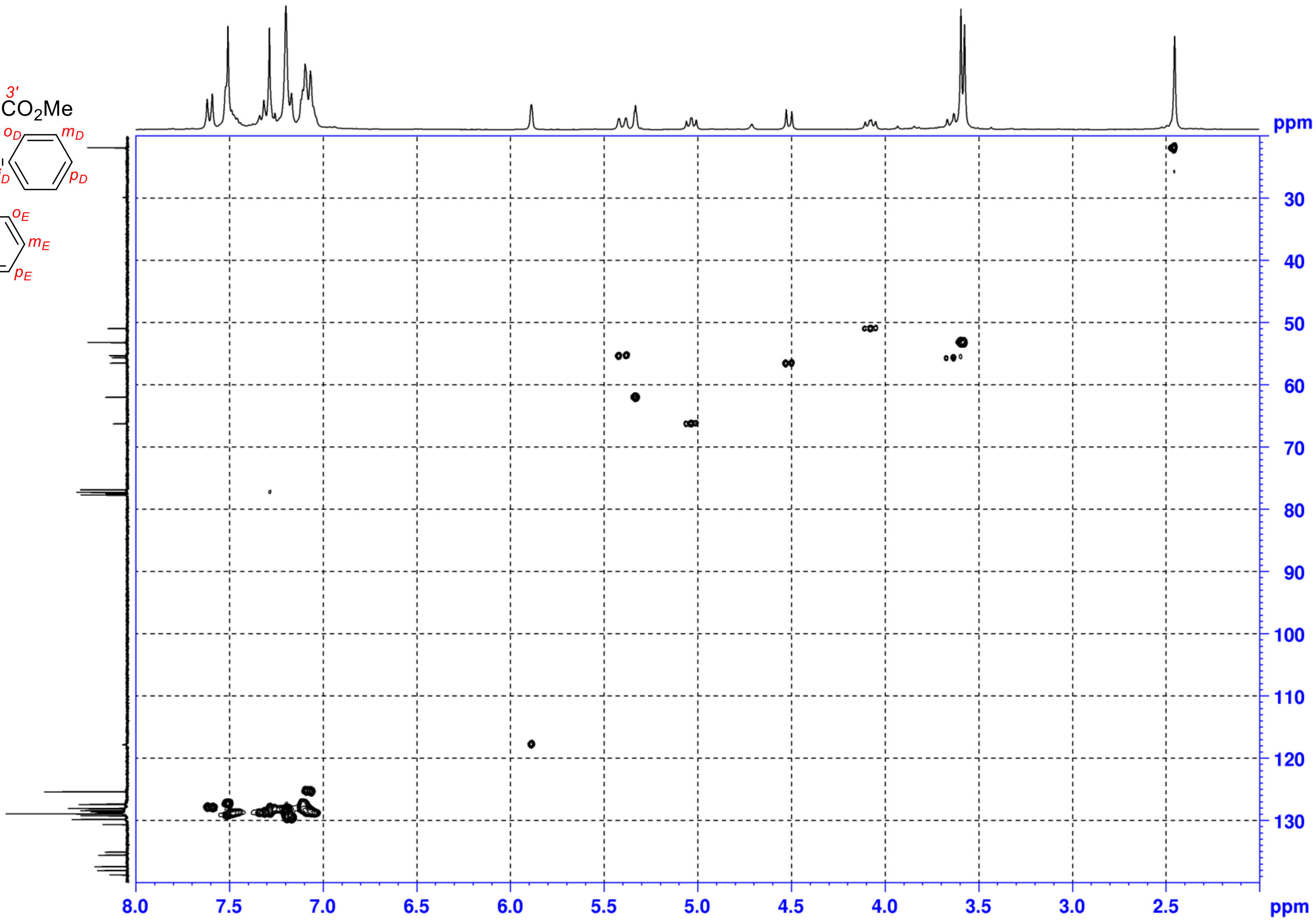

$^{1}H,^{13}C$-HSQC NMR spectrum of **5b** (^{1}H: 300.1 MHz; ^{13}C: 75.5 MHz; $CDCl_3$)

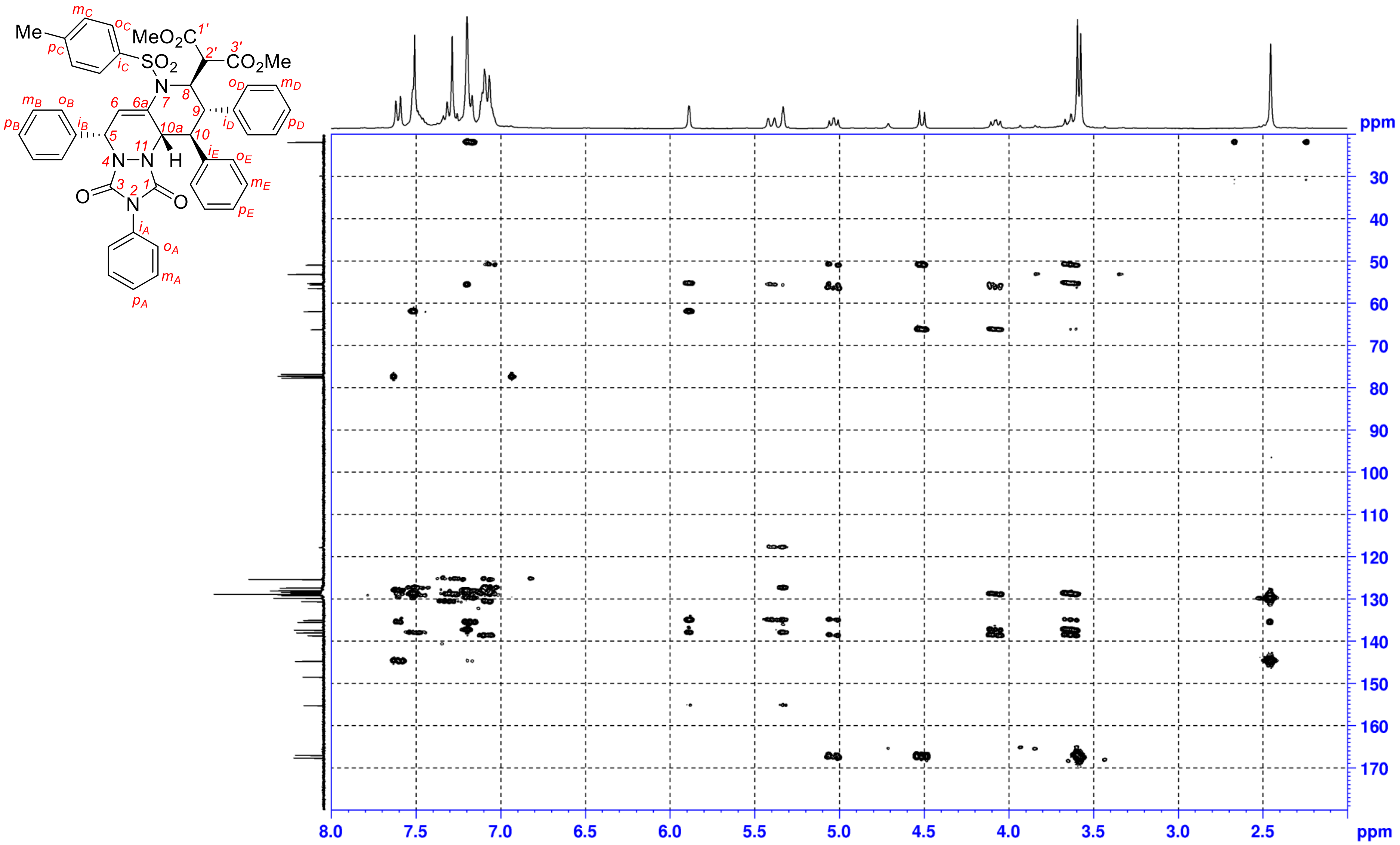

^{1}H,^{13}C-HMBC NMR spectrum of **5b** (^{1}H: 300.1 MHz; ^{13}C: 75.5 MHz; $CDCl_3$)

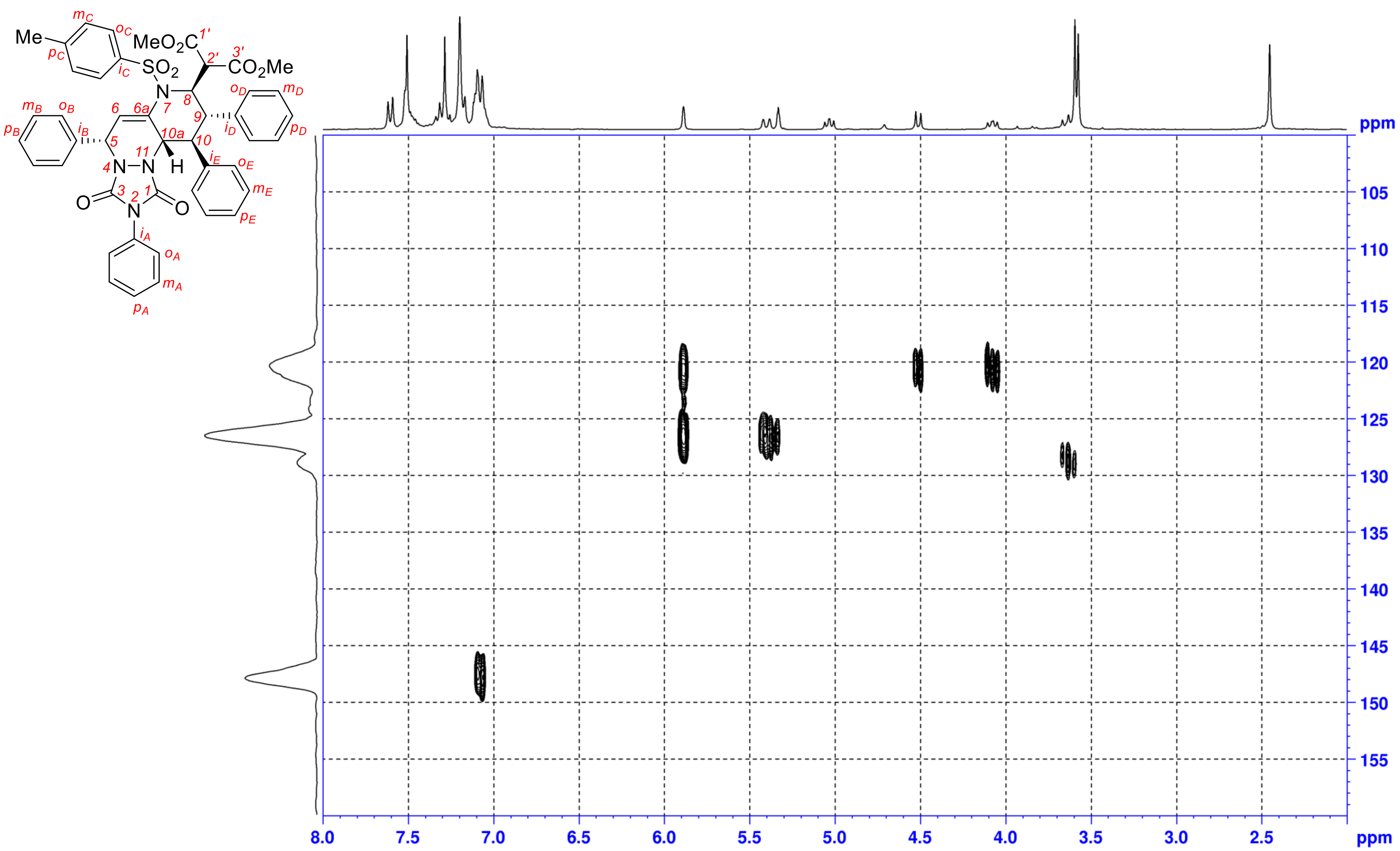

$^{1}H,^{15}N$-HMBC NMR spectrum of **5b** (^{1}H: 300.1 MHz; ^{15}N: 30.4 MHz; $CDCl_3$)

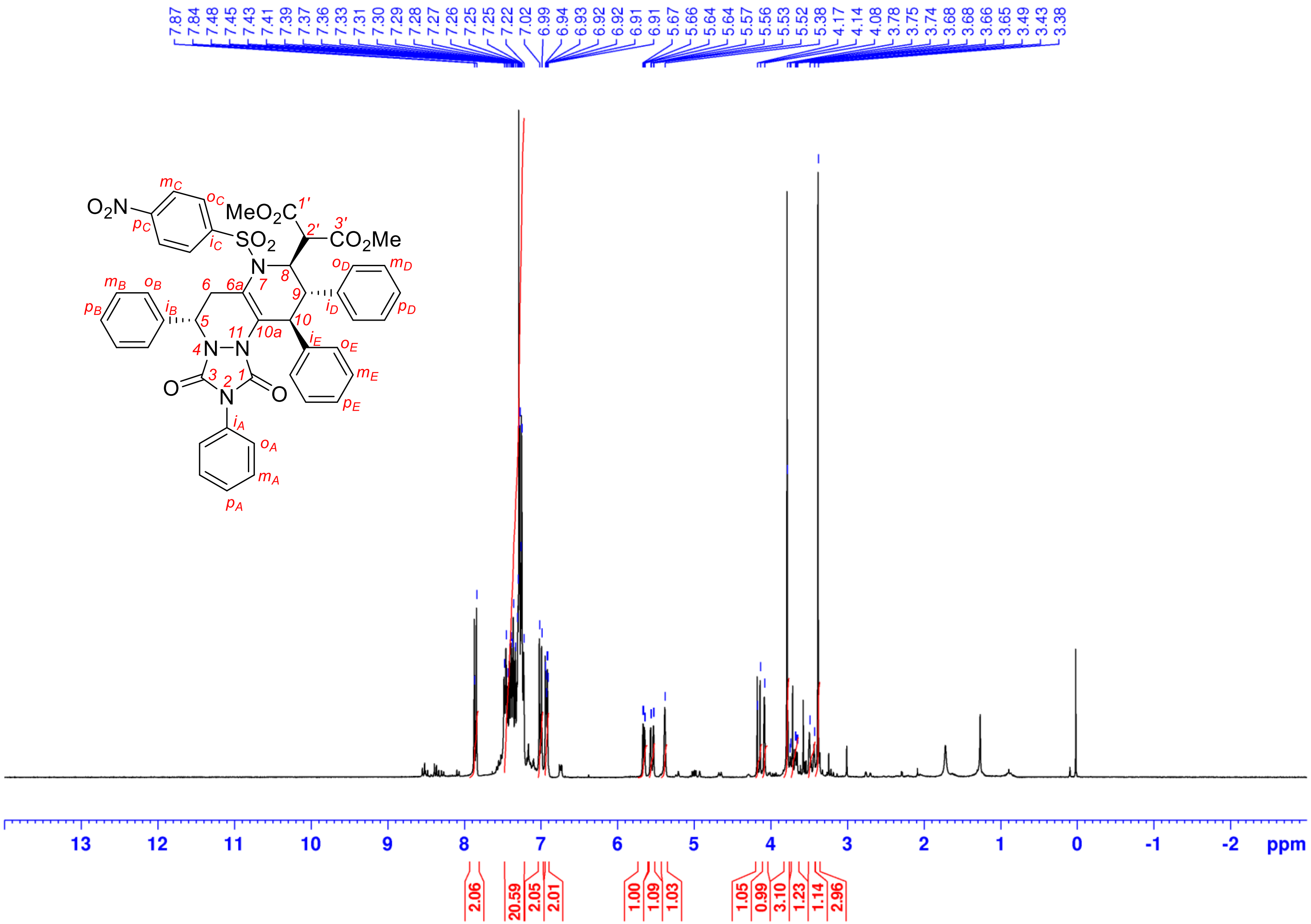

^{1}H NMR spectrum of **6** (300.1 MHz, $CDCl_3$)

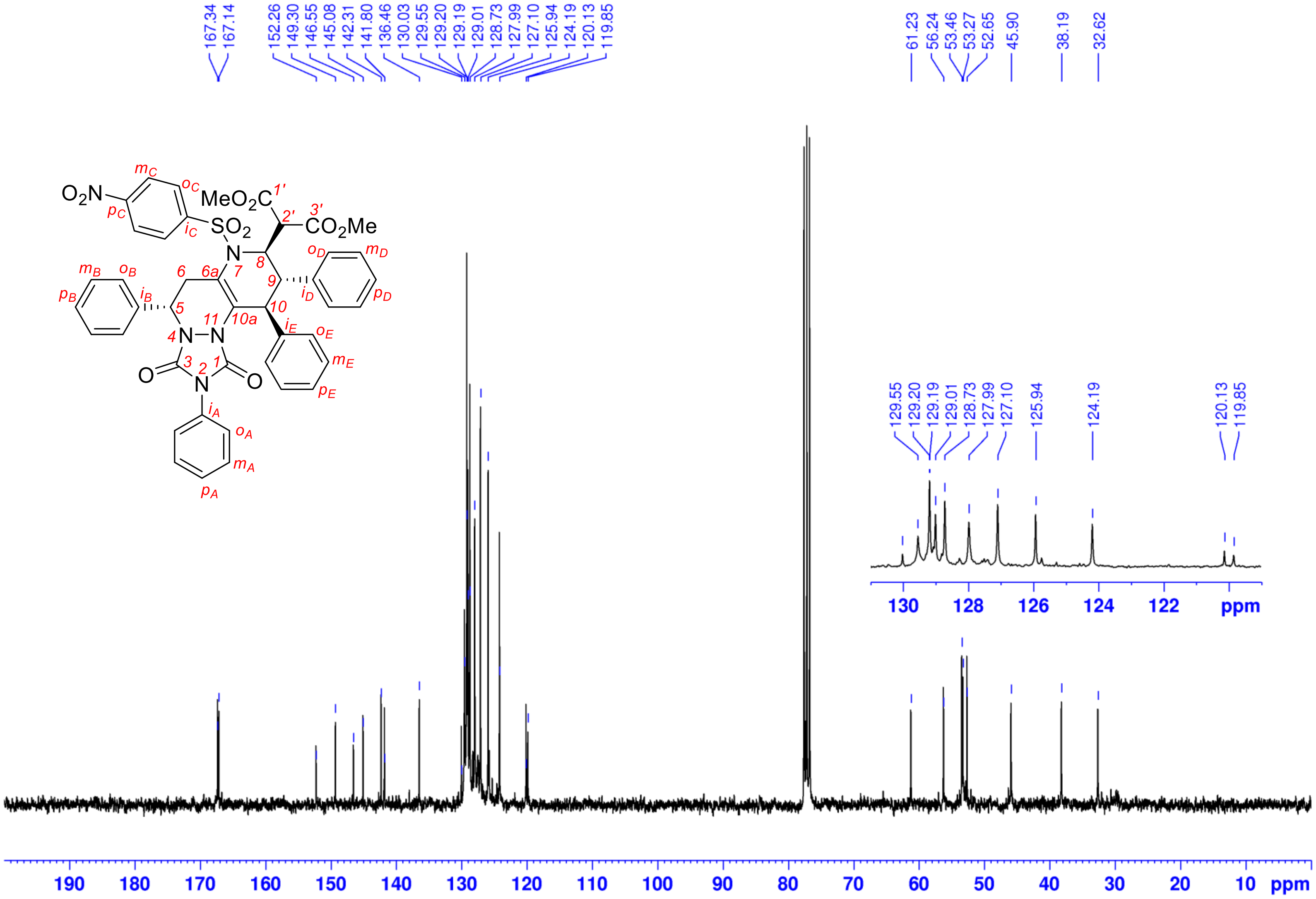

^{13}C NMR spectrum of **6** (75.5 MHz, $CDCl_3$)

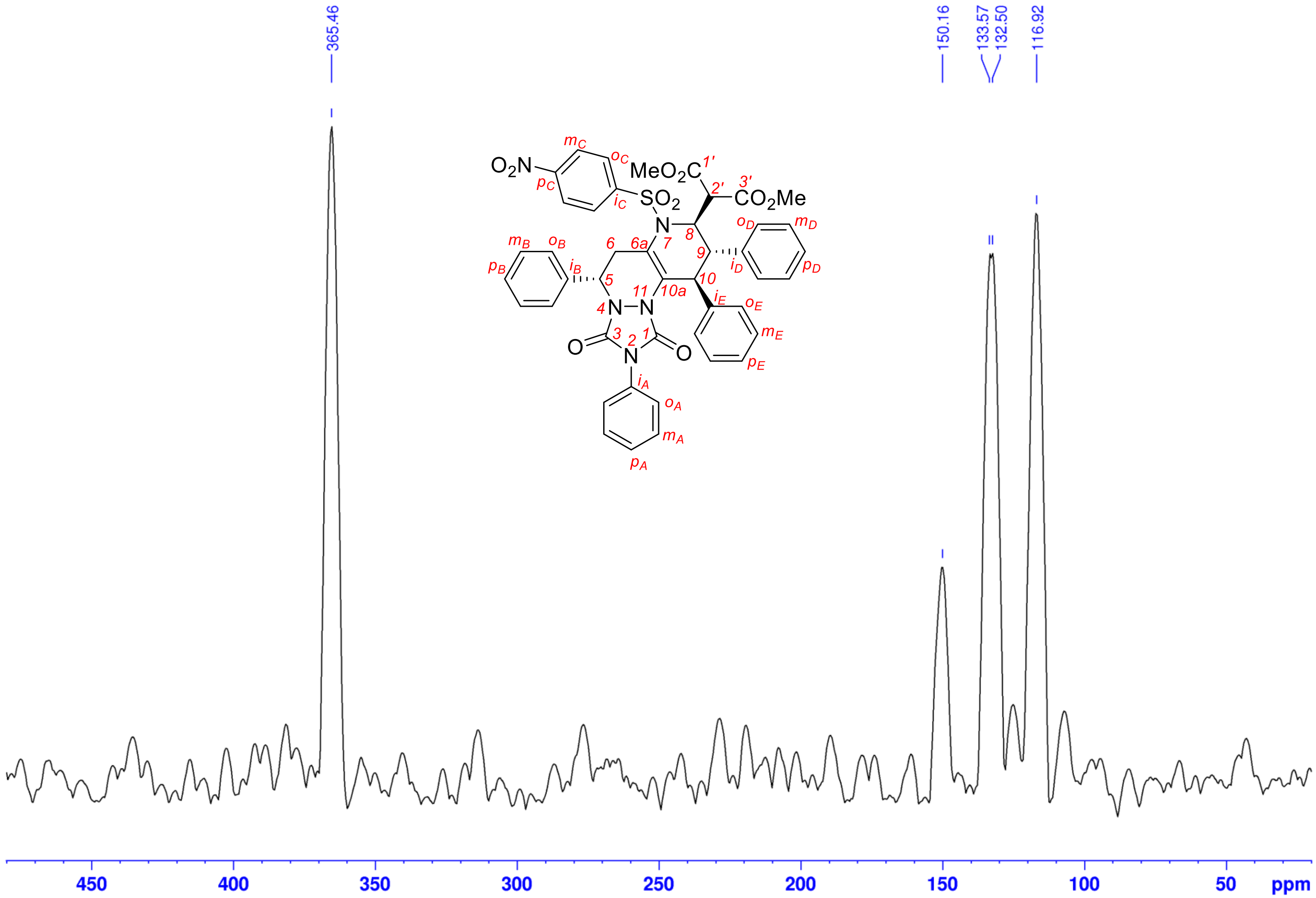

^{15}N NMR spectrum of **6** (30.4 MHz, $CDCl_3$)

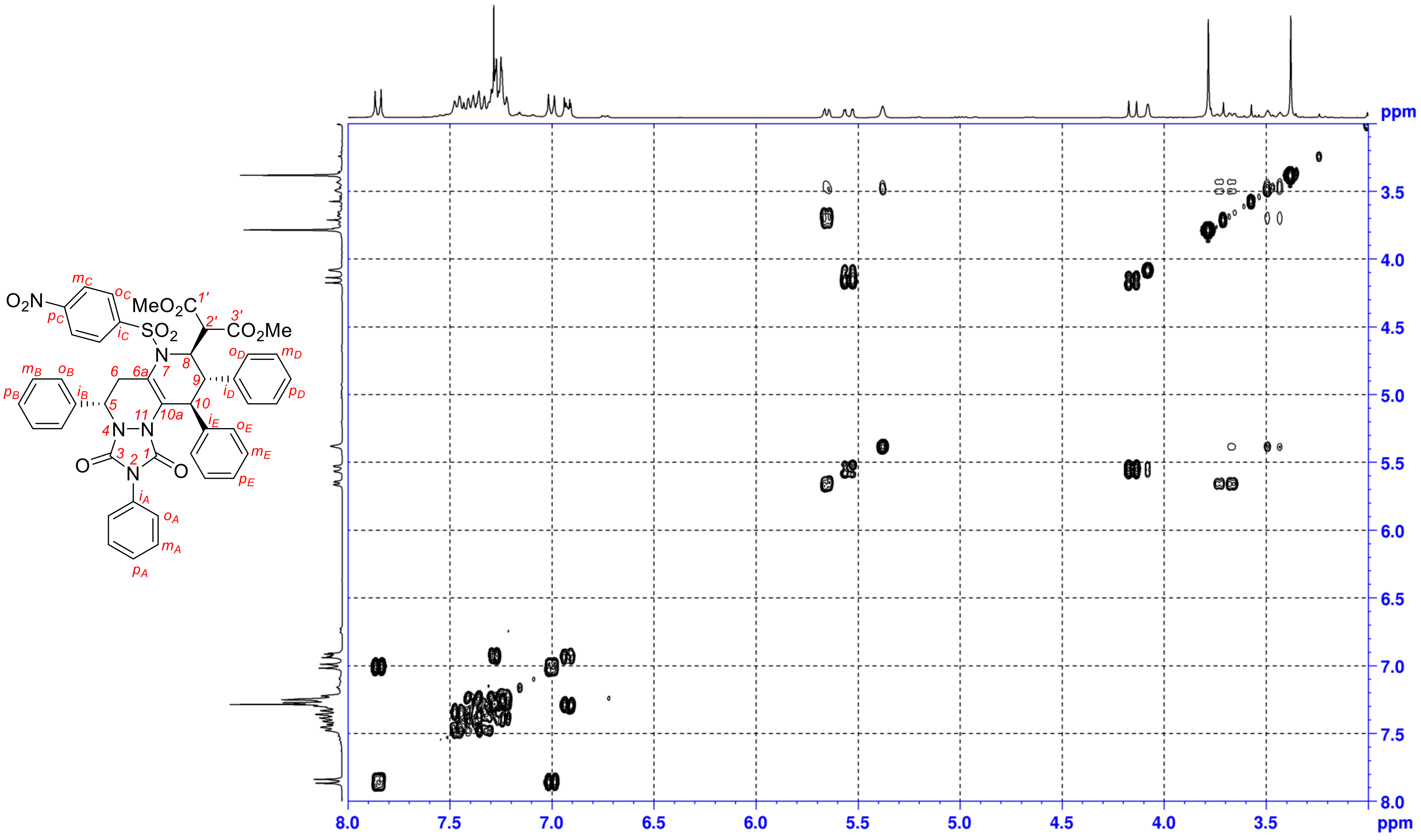

^{1}H,^{1}H-COSY NMR spectrum of **6** (300.1 MHz, $CDCl_3$)

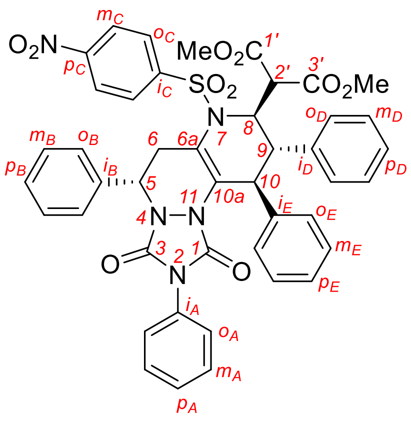

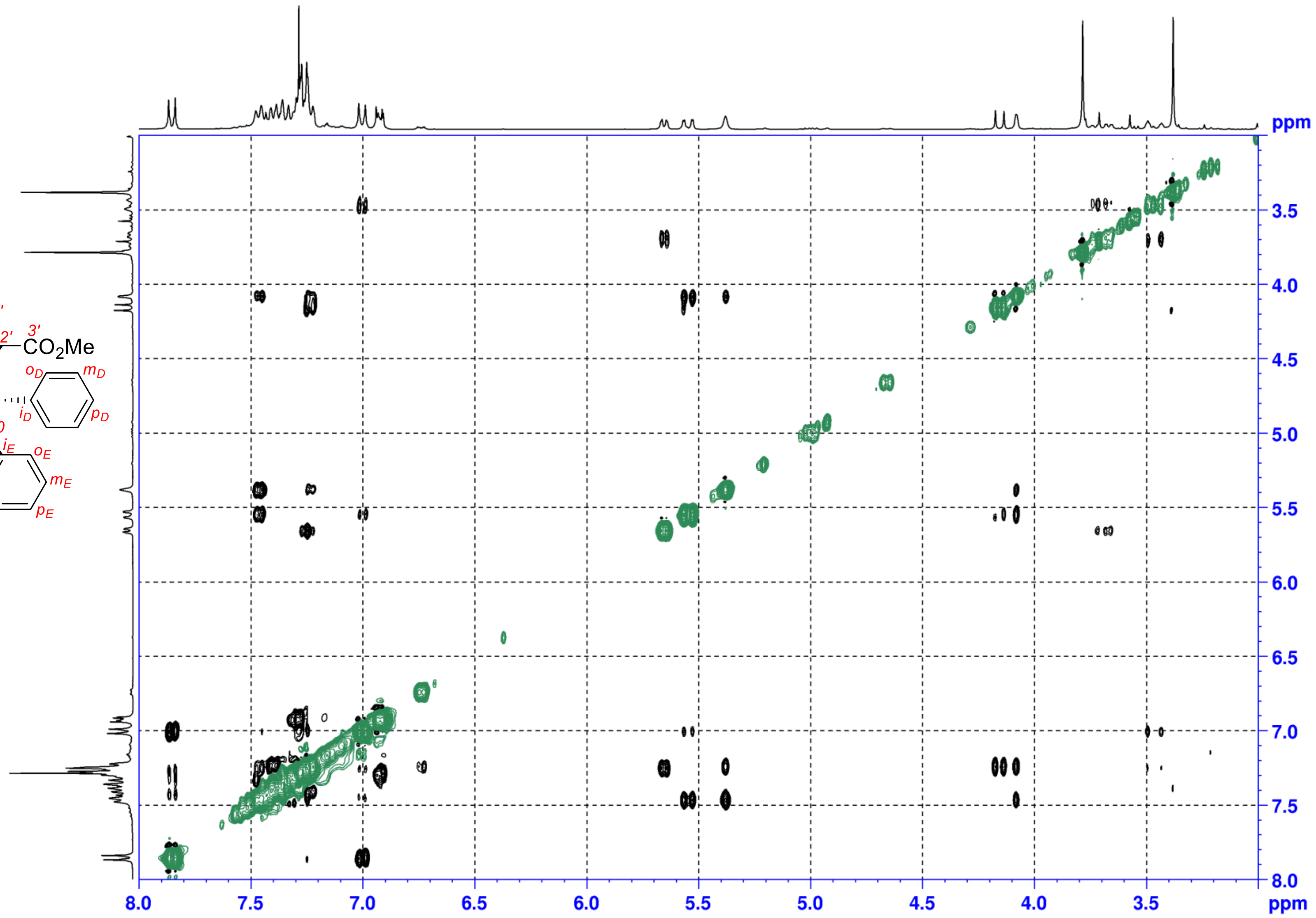

^{1}H,^{1}H-NOESY NMR spectrum of **6** (300.1 MHz, $CDCl_3$)

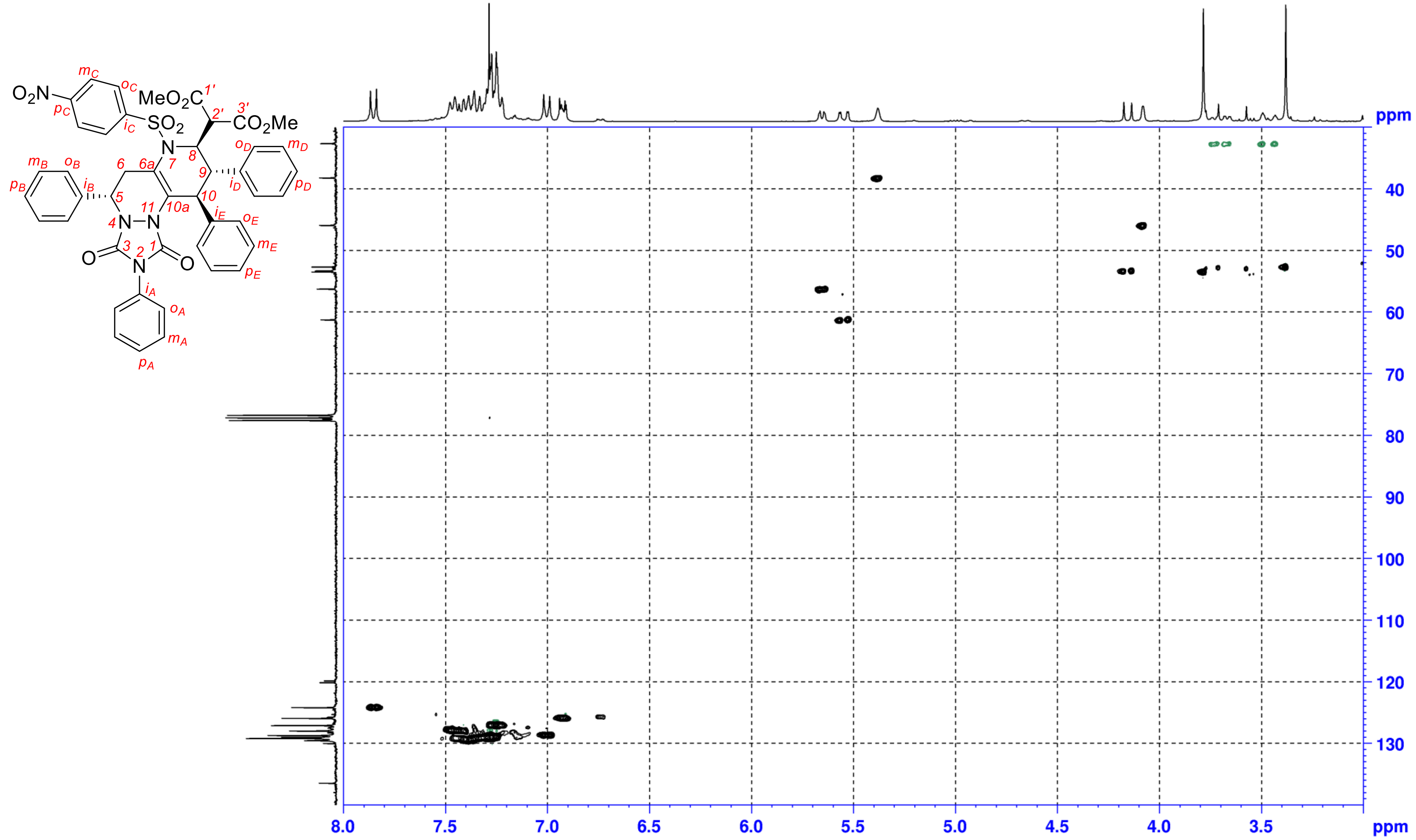

$^1H,^{13}C$-HSQC NMR spectrum of **6** (^1H: 300.1 MHz; ^{13}C: 75.5 MHz; $CDCl_3$)

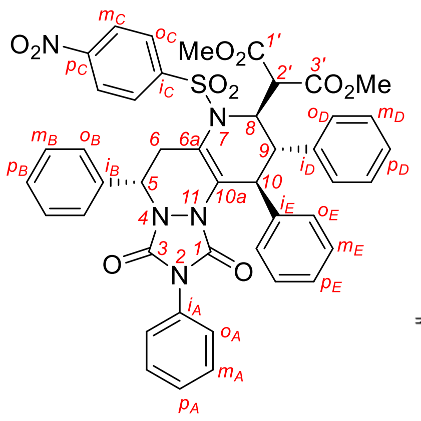

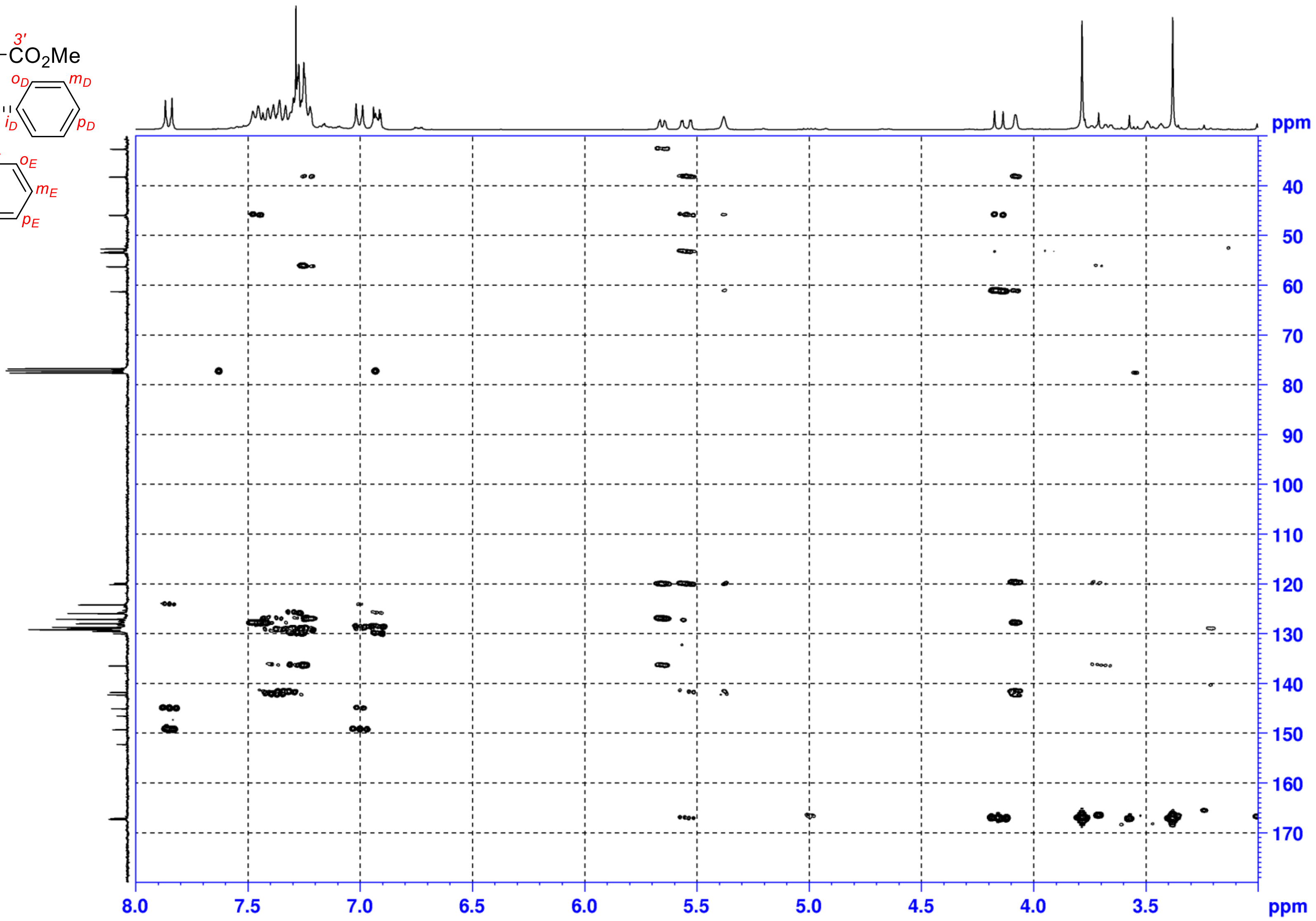

$^{1}H,^{13}C$-HMBC NMR spectrum of **6** (^{1}H: 300.1 MHz; ^{13}C: 75.5 MHz; $CDCl_3$)

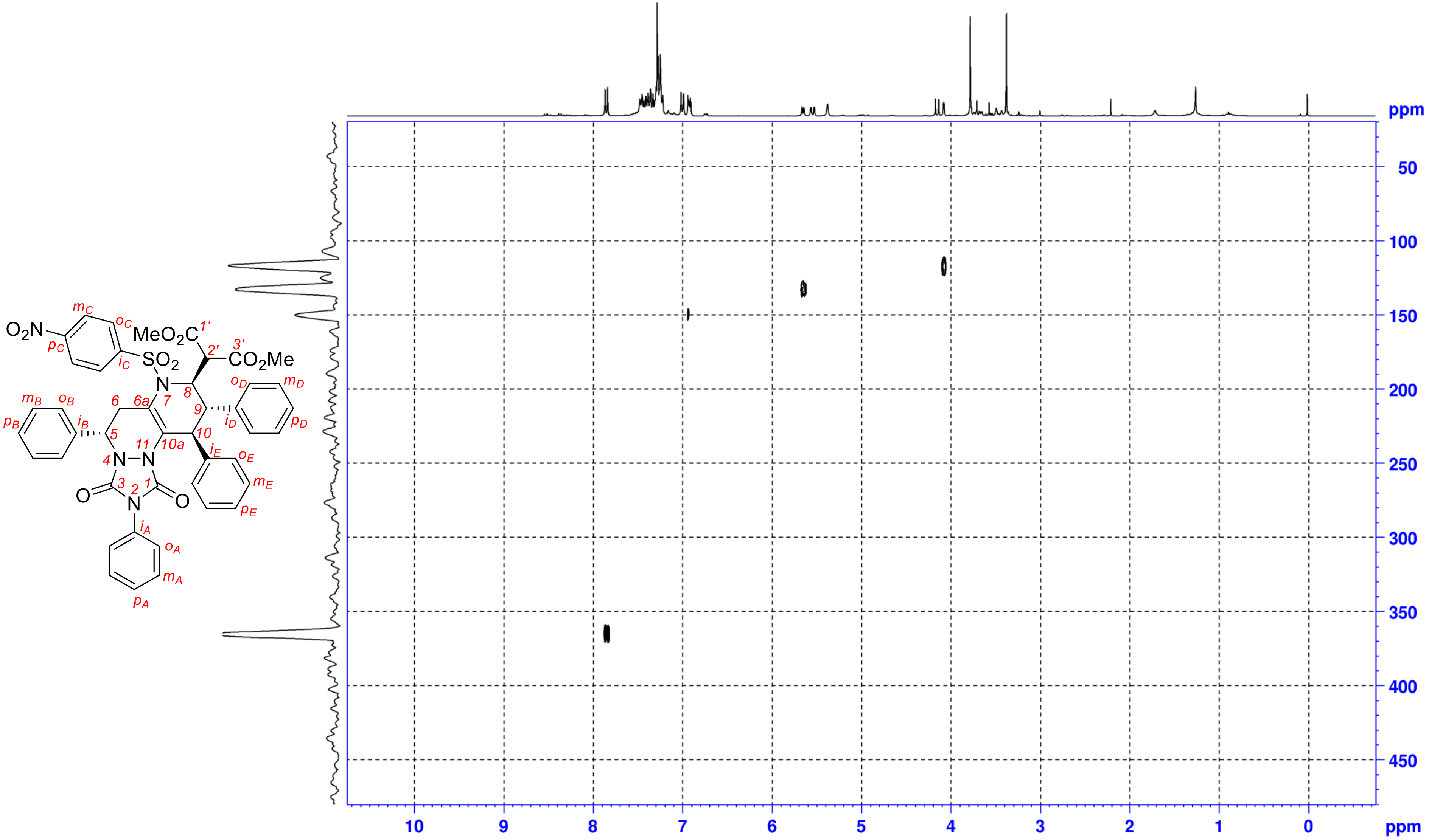

$^{1}H,^{15}N$-HMBC NMR spectrum of **6** (^{1}H: 300.1 MHz; ^{15}N: 30.4 MHz; $CDCl_3$)

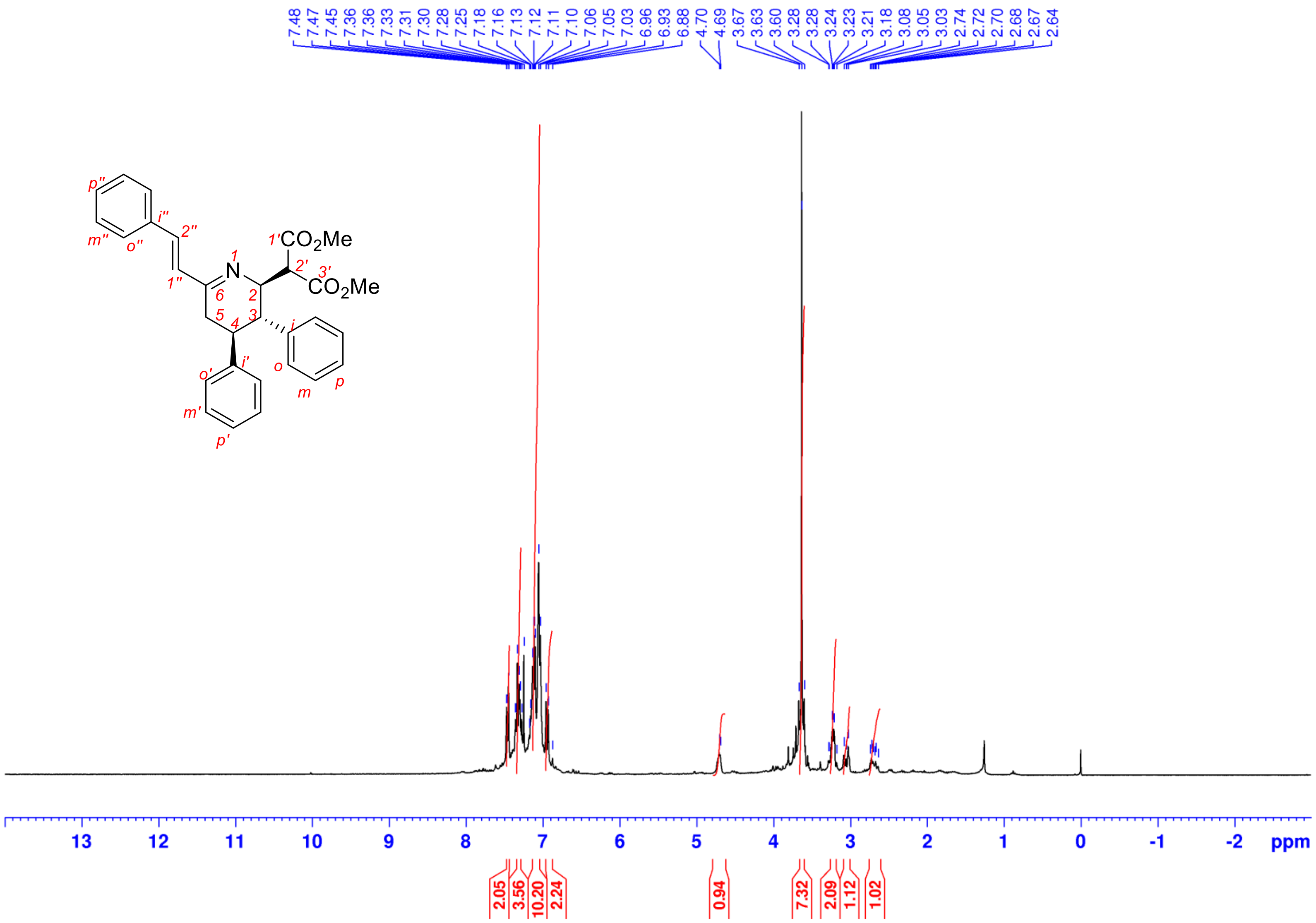

^{1}H NMR spectrum of **4** (300.1 MHz, $CDCl_3$)

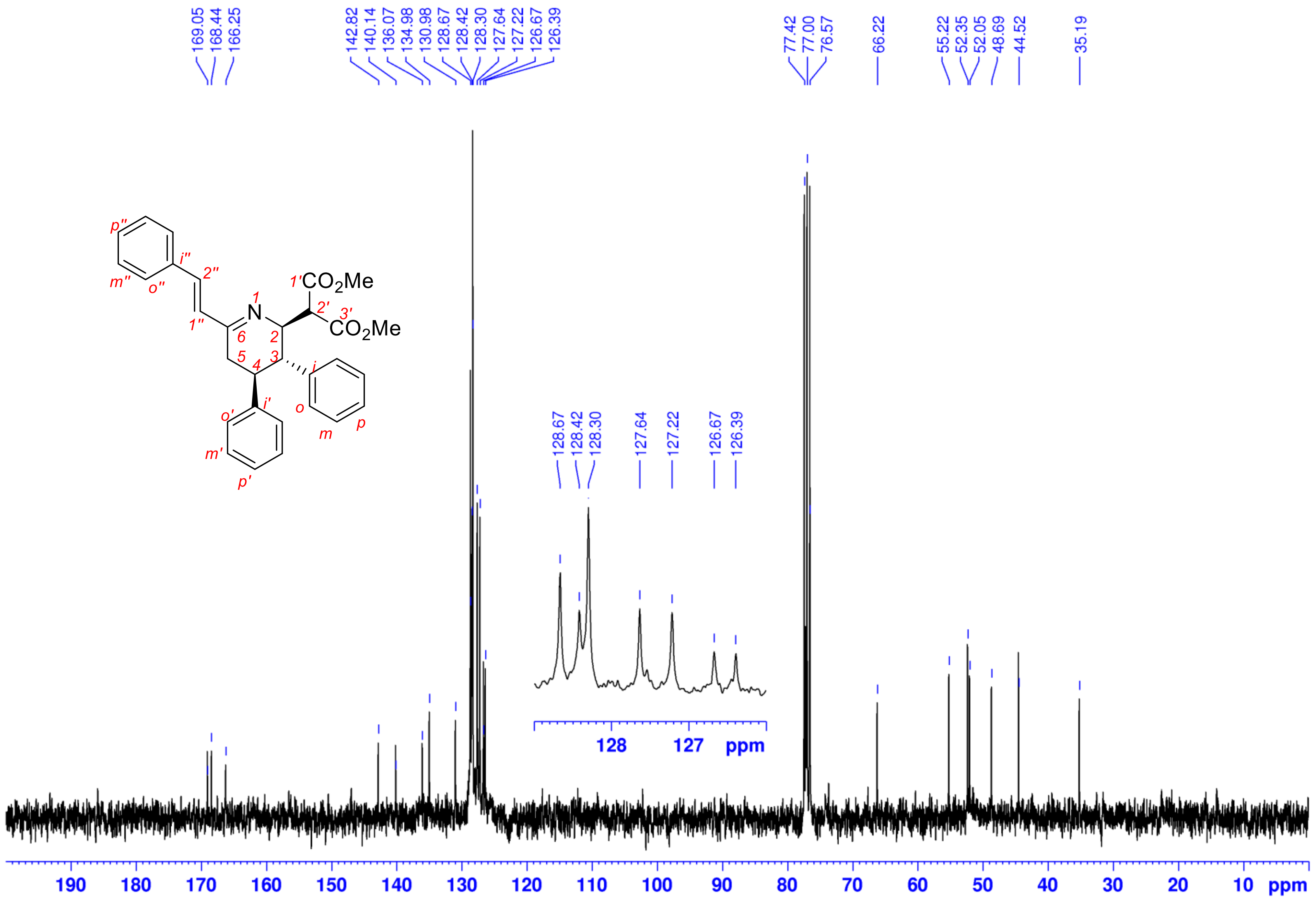

^{13}C NMR spectrum of **4** (75.5 MHz, $CDCl_3$)

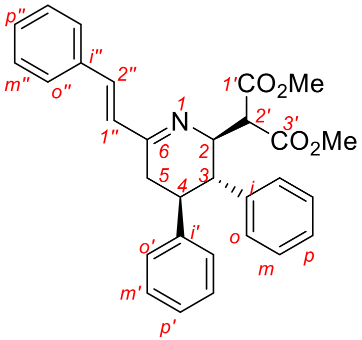

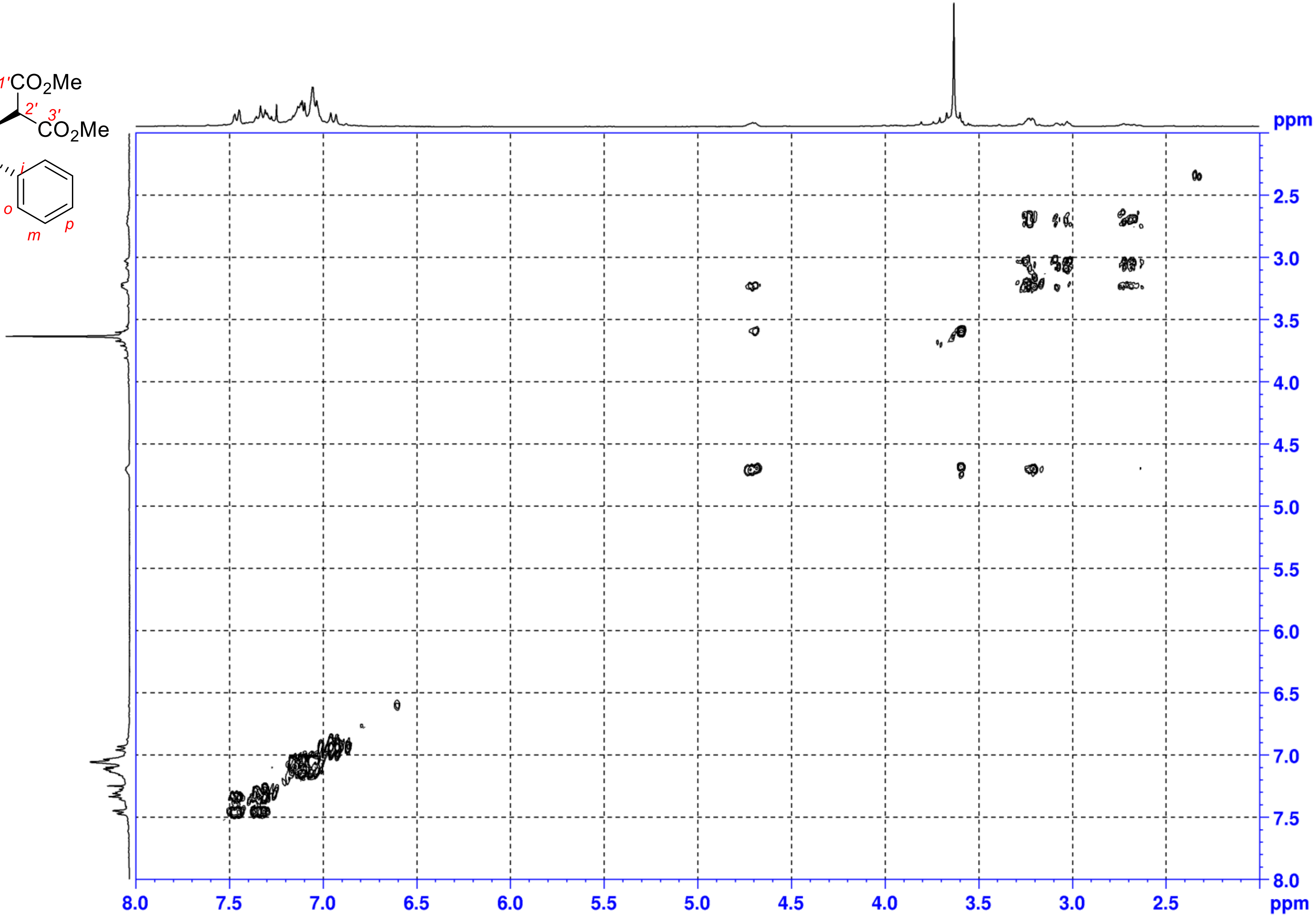

$^{1}H,^{1}H$-COSY NMR spectrum of **4** (300.1 MHz, $CDCl_3$)

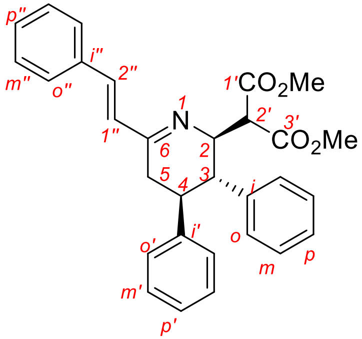

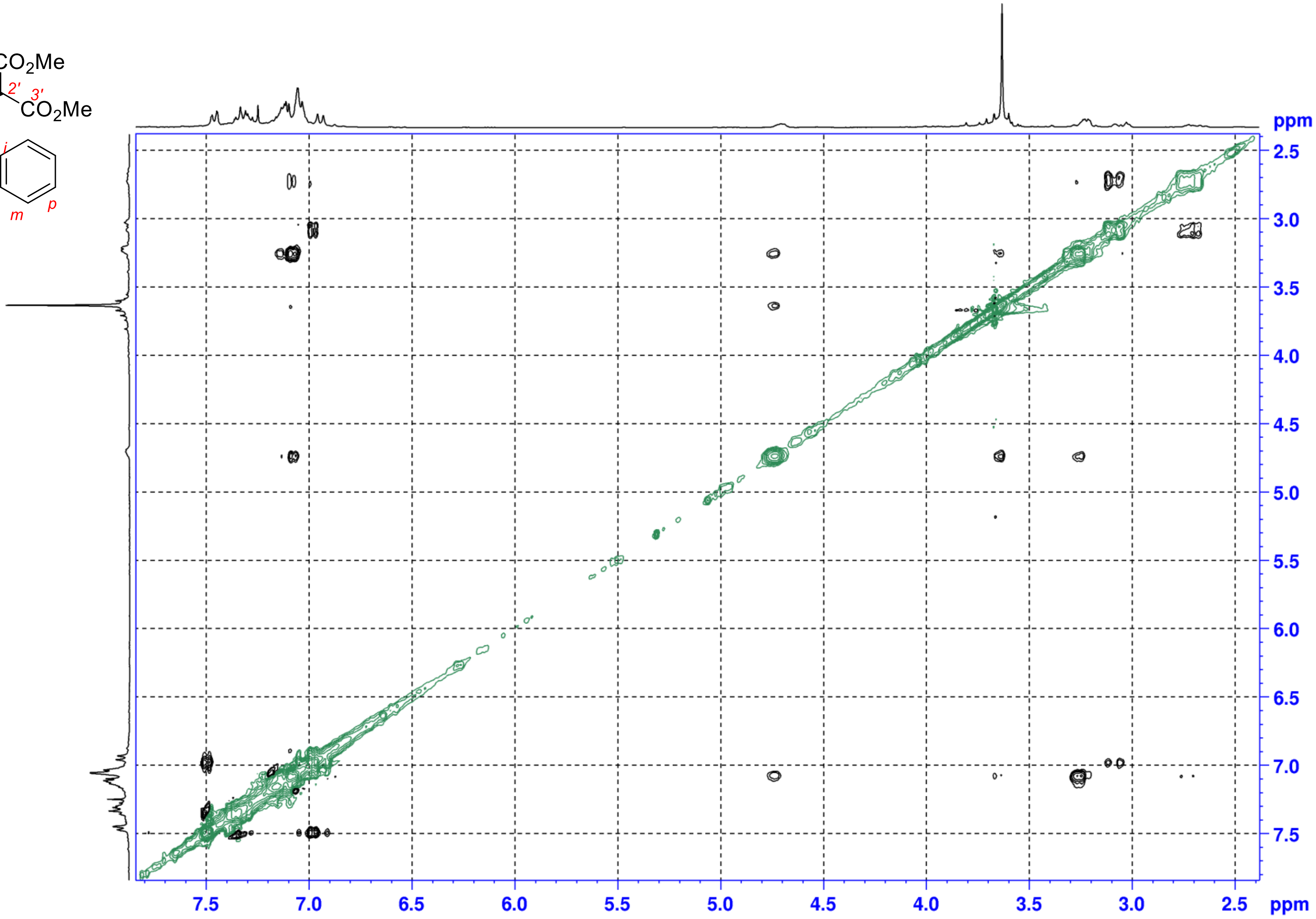

$^1H,^1H$-NOESY NMR spectrum of **4** (300.1 MHz, $CDCl_3$)

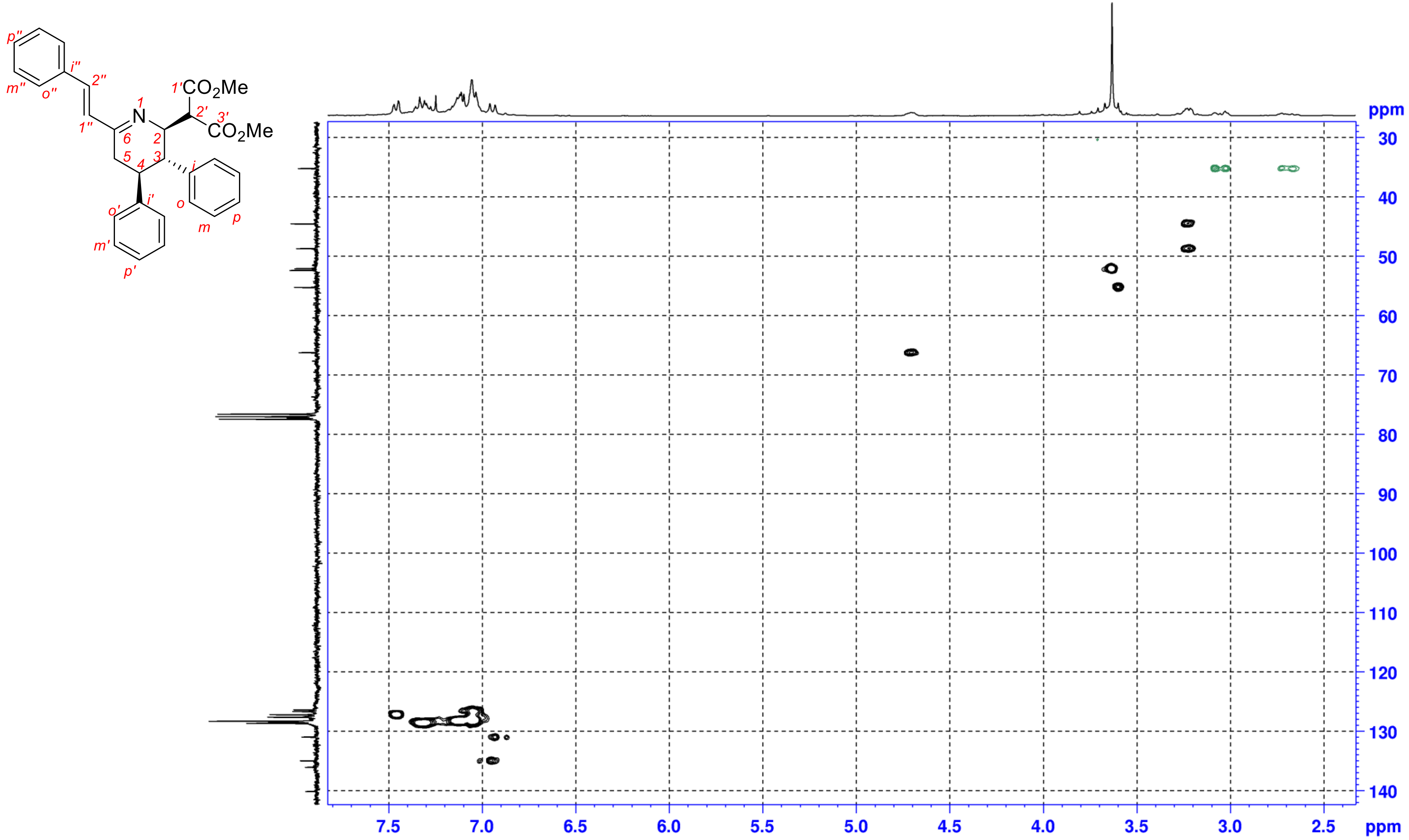

$^{1}H,^{13}C$-HSQC NMR spectrum of **4** (^{1}H: 300.1 MHz; ^{13}C: 75.5 MHz; $CDCl_3$)

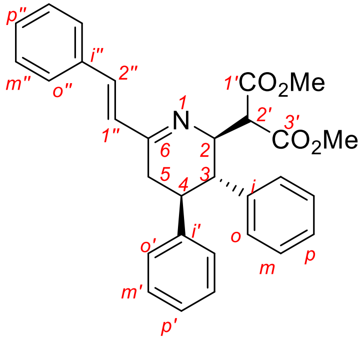

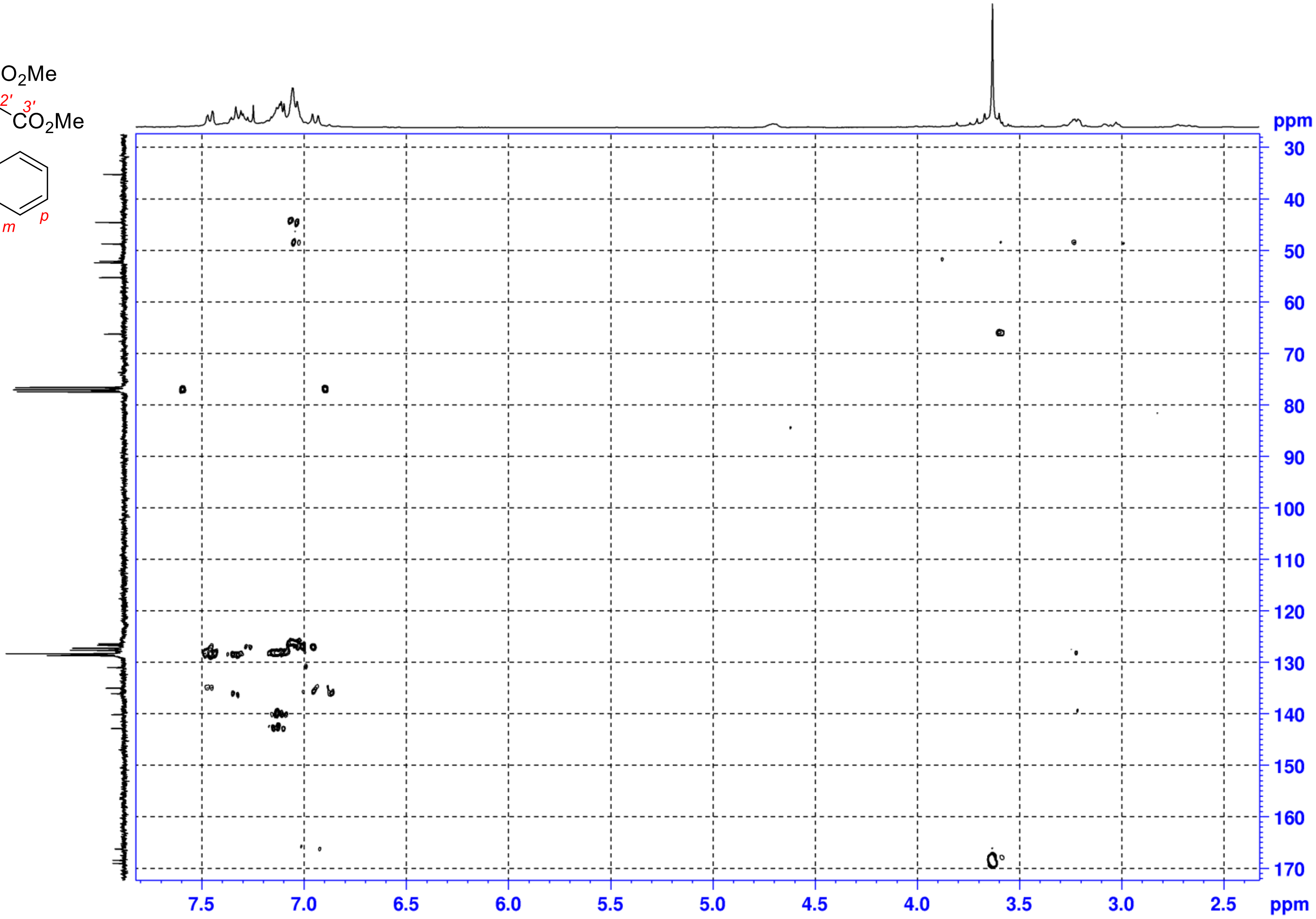

$^{1}H,^{13}C$-HMBC NMR spectrum of **4** (^{1}H: 300.1 MHz; ^{13}C: 75.5 MHz; $CDCl_3$)

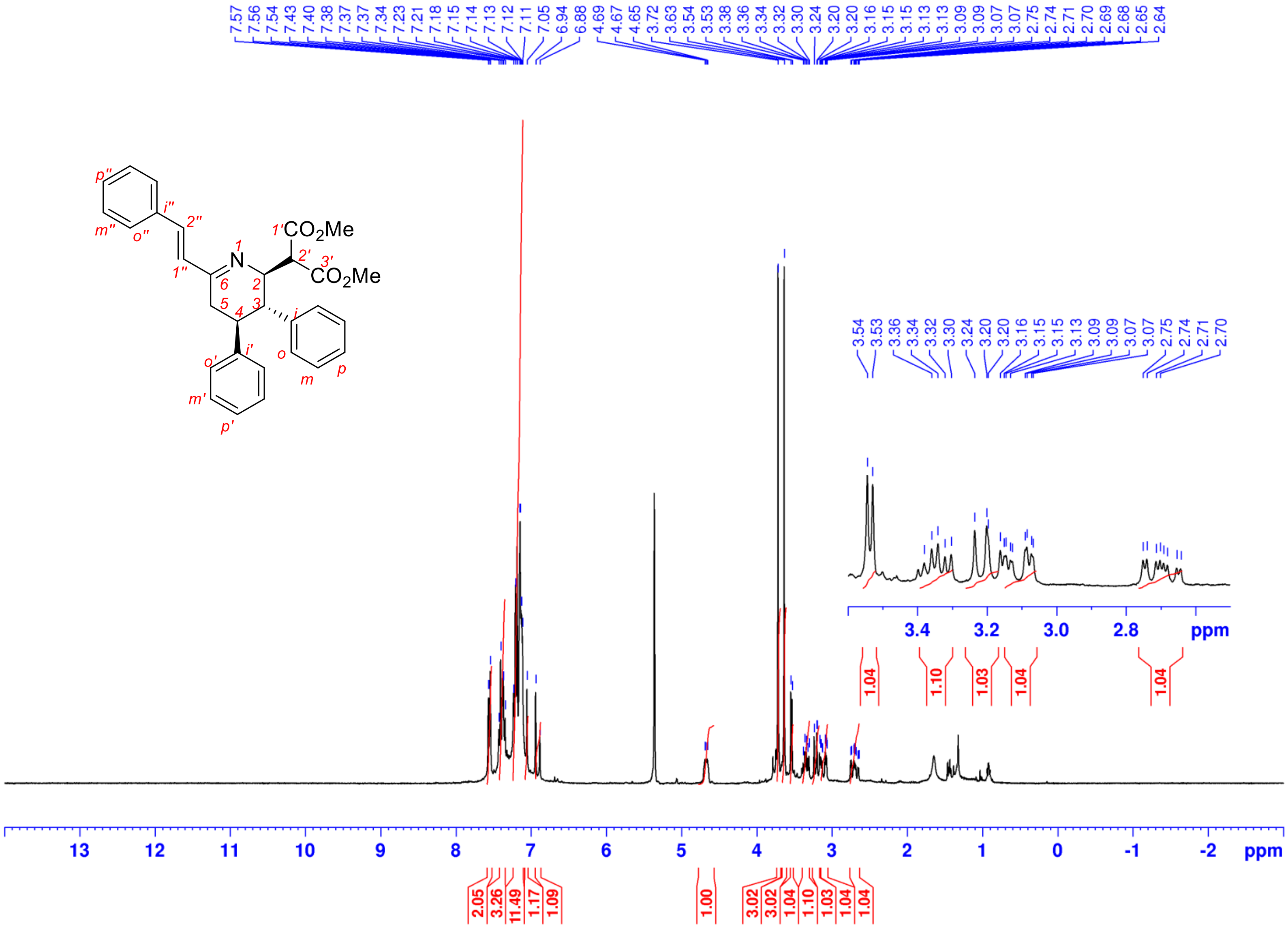

^{1}H NMR spectrum of **4** (300.1 MHz, CD_2Cl_2)

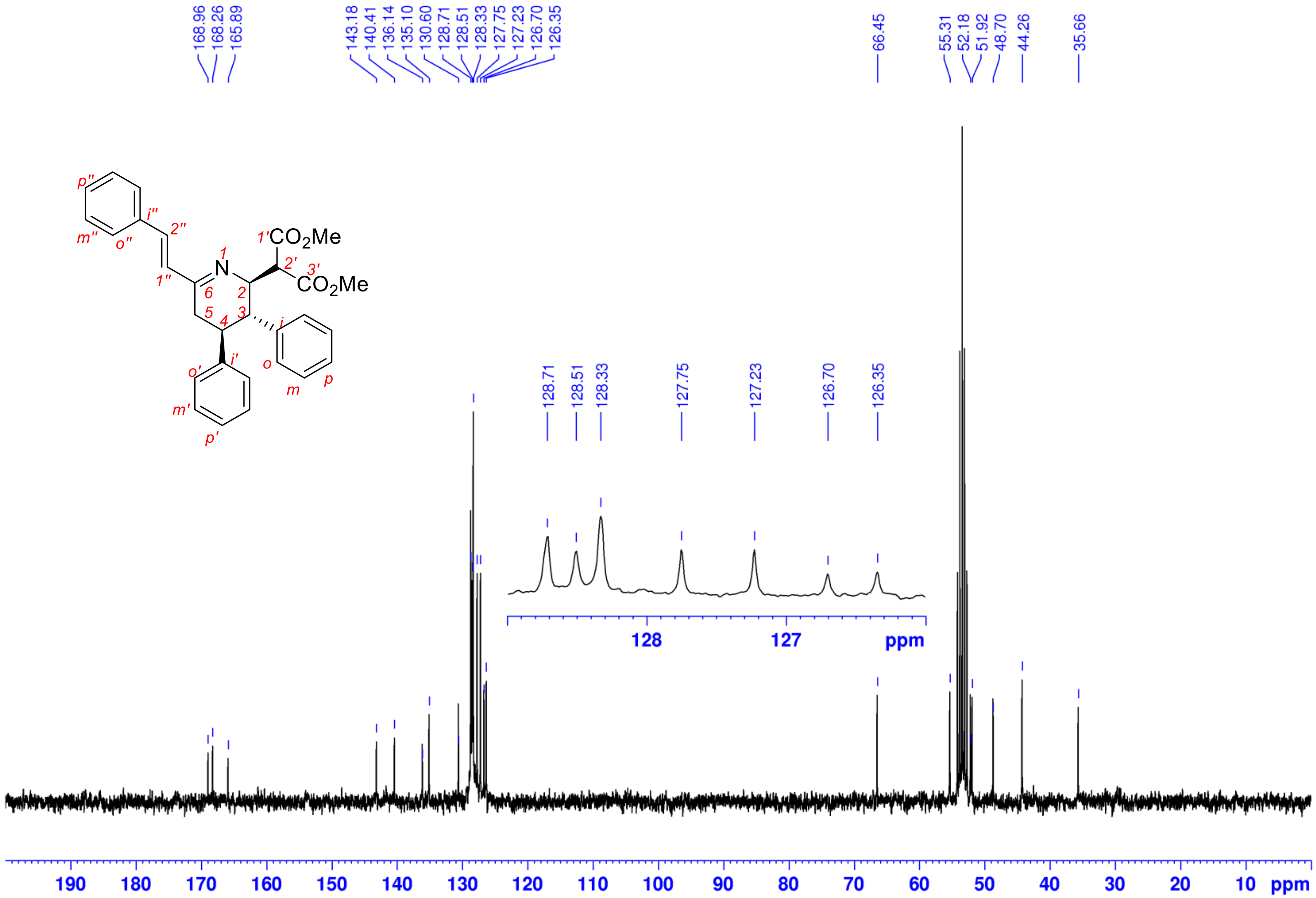

^{13}C NMR spectrum of **4** (75.5 MHz, CD_2Cl_2)

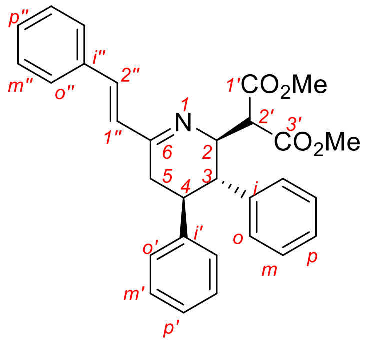

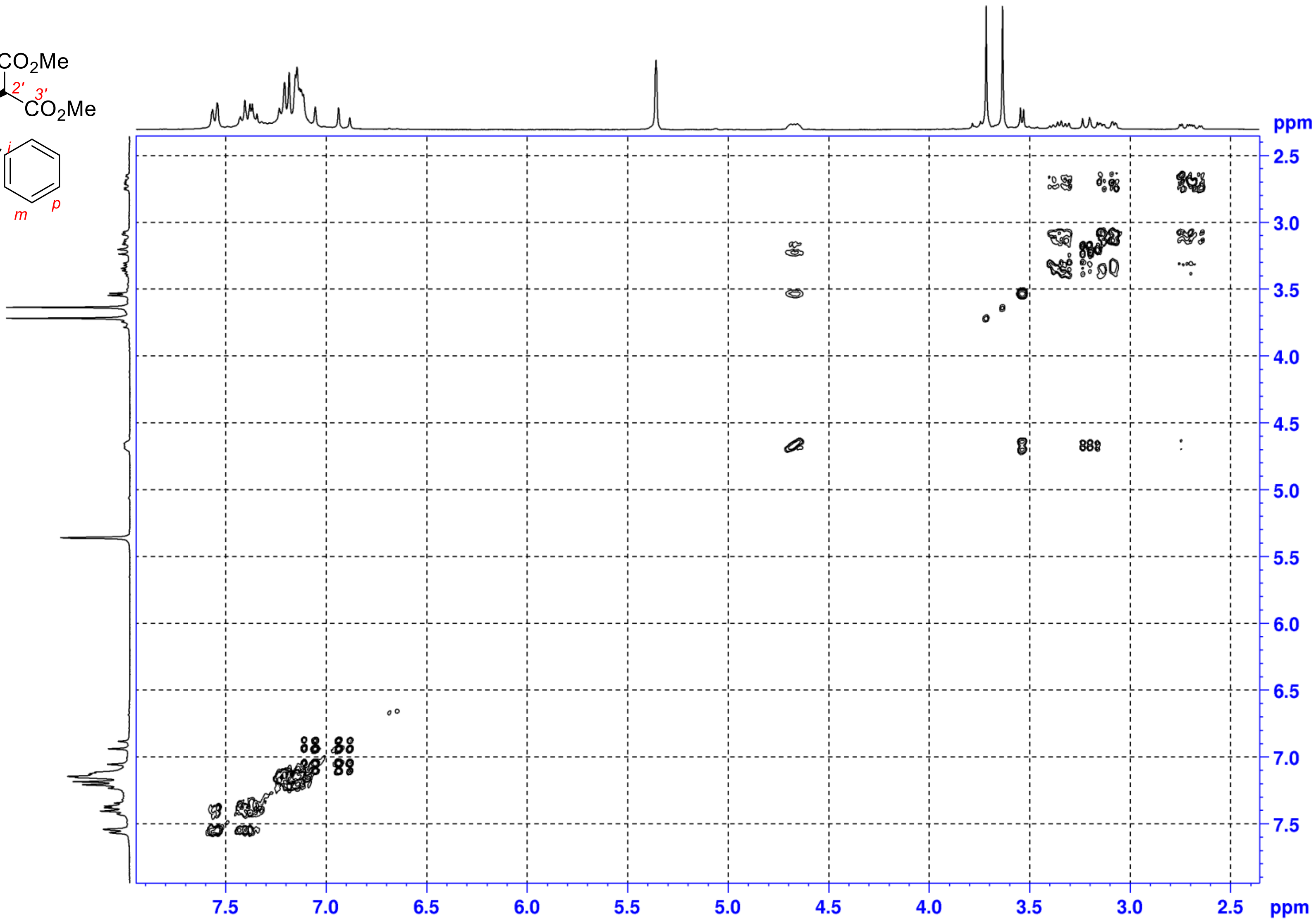

^{1}H,^{1}H-COSY NMR spectrum of **4** (300.1 MHz, CD_2Cl_2)

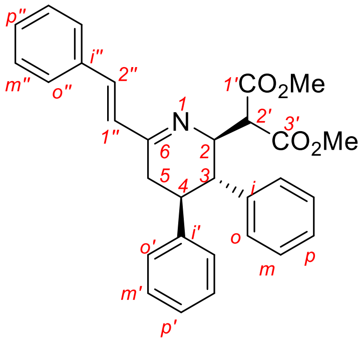

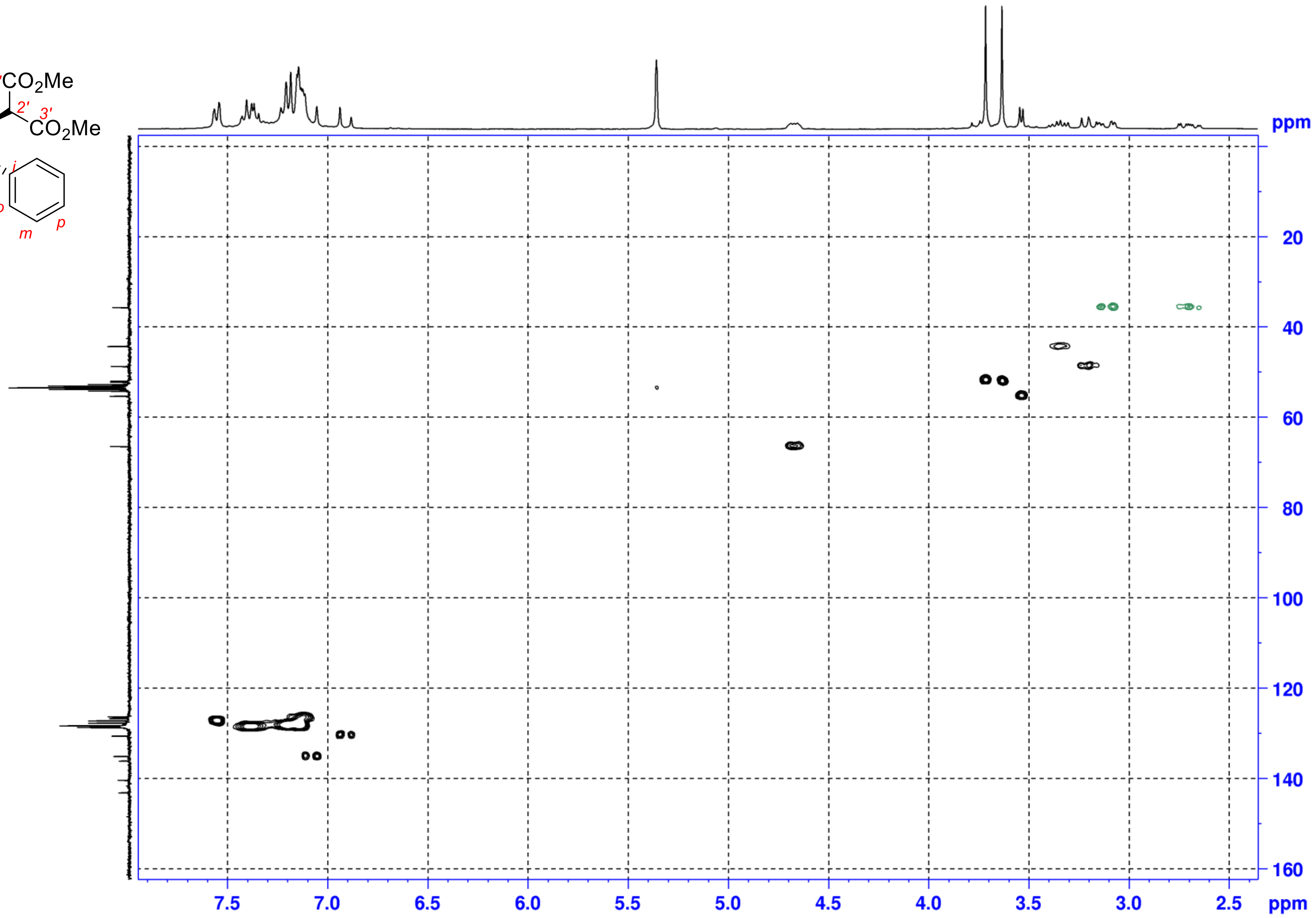

$^{1}H,^{13}C$-HSQC NMR spectrum of **4** (^{1}H: 300.1 MHz; ^{13}C: 75.5 MHz; CD_2Cl_2)

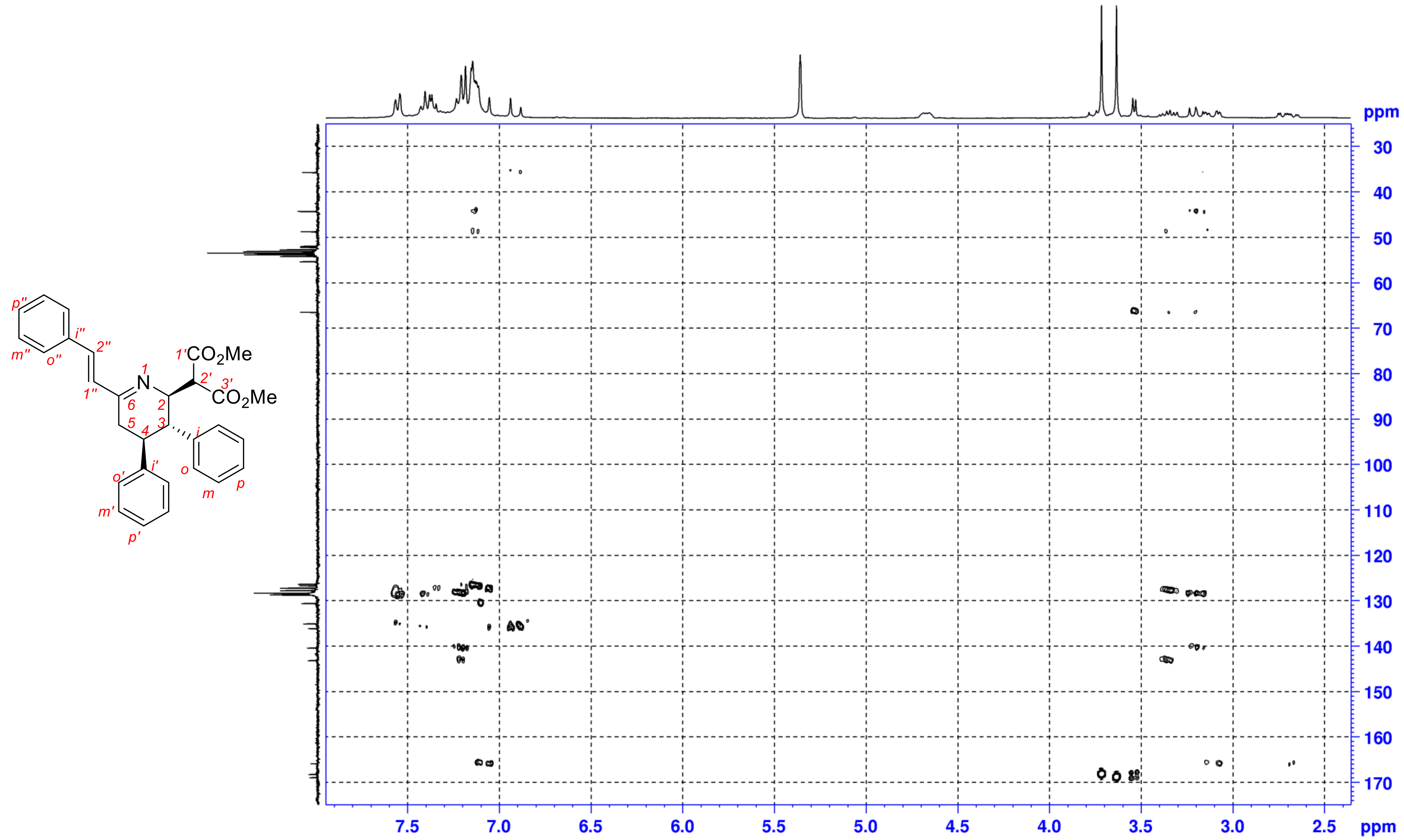

^{1}H,^{13}C-HMBC NMR spectrum of **4** (^{1}H: 300.1 MHz; ^{13}C: 75.5 MHz; CD_2Cl_2)
